# Atom-efficient arylation of *N*-tosylimines mediated by cooperative ZnAr_2_/Zn(C_6_F_5_)_2_ combinations†

**DOI:** 10.1039/d3cc01490h

**Published:** 2023-05-15

**Authors:** Andryj M. Borys, Tim Kunzmann, Jose M. Gil-Negrete, Eva Hevia

**Affiliations:** a Departement für Chemie, Biochemie und Pharmazie, Universität Bern Bern 3012 Switzerland eva.hevia@unibe.ch

## Abstract

By combining the Lewis acid Zn(C_6_F_5_)_2_ with nucleophilic diarylzinc (ZnAr_2_) reagents, we report the atom-efficient arylation of *N*-tosylimines under mild conditions. Mechanistic studies through the isolation of key intermediates reveal how the two zinc species act cooperatively to activate the imine substrate and regenerate the ZnAr_2_ reagent, enabling a limiting 50 mol% to be employed.

## Abstract 2

Operating *via* a special type of Zn/Zn′ cooperation, an atom-efficient method for the arylation of *N*-tosylimines has been developed combining nucleophilic ZnAr_2_ with Lewis acidic Zn(C_6_F_5_)_2_.

## Graphical abstract

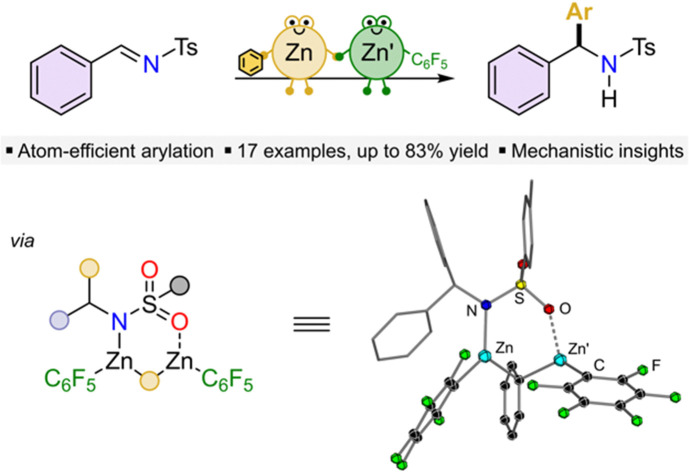

The addition of polar organometallics to unsaturated substrates represents a versatile method to build molecular complexity in a simple step.^1^ Exemplified by the Grignard reaction,^2^ which involves the addition of an organomagnesium halide to a carbonyl functionality, these reactions are widely used to construct new C–C bonds and obtain functionalised alcohol products.^3,4^ The analogous addition of organozinc halides to carbonyls was recognised as early as 1887 by Reformatsky^5^ and takes advantage of the lower nucleophilicity of zinc to enable the reaction to occur even in the presence of ester functional groups.^6^ Indeed, the high functional group tolerance and selectivity of organozinc reagents gives them widespread applications in organic synthesis,^7,8^ most notably in Negishi cross-coupling reactions.^9^ Nevertheless, the high selectivity of organozinc reagents comes with the drawback of reduced reactivity, meaning that transition-metal catalysts, additives, or harsh reaction conditions are often needed to facilitate C–C bond formation when using these reagents.^7,8^

To overcome these limitations, we have previously exploited the use of diarylzinc reagents in combination with the Lewis acidic species Zn(C_6_F_5_)_2_ to enable the atom-efficient functionalisation of glycosyl bromides^10^ or *N*,*O*-acetals^11^ without the need for transition-metal catalysts. Key for the success of these approaches is the lack of co-complexation or ligand scrambling between both types of Zn reagents. We thus considered whether this same Zn/Zn combination could instead be employed for nucleophilic addition reactions and focused our attention towards the functionalisation of imines to give amine products. Whilst the addition of organolithiums to imines occurs readily,^12^ and can even be conducted under aerobic conditions,^13,14^ additives or catalysts are generally needed when employing less nucleophilic Grignard or organozinc reagents. For example, Lewis acid additives such as Me_3_SiOTf (OTf = OSO_2_CF_3_) facilitate the addition of Grignard reagents to imines,^15^ whilst copper catalysts are needed for the addition of organozinc reagents to *N*-tosylimines.^16^ In many cases, a large excess of the organometallic reagent is employed,^16,17^ and these processes have not yet been optimised for arylzinc species.

We first explored the reaction of *N*-(tosyl)-4-fluorobenzylideneamine (1) with various phenyl-zinc reagents under different conditions (Table 1).

**Table 1:** Reaction optimisation. Yields refer to spectroscopic yields measured using ferrocene as an internal standard. Ar = 4-F–C_6_H_4_; Ts = SO_2_–C_6_H_4_–CH_3_

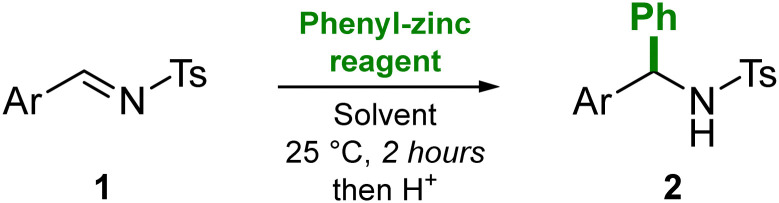				
Entry	Phenyl-zinc reagent	Equivalents used	Solvent	Yield (%)
1	ZnPh_2_	1	THF	12
2	ZnPh_2_	1	Toluene	80
3	ZnPh_2_	0.5	Toluene	45
**4**	**ZnPh**_**2**_ + **Zn**(**C**_**6**_**F**_**5**_)_**2**_	**0.5 each**	**Toluene**	**80**
5	PhZnBr	1	THF	0
6	PhZnBr	1	Toluene	0
7	Ph_3_ZnLi	1	THF	70

When using 1 equivalent of ZnPh_2_ in THF, only 12% yield of *N*-tosyldiarylmethanamine (2) was obtained after quenching the reaction (entry 1). Contrastingly, performing the reaction in toluene gave an 80% yield of 2 (entry 2). Numerous reports have demonstrated that non-donor solvents work best for nucleophilic substitution reactions involving diarylzinc reagents.^10,11,18^ This solvent dependence suggests that the pre-coordination of the imine substrate to the Lewis acidic zinc centre plays an essential role in the reaction (*vide infra*). Lowering the equivalents of ZnPh_2_ to 0.5, only 45% yield of 2 was obtained, indicating that only one phenyl-substituent from ZnPh_2_ is transferred. Using an equimolar amount of Zn(C_6_F_5_)_2_ however, restored the high yields (80%, entry 4), with no competitive transfer of the C_6_F_5_-substituent observed, allowing for the efficient transfer of both Ph groups to the substrate. Contrastingly, no product was obtained when using PhZnBr, regardless of the solvent employed (entries 5 and 6), reflecting the decreased nucleophilicity of organozinc halides compared to diorganozinc reagents. 70% yield of 2 was observed when using anionically activated lithium zincate,^19^ Ph_3_ZnLi (entry 7), but here only one phenyl-substituent is transferred and therefore this reagent simply acts as a PhLi surrogate (see ESI† for extended reaction optimisation table).

Having established that 0.5 equivalents each of ZnPh_2_ and Zn(C_6_F_5_)_2_ gave the optimal conditions, we went on to explore the scope of different diarylzinc reagents for the nucleophilic addition reaction to *N*-(tosyl)-benzylideneamine (1a) (Scheme 1). Good to high yields of the corresponding addition products 2a–c were obtained when using 0.5 equivalents of ZnPh_2_, Zn(4-Me–C_6_H_4_)_2_ and Zn(4-OMe–C_6_H_4_)_2_ respectively. For less nucleophilic ZnAr_2_ species, slightly lower yields of 55% (2d) and 38% (2e) were obtained, but extended heating improved the yield of 2e up to 80%. *ortho*-Substituted diarylzincs gave good yields of 64% (2f) and 59% (2g) respectively. Remarkably, concerning the synthesis of 2g, no product was observed when using 1 equivalent of Zn(2-OMe–C_6_H_4_)_2_ in the absence of Zn(C_6_F_5_)_2_. This is attributed to the *ortho*-OMe-substituents that can coordinate and quench the Lewis acidity of the Zn centre,^20,21^ which has a similar detrimental role to using ethereal solvents (see Table 1). The sterically encumbered Zn(2,6-Me_2_-C_6_H_3_)_2_ gave a good yield of 65% (2h) albeit after heating at 80 °C for 20 hours. Electron-deficient Zn(1-naphthyl)_2_ gave a good yield of 68% (2i) whilst the heteroaryl Zn(2-thiophenyl)_2_ gave a high yield of 80% (2j). In general, the yields obtained when using 0.5 equivalents each of ZnAr_2_ and Zn(C_6_F_5_)_2_ were higher than when simply using 1 equivalent of ZnAr_2_ in the absence of Zn(C_6_F_5_)_2_, particularly for less nucleophilic ZnAr_2_ compounds (see Scheme S1 in the ESI† for full details). This methodology is therefore attractive when employing complex ZnAr_2_ species since it allows the atom-efficient transfer of both aryl-substituents, and furthermore, its high functional group tolerance and mild reaction conditions make this approach suitable for late state functionalisation strategies.

**Scheme 1:**
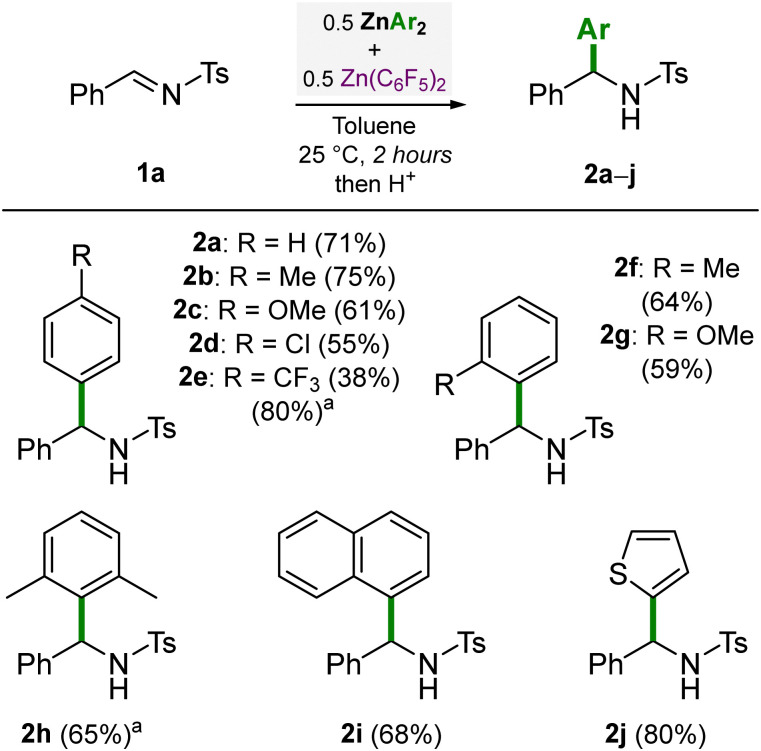
Diarylzinc scope for the nucleophilic addition reaction to *N*-(tosyl)-benzylideneamine (1a). Yields refer to isolated yields after column chromatography. ^a^ Heated to 80 °C for 20 hours.

We then went on to investigate the scope of different *N*-tosylimines (1a–p) using 0.5 equivalents each of ZnPh_2_ and Zn(C_6_F_5_)_2_ (Scheme 2). Modest to high yields (34–83%) of the corresponding addition products 2a–p were obtained under these reaction conditions. In general, electron-withdrawing substituents (F, Cl, NO_2_, CN) were found to give higher yields due to the increased electrophilicity at the imine carbon, whilst electron-donating substituents (OMe or NMe_2_) gave modest or low yields (*e.g.* 9% for 2l). The synthesis of compounds 2m, 2n and 2p exemplifies the high functional group tolerance of organozinc compounds, as these unsaturated substituents (NO_2_, CN and pyridyl) are typically incompatible with more nucleophilic polar organometallics such as organolithiums.^14,22,23^

**Scheme 2:**
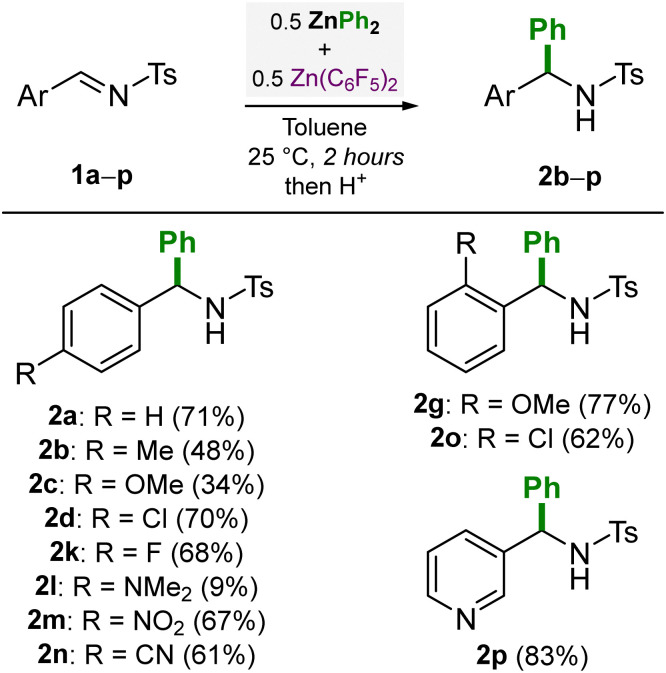
*N*-tosylimine scope for the nucleophilic addition of ZnPh_2_ in the presence of Zn(C_6_F_5_)_2_. Yields refer to isolated yields after column chromatography.

To understand how Zn(C_6_F_5_)_2_ facilitates the atom-efficient addition of diarylzinc reagents to *N*-tosylimines, a series of stoichiometric reactions were performed with each component (Fig. 1). The addition of 1 equivalent of ZnPh_2_ to *N*-(tosyl)-benzylideneamine (1a) in toluene affords the corresponding 1,2-addition product, 3a (Fig. 1a). Compound 3a exists a tetramer in the solid-state which oligomerises due to coordination of the sulfonamide oxygens to the Zn centres, forming a central eight-membered {ZnOSO}_2_ ring. Only one of the phenyl-substituents from ZnPh_2_ is transferred to the imine and the remaining phenyl-substituent on Zn occupies either a bridging or terminal position, akin to ZnPh_2_ itself which is dimeric in the solid-state in the absence of donors.^24^ The addition of TMEDA (*N*,*N*,*N*′,*N*′-tetramethylethylenediamine) to 3a leads to deaggregation to give the monomeric and solvated addition product 3a.TMEDA (see ESI† for the solid-state structure).

**Fig. 1:**
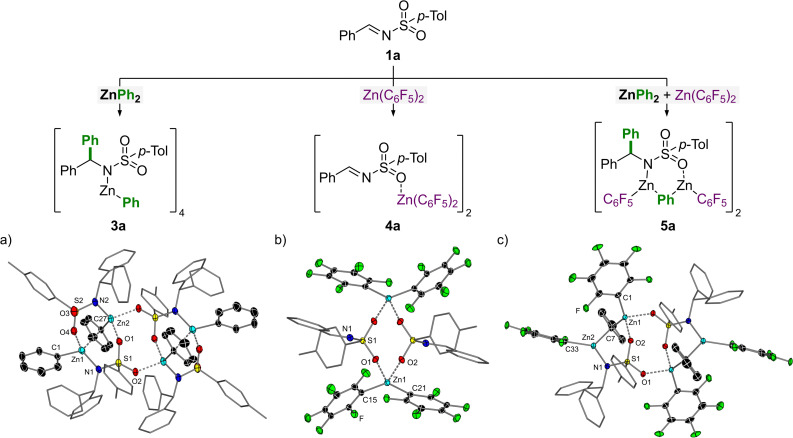
Stoichiometric reactions of *N*-(tosyl)-benzylideneamine (1a) with 1 equivalent of ZnPh_2_ and/or Zn(C_6_F_5_)_2_ in toluene. (a) Molecular structure of 3a. Thermal ellipsoids shown at 30% probability. Hydrogen atoms omitted and aryl-substituents not on Zn shown as wireframes for clarity. Selected bond lengths [Å]: Zn1–C1 1.983(3); Zn1⋯C27 2.364(3); Zn1–N1 2.006(3); O4⋯Zn1 2.122(2); Zn2–C27 2.003(3); Zn2–N2 1.965(2); O1⋯Zn2 2.112(2); O2⋯Zn2 2.033(2). Selected bond angles [°]: C1–Zn1–N1 127.1(1); N2–Zn2–C27 124.4(1). (b) Molecular structure of 4a. Thermal ellipsoids shown at 30% probability. Hydrogen atoms omitted and aryl-substituents not on Zn shown as wireframes for clarity. Selected bond lengths [Å]: Zn1–C15 1.974(5); Zn1–C21 1.987(5); O1⋯Zn1 2.200(3); O2⋯Zn1 2.129(2). Selected bond angles [°]: C15–Zn1–C21 138.8(2). (c) Molecular structure of 5a. Thermal ellipsoids shown at 30% probability. Hydrogen atoms omitted and aryl-substituents not on Zn shown as wireframes for clarity. Selected bond lengths [Å]: Zn1–C1 1.989(2); Zn1–C7 2.058(2); O1⋯Zn1 2.055(1); O2⋯Zn1 2.066(1); Zn2–C7 2.111(2); Zn2–C33 1.983(2); Zn2–N1 1.995(1). Selected bond angles [°]: C1–Zn1–C7 131.41(7); Zn1–C7–Zn2 90.51(6); C7–Zn2–C33 118.22(7); C7–Zn2–N1 115.27(6); N1–Zn2–C33 126.50(6).

The addition of 1 equivalent of Zn(C_6_F_5_)_2_ to *N*-(tosyl)-benzylideneamine (1a) in toluene affords the corresponding 1 : 1 Lewis adduct, 4a (Fig. 1b). Compound 4a is dimeric in the solid-state and shows coordination of the sulfonamide oxygens to the Lewis acidic Zn centres forming a central eight-membered {ZnOSO}_2_ ring, akin to 3a. This coordination serves to increase the electron-withdrawing capacity of the tosyl-substituent which in turn increases the electrophilicity of the imine carbon and thus facilitates nucleophilic addition of the diarylzinc species. Compound 4a shows comparable structural parameters (O⋯Zn and C_aryl_–Zn distances; C_aryl_–Zn–C_aryl_ angles) to other reported Lewis adducts such as (THF)_2_Zn(C_6_F_5_)_2_.^25^

Finally, the combination of benzylideneamine (1a) with 1 equivalent each of ZnPh_2_ and Zn(C_6_F_5_)_2_ affords compound 5a in which all three reaction components are incorporated (Fig. 1c). This compound can also be accessed by treating 3a with Zn(C_6_F_5_)_2_, or 4a with ZnPh_2_. Compound 5a is dimeric in the solid-state and bears similar structural properties to 3a. The two unique Zn environments each bear one terminal C_6_F_5_-substituent and share a bridging phenyl-substituent. Oligomerisation in the solid-state is again caused by coordination of the sulfonamide oxygens to the Lewis acidic Zn centres to form a central eight-membered {ZnOSO}_2_ ring, which is a common structural feature in compounds 3a, 4a and 5a.

Based on these stoichiometric studies and our previous work employing ZnAr_2_ and Zn(C_6_F_5_)_2_ for nucleophilic substitution reactions with *N*,*O*-acetals,^11^ a mechanism for this transformation can be proposed (Scheme 3). The addition of ZnPh_2_ and Zn(C_6_F_5_)_2_ to the *N*-tosylimine substrates affords compound 5 (likely *via*3a or 4a), as shown in Fig. 1. Zn(C_6_F_5_)_2_ acts as a strong Lewis acid to activate the imine substrate, as illustrated in compound 4a, and justifies why non-coordinating solvents (toluene *vs.* THF) are necessary for the transformation. We have previously demonstrated that the addition of Zn(C_6_F_5_)_2_ to heteroleptic intermediate RZnPh (where R = OMe for *N,O*-acetals; R = NR_2_ for 3a) results in the formation of RZn(C_6_F_5_) alongside “PhZn(C_6_F_5_)”.^11^ The latter species “PhZn(C_6_F_5_)” however dissociates into its favoured homoleptic components [*e.g.* 0.5 equivalents each of ZnPh_2_ and Zn(C_6_F_5_)_2_] which effectively regenerates the more nucleophilic diarylzinc reagent.^10,11^ In the case of 5a, the RZn(C_6_F_5_) and “PhZn(C_6_F_5_)” components are retained together, at least in the solid-state under the conditions employed for crystallisation. In solution however, it is proposed that “PhZn(C_6_F_5_)” dissociates from 5 which gives compound 6 as the ultimate product of the reaction, which affords the corresponding amine 2 upon acidic quench. Multinuclear NMR spectroscopy studies show that the dissolution of crystalline 5a in THF-d_8_ affords characteristic signals for both ZnPh_2_ and Zn(C_6_F_5_)_2_ supporting the dissociation of “PhZn(C_6_F_5_)” and subsequent regeneration of the two homoleptic zinc reagents. The remaining signals observed in the ^1^H and ^19^F NMR spectra are attributed to 5a and 6; the latter species can be rationally prepared *in situ* by deprotonative zincation of 2a with Zn(C_6_F_5_)_2_ and further supports the proposed mechanism outlined in Scheme 3 (see ESI† for full spectroscopic details).

**Scheme 3:**
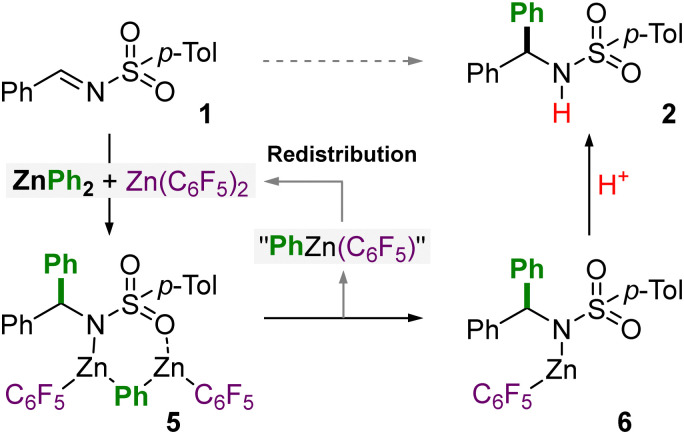
Proposed mechanism for the atom-efficient nucleophilic addition of ZnPh_2_ to *N*-tosylimines facilitated by Zn(C_6_F_5_)_2_.

Zn(C_6_F_5_)_2_ therefore plays two distinct roles in the reaction: (i) it acts as a powerful Lewis acid (see 4a, Fig. 1) to increase the electrophilicity of the imine and facilitate 1,2-addition of the nucleophilic diarylzinc reagent; and (ii) it enables the effective regeneration of the diarylzinc reagent to allow a limiting 50 mol% to be employed in the reaction. Alternatively, it could be proposed that Zn(C_6_F_5_)_2_ simply acts as an innocent Lewis acid to allow RZnPh (*e.g.*3a) to undergo a second nucleophilic addition to *N*-tosylimine 1, however this mechanistic proposal was ruled out since this would be expected to be catalytic in Zn(C_6_F_5_)_2_ (as well as other Lewis acids) and no evidence to support this pathway could be observed by NMR spectroscopy.

In conclusion, we have demonstrated how Zn/Zn cooperativity can be exploited to facilitate the atom-efficient arylation of *N*-tosylimines, activating ZnAr_2_ reagents towards the transfer of both of its Ar groups under mild conditions. Mechanistic studies through the isolation and structural characterisation of key intermediates reveals how Zn(C_6_F_5_)_2_ acts as a Lewis acid to activate the imine substrate whilst also enabling the regeneration of ZnAr_2_.

We thank the SNSF (188573) and the Universität Bern for their generous sponsorship of this research.

## Conflicts of interest

There are no conflicts to declare.

## Supplementary Material

CC-059-D3CC01490H-s001

CC-059-D3CC01490H-s002
