# Supplementary material for: Atom-efficient arylation of N-tosylimines mediated by cooperative ZnAr2/Zn(C6F5)2 combinations
Source: Chem Commun (Camb). 2023 May 15;59(49):7583–6. doi: 10.1039/d3cc01490h (PMC10270240; doi:10.1039/d3cc01490h)
Supplement: CC-059-D3CC01490H-s001 [file CC-059-D3CC01490H-s001.pdf]

Electronic Supplementary Material (ESI) for ChemComm.
This journal is © The Royal Society of Chemistry 2023

Electronic Supplementary Information

Atom-efficient arylation of *N*-tosylimines mediated by cooperative $ZnAr_2/Zn(C_6F_5)_2$ combinations

A. M. Borys, T. Kunzmann, J. M. Gil-Negrete, E. Hevia*

Table of Contents

Experimental 4
General Considerations 4
Synthesis of *N*-tosylimines (**1a–p**) 4
N-Benzylidene-4-methylbenzenesulfonamide (**1a**) 4
N-(4-Methylbenzylidene)-4-methylbenzenesulfonamide (**1b**) 5
N-(4-Methoxybenzylidene)-4-methylbenzenesulfonamide (**1c**) 5
N-(4-Chlorobenzylidene)-4-methylbenzenesulfonamide (**1d**) 5
N-(2-Methoxybenzylidene)-4-methylbenzenesulfonamide (**1g**) 6
N-(4-Fluorobenzylidene)-4-methylbenzenesulfonamide (**1k**) 6
N-(4-*N',N'*-Dimethylaminobenzylidene)-4-methylbenzenesulfonamide (**1l**) 6
N-(4-Nitrobenzylidene)-4-methylbenzenesulfonamide (**1m**) 7
N-(4-cyanobenzylidene)-4-methylbenzenesulfonamide (**1n**) 7
N-(2-Chlorobenzylidene)-4-methylbenzenesulfonamide (**1o**) 7
N-(3-Pyridylmethylene)-4-methylbenzenesulfonamide (**1p**) 8
Synthesis of *N*-benzhydryl-4-methylbenzenesulfonamides (**2a–p**) 8
N-Benzhydryl-4-methylbenzenesulfonamide (**2a**) 8
4-Methyl-*N*-(phenyl(*p*-tolyl)methyl)benzenesulfonamide (**2b**) 9
N-(4-Methoxyphenyl)(phenyl)methyl-4-methylbenzenesulfonamide (**2c**) 9
N-(4-Chlorophenyl)(phenyl)methyl-4-methylbenzenesulfonamide (**2d**) 9
N-(4-Trifluoromethylphenyl)(phenyl)methyl-4-methylbenzenesulfonamide (**2e**) 10
N-(2-Methylphenyl)(phenyl)methyl-4-methylbenzenesulfonamide (**2f**) 10
N-(2-Methoxyphenyl)(phenyl)methyl-4-methylbenzenesulfonamide (**2g**) 11
N-(2,6-Dimethylphenyl)(phenyl)methyl-4-methylbenzenesulfonamide (**2h**) 11
N-(1-Naphthyl)(phenyl)methyl-4-methylbenzenesulfonamide (**2i**) 11
N-(2-Thienyl)(phenyl)methyl-4-methylbenzenesulfonamide (**2j**) 12
N-(4-Fluorophenyl)(phenyl)methyl-4-methylbenzenesulfonamide (**2k**) 12
N-(4-*N',N'*-dimethylaminophenyl)(phenyl)methyl-4-methylbenzenesulfonamide (**2l**) 13
N-(4-Nitrophenyl)(phenyl)methyl-4-methylbenzenesulfonamide (**2m**) 13
N-(4-Cyanophenyl)(phenyl)methyl-4-methylbenzenesulfonamide (**2n**) 13
N-(2-Chlorophenyl)(phenyl)methyl-4-methylbenzenesulfonamide (**2o**) 14
N-(3-Pyridyl)(phenyl)methyl-4-methylbenzenesulfonamide (**2p**) 14
Synthesis of Zinc Complexes (**3a–5a**) 15
3a 15
3a.TMEDA 15
4a 16
5a 16
Reaction Optimisation 17
Comparison of Conditions 17

Spectroscopic Studies 18
X-ray Crystallography 22
Molecular Structure of **3a** 25
Molecular Structure of **3a.TMEDA** 25
Molecular Structure of **4a** 26
Molecular Structure of **5a** 26
NMR Spectra of Reported Compounds 27
References 59

Experimental

General Considerations

All manipulations were carried out under an inert atmosphere of argon using standard Schlenk line[1,2] or glove-box techniques (MBraun UNILab Pro ECO, <0.5 ppm H_2O and O_2). THF was dried and distilled from Na/benzophenone and stored over 4 Å molecular sieves. Hexane, Et_2O, toluene and benzene were dried using a MBraun MBSPS 5 and stored over 4 Å molecular sieves. THF-d_8, toluene-d_8 and C_6D_6 were dried and distilled over $NaK_{2.8}$ and stored over 4 Å molecular sieves in a glove-box prior to use. $Zn(C_6F_5)_2$ was purchased from Sigma Aldrich or ChemCruz and used as received unless other specified. TMEDA was dried and distilled over CaH_2 and stored over 4 Å molecular sieves. All diarylzinc reagents were prepared according to established literature procedures[3,4] and sublimed or thoroughly dried *in vacuo* prior to use to remove residual Et_2O. All other reagents were used as supplied unless otherwise stated.

NMR spectra were recorded on a Bruker Avance III HD 300 MHz spectrometer at 300 K unless otherwise specified. ^1H NMR spectra were referenced internally to the corresponding residual *protio* solvent peaks. CHN elemental microanalyses were performed on a Flash 2000 Organic Elemental Analyser (Thermo Scientific). High resolution mass spectra were recorded on a Thermo Scientific LTQ Orbitrap XL spectrometer (HRMS ESI, nano-electrospray) in positive ionisation mode (samples were diffused in a stream of MeCN).

Synthesis of *N*-tosylimines (1a–p)

N-tosylimines (**1a–p**) were prepared following the general unoptimised procedure.[5] To a 0.2 M solution of the corresponding aldehyde (1.05 equivalents) in anhydrous benzene containing activated 4 Å molecular sieves, *p*-toluenesulfonamide (1 equivalents) and $BF_3 \cdot OEt_2$ (1 equivalents) was added at room temperature. The reaction was heated at 80 °C overnight (14–18 hours) then cooled to room temperature and filtered through celite. All volatiles were removed *in vacuo* affording the corresponding imines, often without the need for further purification. Imine **1p** was prepared using catalytic piperidine (20 mol%) in DCM according to literature procedures.[6]

N-Benzylidene-4-methylbenzenesulfonamide (1a)

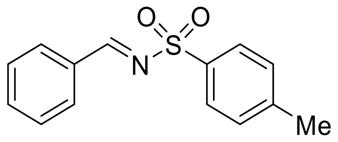

Colourless solid (98%).

^{1}H NMR (300.1 MHz, 298 K, $CDCl_3$): δ 9.03 (s, 1H, C*H*=N), 7.97-7.85 (m, 4H, Ar-C*H*), 7.61 (t, *J* = 7.4 Hz, 1H, Ar-C*H*), 7.48 (t, *J* = 7.5 Hz, 2H, Ar-C*H*), 7.35 (d, *J* = 8.0 Hz, 2H, Ar-C*H*), 2.44 (s, 3H, CH_3).

^{13}C{^{1}H} NMR (75.5 MHz, 298 K, $CDCl_3$): δ 170.3, 144.8, 135.2, 135.1, 132.5, 131.5, 129.9, 129.3, 128.2, 21.8.

Analytical data in accordance with the literature.[5]

N-(4-Methylbenzylidene)-4-methylbenzenesulfonamide (1b)

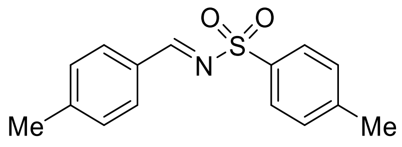

Colourless solid (92%).

^{1}H NMR (300.1 MHz, 298 K, $CDCl_3$): δ 8.98 (s, 1H, C*H*=N), 7.88 (d, *J* = 8.3 Hz, 2H, Ar-C*H*), 7.81 (d, *J* = 8.0 Hz, 2H, Ar-C*H*), 7.37-7.24 (m, 4H, Ar-C*H*), 2.43 (s, 6H, CH_3).

^{13}C{^{1}H} NMR (75.5 MHz, 298 K, $CDCl_3$): δ 170.1, 146.5, 144.6, 135.5, 133.2, 130.1, 130.0, 129.9, 128.2, 22.1, 21.8.

Analytical data in accordance with the literature.[7]

N-(4-Methoxybenzylidene)-4-methylbenzenesulfonamide (1c)

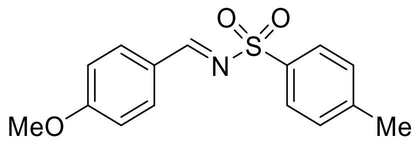

Colourless solid (96%).

^{1}H NMR (300.1 MHz, 298 K, $CDCl_3$): δ 8.94 (s, 1H, C*H*=N), 7.95-7-84 (m, 4H, Ar-C*H*), 8.1 (d, *J* = 8.1 Hz, 2H, Ar-C*H*), 6.96 (d, *J* = 8.9 Hz, 2 Hz, Ar-C*H*), 3.88 (s, 3H, OCH_3), 2.43 (s, 3H, CH_3).

^{13}C{^{1}H} NMR (75.5 MHz, 298 K, $CDCl_3$): δ 169.3, 165.4, 144.4, 135.9, 133.9, 129.9, 128.0, 125.7, 114.8, 55.8, 21.8.

Analytical data in accordance with the literature.[5]

N-(4-Chlorobenzylidene)-4-methylbenzenesulfonamide (1d)

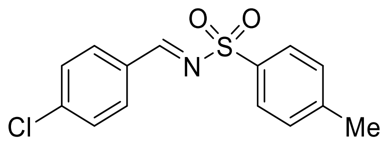

Colourless solid (88%).

^{1}H NMR (300.1 MHz, 298 K, $CDCl_3$): δ 8.99 (s, 1H, C*H*=N), 7.87 (dd, *J* = 8.4, 6.3 Hz, 4H, Ar-C*H*), 7.47 (d, *J* = 8.5 Hz, 2H, Ar-C*H*), 7.35 (d, *J* = 8.0 Hz, 2H, Ar-C*H*), 2.44 (s, 3H, CH_3).

^{13}C{^{1}H} NMR (75.5 MHz, 298 K, $CDCl_3$): δ 168.8, 144.9, 141.6, 135.1, 132.5, 130.2, 130.0, 129.8 (Ar-*C*H), 128.3, 21.8.

Analytical data in accordance with the literature.[7]

***N*-(2-Methoxybenzylidene)-4-methylbenzenesulfonamide (1g)**

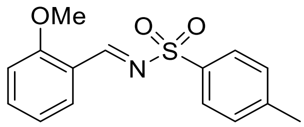

Colourless solid (95%).

^{1}H NMR (300.1 MHz, 298 K, $CDCl_3$): δ 9.45 (s, 1H, C*H*=N), 7.95 (dd, *J* = 7.8, 1.8 Hz, 1H, Ar-C*H*), 7.78 (d, *J* = 8.3 Hz, 2H, Ar-C*H*), 7.45 (ddd, *J* = 8.4, 7.3, 1.8 Hz, 1H, Ar-C*H*), 7.22 (d, *J* = 8.0 Hz, 2H, Ar-C*H*), 6.92-6.81 (m, 2H, Ar-C*H*), 3.81 (s, 3H, OCH_3), 2.33 (s, 3H, CH_3).

^{13}C{^{1}H} NMR (75.5 MHz, 298 K, $CDCl_3$): δ 166.5, 161.8, 144.4, 137.0, 135.8, 129.8, 129.5, 128.5, 128.1, 121.0, 111.6, 55.9, 21.8.

Analytical data in accordance with the literature.[8]

***N*-(4-Fluorobenzylidene)-4-methylbenzenesulfonamide (1k)**

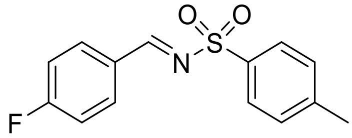

Colourless solid (79%)

^{1}H NMR (300.1 MHz, 298 K, $CDCl_3$): δ 9.00 (s, 1H, C*H*=N), 8.0-7.92 (m, 2H, Ar-C*H*), 7.88 (d, *J* = 8.3 Hz, 2H, Ar-C*H*), 7.35 (d, *J* = 7.3 Hz, 2H, Ar-C*H*), 7.18 (t, *J* = 7.2 Hz, 2H, Ar-C*H*), 2.44 (s, 3H, CH_3).

^{13}C{^{1}H} NMR (75.5 MHz, 298 K, $CDCl_3$): δ 168.7, 166, 144.8, 135.2, 133.9 (d, $J_{C\text{-}F}$ = 9.4 Hz), 130.0, 128.3, 128.0, 116.8 (d, $J_{C\text{-}F}$ = 22.0 Hz), 21.8.

^{19}F{^{1}H} NMR 282.4 MHz, 298 K, $CDCl_3$): δ -101.1 (s).

Analytical data in accordance with the literature.[7]

***N*-(4-*N’,N’*-Dimethylaminobenzylidene)-4-methylbenzenesulfonamide (1l)**

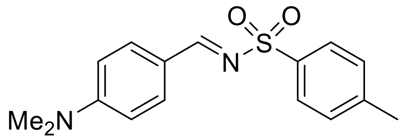

Bright yellow solid (25%).

^{1}H NMR (300.1 MHz, 298 K, $CDCl_3$): δ 8.81 (s, 1H, C*H*=N), 7.85 (d, *J* = 8.3 Hz, 2H, Ar-C*H*), 7.77 (d, *J* = 8.9 Hz, 2H, Ar-C*H*), 7.30 (d, *J* = 8.3 Hz, 2H, Ar-C*H*), 6.70 (d, *J* = 8.9 Hz, 2H, Ar-C*H*), 3.10 (s, 6H, NMe_2), 2.41 (s, 3H, CH_3).

^{13}C{^{1}H} NMR (75.5 MHz, 298 K, $CDCl_3$): δ 168.8, 154.6, 143.6, 136.7, 133.9, 129.5, 127.5, 120.2, 111.6, 40.3, 21.5.

Analytical data in accordance with the literature.[7]

N-(4-Nitrobenzylidene)-4-methylbenzenesulfonamide (1m)

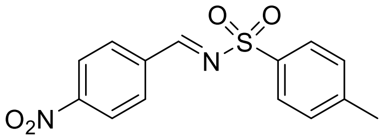

Pale tan solid (5%). Purified by recrystallisation from toluene at -30 °C.

^{1}H NMR (300.1 MHz, 298 K, $CDCl_3$): δ 9.10 (s, 1H, C*H*=N), 8.28 (d, *J* = 8.8 Hz, 2H, Ar-C*H*), 8.08 (d, *J* = 8.8 Hz, 2H, Ar-C*H*), 7.85 (d, *J* = 8.2 Hz, 2H, Ar-C*H*), 7.34 (d, *J* = 8.07 Hz, 2H, Ar-C*H*), 2.41 (s, 3H, CH_3).

^{13}C{^{1}H} NMR (75.5 MHz, 298 K, $CDCl_3$): δ 167.2, 150.6, 144.9, 137.0, 133.6, 131.5, 130.1, 129.6, 128.9, 127.8, 125.7, 123.7, 21.2.

Analytical data in accordance with the literature.[7]

N-(4-cyanobenzylidene)-4-methylbenzenesulfonamide (1n)

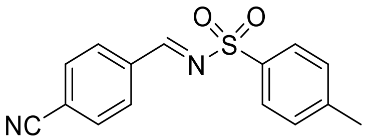

Colourless solid (9%). Purified by recrystallisation from toluene at -30 °C.

^{1}H NMR (300.1 MHz, 298 K, $CDCl_3$): δ 9.06 (s, 1H, C*H*=N), 8.04 (d, *J* = 8.4 Hz, 2H, Ar-C*H*), 7.90 (d, *J* = 8.2 Hz, 2H, Ar-C*H*), 7.78 (d, *J* = 8.4 Hz, 2H, Ar-C*H*), 7.37 (d, *J* = 8.2 Hz, 2H, Ar-C*H*), 2.45 (s, 3H, CH_3).

^{13}C{^{1}H} NMR (75.5 MHz, 298 K, $CDCl_3$): δ 167.8, 145.2, 135.9, 134.2, 132.7, 131.2, 129.9, 129.6, 128.3, 126.4, 117.6, 21.7.

Analytical data in accordance with the literature.[7]

N-(2-Chlorobenzylidene)-4-methylbenzenesulfonamide (1o)

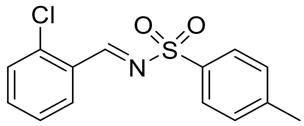

Colourless solid (94%).

^{1}H NMR (300.1 MHz, 298 K, $CDCl_3$): δ 9.50 (s, 1H, C*H*=N), 8.15 (d, *J* = 7.8 Hz, 1H, Ar-C*H*), 7.90 (d, *J* = 8.3 Hz, 2H, Ar-C*H*), 7.56-7.44 (m, 2H, Ar-C*H*), 7.40-7.32 (m, 3H, Ar-C*H*), 2.45 (s, 3H, CH_3).

^{13}C{^{1}H} NMR (75.5 MHz, 298 K, $CDCl_3$): δ 166.9, 145.0, 139.1, 135.8, 134.8, 130.6, 130.3, 130.0, 129.9, 128.4, 127.5, 21.8.

Analytical data in accordance with the literature.[9]

N-(3-Pyridylmethylene)-4-methylbenzenesulfonamide (1p)

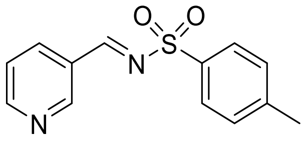

Pale yellow solid (37%). Purified by recrystallisation from hot EtOAc.

^{1}H NMR (300.1 MHz, 298 K, $CDCl_3$): δ 9.08 (s, 1H, C*H*=N), 9.04 (d, *J* = 1.7 Hz, 1H, Ar-C*H*), 8.80 (dd, *J* = 1.7, 4.9 Hz, 1H, Ar-C*H*), 8.27 (dt, *J* = 8.0, 1.9 Hz, 1H, Ar-C*H*), 7.89 (d, *J* = 8.3 Hz, 2H, Ar-C*H*), 7.43 (d, *J* = 4.9, 8.0 Hz, 1H, Ar-C*H*), 7.36 (d, *J* = 8.0 Hz, 2H, Ar-C*H*), 2.44 (s, 3H, C*H*$_3$).

^{13}C{^{1}H} NMR (75.5 MHz, 298 K, $CDCl_3$): δ 167.5, 155.0, 152.9, 145.0, 136.9, 134.4, 129.9, 128.2, 124.0, 21.6.

Analytical data in accordance with the literature.[6]

Synthesis of *N*-benzhydryl-4-methylbenzenesulfonamides (2a–p)

N-Benzhydryl-4-methylbenzenesulfonamines (**2a–p**) were prepared following the general procedure. *N*-tosylimine (**1a–p**) (0.25 mmol), $Zn(C_6F_5)_2$ (50 mg, 0.125 mmol, 0.5 equivalents) and $ZnAr_2$ (0.125 mmol, 0.5 equivalents) were dissolved in toluene (2.5 mL) and stirred for 2 hours at room temperature. The reaction was quenched with MeOH (1 mL) and extracted into Et_2O (3 × 15 mL). The organic phase was washed with H_2O (2 × 15 mL) then dried over Na_2SO_4, filtered, and evaporated to dryness. The crude products were purified by silica gel chromatography using a CombiFlash®Rf system (Teledyne ISCO), with RediSep® Silver Normal-phase Silica Flash Columns as stationary phase and a hexane/EtOAc gradient elution (up to 20% EtOAc) as mobile phase.

N-Benzhydryl-4-methylbenzenesulfonamide (2a)

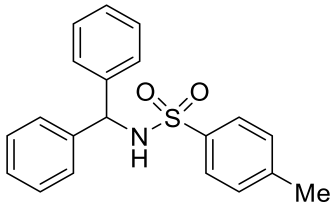

Colourless solid (71%).

^{1}H NMR (300.1 MHz, 298 K, $CDCl_3$): δ 7.57 (d, *J* = 8.3 Hz, 2H, Ar-C*H*), 7.24–7.17 (m, 6H, Ar-C*H*), 7.16-7.09 (m, 6H, Ar-C*H*), 5.59 (d, *J* = 7.3 Hz, 1H, C*H*), 5.44 (d, *J* = 7.3 Hz, 1H, N*H*), 2.38 (s, 3H, C*H*$_3$).

^{13}C{^{1}H} NMR (75.5 MHz, 298 K, $CDCl_3$): δ 143.1, 140.5, 137.3, 129.3, 128.5, 127.5, 127.3, 127.1, 61.3, 21.4.

Analytical data in accordance with the literature.[10]

4-Methyl-*N*-(phenyl(*p*-tolyl)methyl)benzenesulfonamide (2b)

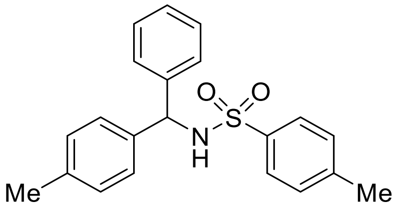

Colourless solid (75% from **1a** and Zn(4-Me-C_6H_4)$_2$; 48% from **1b** and $ZnPh_2$).

^{1}H NMR (300.1 MHz, 298 K, $CDCl_3$): δ 7.57 (d, *J* = 8.3 Hz, 2H, Ar-C*H*), 7.24–7.17 (m, 3H, Ar-C*H*), 7.17–7.09 (m, 4H, Ar-C*H*), 7.05–6.96 (m, 4H, Ar-C*H*), 5.54 (d, *J* = 7.2 Hz, 1H, C*H*), 5.37 (d, *J* = 7.2 Hz, 1H, N*H*), 2.39 (s, 3H, CH_3), 2.29 (s, 3H, CH_3).

^{13}C{^{1}H} NMR (75.5 MHz, 298 K, $CDCl_3$): δ 143.0, 140.7, 137.6, 137.4, 137.2, 129.3, 129.1, 128.4, 127.4, 127.3, 127.2, 127.1, 61.1, 21.4, 21.0.

Analytical data in accordance with the literature.[11]

***N*-(4-Methoxyphenyl)(phenyl)methyl-4-methylbenzenesulfonamide (2c)**

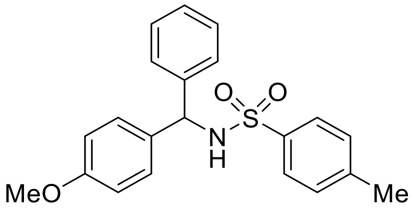

Colourless solid (61% from **1a** and Zn(4-OMe-C_6H_4)$_2$; 34% from **1c** and $ZnPh_2$).

^{1}H NMR (300.1 MHz, 298 K, $CDCl_3$): δ 7.57 (d, *J* = 8.3 Hz, 2H, Ar-C*H*), 7.23–7.17 (m, 4H, Ar-C*H*), 7.16–7.09 (m, 4H, Ar-C*H*), 7.01 (d, *J* = 8.5 Hz, 2H, Ar-C*H*), 6.73 (d, *J* = 8.8 Hz, 2H, Ar-C*H*), 5.54 (d, *J* = 7.3 Hz, 1H, C*H*), 5.39 (d, *J* = 7.3 Hz, 1H, N*H*), 3.75 (s, 3H, OCH_3), 2.38 (s, 3H, CH_3).

^{13}C{^{1}H} NMR (75.5 MHz, 298 K, $CDCl_3$): δ 158.9, 143.0, 140.7, 137.4, 132.7, 129.3, 128.6, 128.4, 127.4, 127.1, 113.8, 60.8, 55.2, 21.4.

Analytical data in accordance with the literature.[11]

***N*-(4-Chlorophenyl)(phenyl)methyl-4-methylbenzenesulfonamide (2d)**

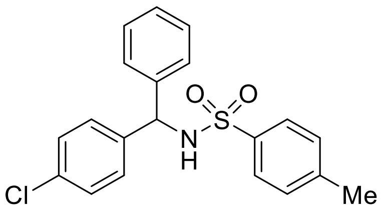

Colourless oil (55% from **1a** and Zn(4-Cl-C_6H_4)$_2$; 70% from **1d** and $ZnPh_2$).

^{1}H NMR (300.1 MHz, 298 K, $CDCl_3$): δ 7.55 (d, *J* = 6.2 Hz, 2H, Ar-C*H*), 7.25-7.12 (m, 7H, Ar-C*H*), 7.10-7.00 (m, 4H, Ar-C*H*), 5.54 (d, *J* = 5.3 Hz, 1H, C*H*), 5.08 (d, *J* = 5.3 Hz, 1H, N*H*), 2.39 (s, CH_3).

$^{13}C\{^1H\}$ NMR (75.5 MHz, 298 K, $CDCl_3$): δ 143.6 (quaternary-*C*), 140.2 (quaternary-*C*), 139.1 (quaternary-*C*), 137.3 (quaternary-*C*), 133.7 (quaternary-*C*), 129.6 (Ar-*C*H), 128.93 (Ar-*C*H), 128.90 (Ar-*C*H), 128.8 (Ar-*C*H), 128.1 (Ar-*C*H), 127.43 (Ar-*C*H), 127.35 (Ar-*C*H), 60.9 (*C*H), 21.6 (*C*H_3).

Analytical data in accordance with the literature.[11]

_N_-(4-Trifluoromethylphenyl)(phenyl)methyl-4-methylbenzenesulfonamide (2e)

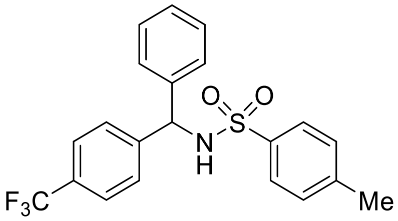

Colourless solid (38% from **1a** and Zn(4-CF_3-C_6H_4)$_2$).

^{1}H NMR (300.1 MHz, 298 K, $CDCl_3$): δ 7.43 (d, *J* = 8.2 Hz, 2H, Ar-C*H*), 7.31 (d, *J* = 8.2 Hz, 2H, Ar-C*H*), 7.16 (d, *J* = 8.3 Hz, 2H, Ar-C*H*), 7.10 (m, 3H, Ar-C*H*), 6.98 (m, 4H, Ar-C*H*), 5.89 (d, *J* = 7.9 Hz, 1H, C*H*), 5.52 (d, *J* = 7.9 Hz, 1H, N*H*), 2.25 (s, 3H, CH_3).

$^{13}C\{^1H\}$ NMR (75.5 MHz, 298 K, $CDCl_3$): δ 144.4, 143.4, 139.7, 137.0, 129.7, 129.3, 128.7, 127.9, 127.7, 127.2, 127.1, 125.3 (q, $J_{C\text{-}F}$ = 3.9 Hz), 123.9 (q, $J_{C\text{-}F}$ = 272.0 Hz), 61.0, 21.3.

$^{19}F\{^1H\}$ NMR (282.4 MHz, 298 K, $CDCl_3$): δ -62.6 (s).

Analytical data in accordance with the literature.[12]

_N_-(2-Methylphenyl)(phenyl)methyl-4-methylbenzenesulfonamide (2f)

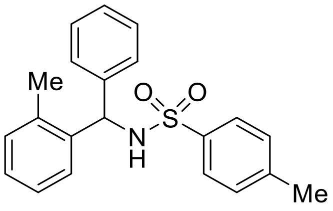

Colourless solid (64% from **1a** and Zn(2-Me-C_6H_4)$_2$).

^{1}H NMR (300.1 MHz, 298 K, $CDCl_3$): δ 7.56 (d, *J* = 8.3 Hz, 2H, Ar-C*H*), 7.20 (m, 3H, Ar-C*H*), 7.16–7.03 (m, 8H, Ar-C*H*), 5.82 (d, *J* = 7.2 Hz, 1H, C*H*), 5.35 (d, *J* = 7.2 Hz, 1H, N*H*), 2.37 (s, 3H, CH_3), 2.18 (s, 3H, CH_3).

$^{13}C\{^1H\}$ NMR (75.5 MHz, 298 K, $CDCl_3$): δ 156.2, 142.7, 140.6, 137.4, 129.4, 128.9, 128.8, 128.0, 127.6, 127.0, 126.9, 126.7, 120.5, 110.9, 58.8, 55.1, 21.3.

Analytical data in accordance with the literature.[12]

N-(2-Methoxyphenyl)(phenyl)methyl-4-methylbenzenesulfonamide (2g)

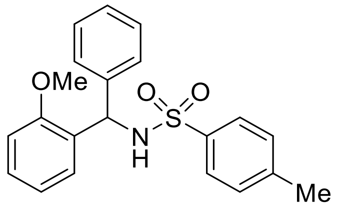

Colourless solid (59% from **1a** and Zn(2-OMe-C_6H_4)$_2$; 77% from **1g** and $ZnPh_2$).

^{1}H NMR (300.1 MHz, 298 K, $CDCl_3$): δ 7.55 (d, *J* = 8.2 Hz, 2H, Ar-C*H*), 7.25–7.12 (m, 6H, Ar-C*H*), 7.04 (m 3H, Ar-C*H*), 6.78 (t, *J* = 7.4 Hz, 1H, Ar-C*H*), 6.67 (d, *J* = 8.2 Hz, 1H, Ar-C*H*), 6.01 (d, *J* = 9.3 Hz, 1H, C*H*), 5.70 (d, *J* = 9.3 Hz, 1H, N*H*), 3.59 (s, 3H, OC*H*$_3$), 2.33 (s, 3H C*H*$_3$).

^{13}C{^{1}H} NMR (75.5 MHz, 298 K, $CDCl_3$): δ 156.2, 142.6, 140.6, 137.4, 129.4, 128.9, 128.8, 128.0, 127.6, 127.0, 126.9, 126.7, 120.5, 110.9, 58.8, 55.1, 21.3.

Analytical data in accordance with the literature.[11]

N-(2,6-Dimethylphenyl)(phenyl)methyl-4-methylbenzenesulfonamide (2h)

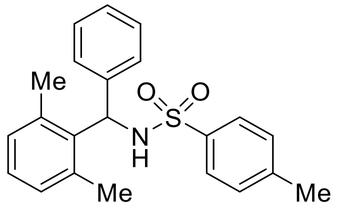

Colourless solid (30% yield from **1a** and Zn(2,6-Me_2-C_6H_3)$_2$).

^{1}H NMR (300.1 MHz, 298 K, $CDCl_3$): δ 7.76 (d, *J* = 8.3 Hz, Ar-C*H*), 7.50–7.39 (m, 5H, Ar-C*H*), 7.30 (d, *J* = 8.0 Hz, 2H, Ar-C*H*), 7.23 (d, *J* = 7.5 Hz, 1H, Ar-C*H*), 7.09 (d, *J* = 7.5 Hz, 2H, Ar-*C*H), 6.39 (d, *J* = 8.7 Hz, 1H, C*H*), 5.75 (d, *J* = 8.7 Hz, 1H, N*H*), 2.57 (s, 3H, *CH$_3$*), 2.29 (s, 6H, C*H$_3$*).

^{13}C{^{1}H} NMR (75.5 MHz, 298 K, $CDCl_3$): δ 142.9, 139.8, 137.4, 136.4, 136.3, 129.1, 128.4, 127.6, 127.1, 126.6, 126.1, 55.9, 21.4, 20.6.

HRMS: *m/z* calculated for $C_{22}H_{27}O_2N_2S$ [M-NH_4]+ 383.1798; found 383.1788.

N-(1-Naphthyl)(phenyl)methyl-4-methylbenzenesulfonamide (2i)

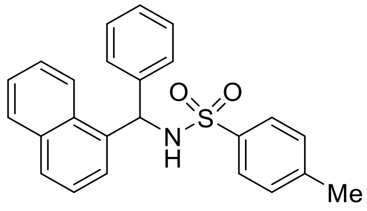

Colourless solid (68% yield from **1a** and Zn(1-Naphthyl)$_2$).

^{1}H NMR (300.1 MHz, 298 K, $CDCl_3$): δ 7.78 (m, 2H, Ar-C*H*), 7.68 (dd, *J* = 1.7, 7.4 Hz, 1H, Ar-C*H*), 7.46 (d, *J* = 8.3 Hz, 2H, Ar-CH), 7.38 (m, 2H, Ar-C*H*), 7.22 (m, 2H, Ar-C*H*), 7.18–7.09 (m, 5H, Ar-C*H*), 7.00 (d, *J* = 8.1 Hz, 2H, Ar-C*H*), 6.29 (d, *J* = 7.2 Hz, 1H, C*H*), 5.25 (d, *J* = 7.2 Hz, 1H, N*H*), 2.30 (s, CH_3).

^{13}C{^{1}H} NMR (75.5 MHz, 298 K, $CDCl_3$): δ 143.1, 140.2, 137.2, 135.5, 133.9, 130.4, 129.2, 128.8, 128.5, 127.6, 127.5, 127.1, 126.5, 126.1, 125.7, 125.0, 123.4, 58.5, 21.4.

Analytical data in accordance with the literature.[11]

_N_-(2-Thienyl)(phenyl)methyl-4-methylbenzenesulfonamide (2j)

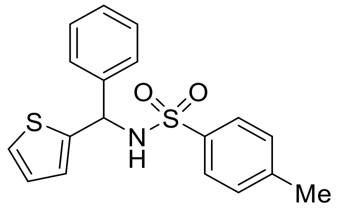

Colourless solid (80% yield from **1a** and Zn(2-Thienyl)$_2$).

^{1}H NMR (300.1 MHz, 298 K, $CDCl_3$): δ 7.60 (d, *J* = 8.3 Hz, 2H, Ar-C*H*), 7.22 (m, 5H, Ar-C*H*), 7.15 (m, 3H, Ar-C*H*), 6.82 (dd, *J* = 3.6, 5.1 Hz, 1H, Ar-C*H*), 6.68 (m, 1H, Ar-C*H*), 5.81 (d, *J* = 7.8 Hz, 1H, C*H*), 5.68 (d, *J* = 7.8 Hz, 1H, N*H*), 2.38 (s, 3H, CH_3).

^{13}C{^{1}H} NMR (75.5 MHz, 298 K, $CDCl_3$): δ 144.8, 143.1, 140.0, 137.2, 129.3, 128.4, 127.8, 127.1, 127.0, 126.6, 126.0, 125.6, 57.4, 21.4.

Analytical data in accordance with the literature.[11]

_N_-(4-Fluorophenyl)(phenyl)methyl-4-methylbenzenesulfonamide (2k)

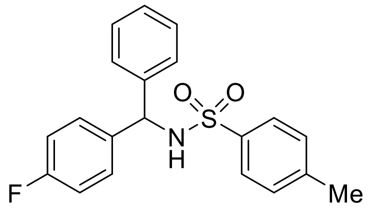

Colourless solid (68% from **1k** and $ZnPh_2$).

^{1}H NMR (300.1 MHz, 298 K, $CDCl_3$): δ 7.55 (d, *J* = 6.2 Hz, 2H, Ar-C*H*), 7.24-7.18 (m, 3H, Ar-C*H*), 7.14 (d, *J* = 6.0 Hz, 2H, Ar-C*H*), 7.11-7.04 (m, 4H, Ar-C*H*), 6.89 (t, *J* = 6.5 Hz, 2H, Ar-C*H*), 5.55 (d, *J* = 5.4 Hz, 1H, C*H*), 5.25 (d, *J* = 5.4 Hz, 1H, N*H*), 2.38 (s, 3H, OCH_3).

^{13}C{^{1}H} NMR (75.5 MHz, 298 K, $CDCl_3$): δ 162.2 (d, J_{C-F} = 246.6 Hz), 134.4, 140.4, 137.4, 136.4 (d, J_{C-F} = 2.4 Hz), 129.5, 129.2 (d, J_{C-F} = 6.1 Hz), 128.8, 127.9, 127.4, 127.3, 115.5 (d, J_{C-F} = 16.2 Hz), 60.8, 21.6.

^{19}F{^{1}H} NMR (282.4 MHz, 298 K, $CDCl_3$): δ -114.8 (s).

Analytical data in accordance with the literature.[11]

N-(4-N',N'-dimethylaminophenyl)(phenyl)methyl-4-methylbenzenesulfonamide (2l)

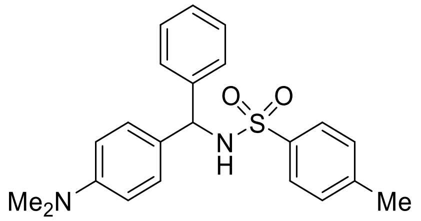

Pale yellow solid (9% yield from **1l** and $ZnPh_2$).

^{1}H NMR (300.1 MHz, 298 K, $CDCl_3$): δ 7.57 (d, *J* = 8.3 Hz, 2H, Ar-C*H*), 7.24–7.11 (m, 7H, Ar-C*H*), 6.93 (d, *J* = 8.4 Hz, 2H, Ar-CH), 6.61 (br, 2H, Ar-C*H*), 5.49 (d, *J* = 6.7 Hz, 1H, C*H*), 4.99 (d, *J* = 6.7 Hz, 1H, N*H*), 2.91 (s, 6H, N*Me₂*), 2.39 (s, 3H, C*H₃*).

^{13}C{^{1}H} NMR (75.5 MHz, 298 K, $CDCl_3$): δ 143.0, 140.9, 137.5, 129.3, 128.4, 127.3, 127.2, 112.7, 60.9, 40.8, 21.5.

Analytical data in accordance with the literature.[13]

N-(4-Nitrophenyl)(phenyl)methyl-4-methylbenzenesulfonamide (2m)

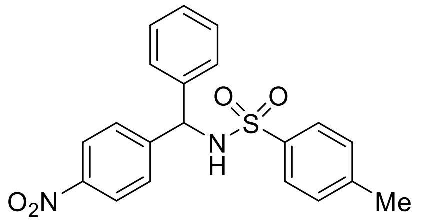

Colourless solid (67% yield from **1m** and $ZnPh_2$).

^{1}H NMR (300.1 MHz, 298 K, $CDCl_3$): δ 8.03 (d, *J* = 8.7 Hz, 2H, Ar-C*H*), 7.57 (d, *J* = 8.2 Hz, 2H, Ar-C*H*), 7.37 (d, *J* = 8.7 Hz, 2H, Ar-C*H*), 7.22 (m, 3H), 7.14 (d, *J* = 8.2 Hz, 2H, Ar-C*H*), 7.02 (m, 2H, Ar-C*H*), 5.94 (d, *J* = 7.5 Hz, 1H, C*H*), 5.62 (d, *J* = 7.5 Hz, 1H, N*H*), 2.38 (s, 3H, CH_3).

^{13}C{^{1}H} NMR (75.5 MHz, 298 K, $CDCl_3$): δ 147.8, 147.0, 143.7, 139.2, 136.8, 129.5, 128.9, 128.2, 127.2, 127.1, 123.5, 60.8, 21.4.

Analytical data in accordance with the literature.[11]

N-(4-Cyanophenyl)(phenyl)methyl-4-methylbenzenesulfonamide (2n)

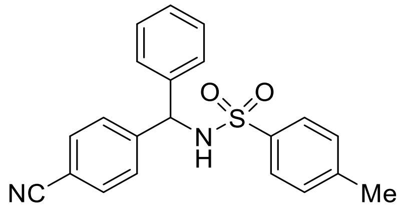

Colourless solid (61% yield from **1n** and $ZnPh_2$).

^{1}H NMR (300.1 MHz, 298 K, $CDCl_3$): δ 7.56 (d, *J* = 8.3 Hz, 2H, Ar-C*H*), 7.48 (d, *J* = 8.4 Hz, 2H, Ar-C*H*), 7.30 (d, *J* = 8.3 Hz, 2H, Ar-C*H*), 7.21 (m, 3H, Ar-C*H*), 7.14 (d, *J* = 8.0 Hz, 2H, Ar-C*H*), 7.00 (m, 2H, Ar-C*H*), 5.86 (d, *J* = 7.6 Hz, 1H, C*H*), 5.57 (d, *J* = 7.6 Hz, 1H, N*H*), 2.39 (s, 3H, CH_3).

^{13}C{^{1}H} NMR (75.5 MHz, 298 K, $CDCl_3$): δ 145.8, 143.6, 139.3, 136.9, 132.2, 129.4, 128.9, 128.1, 128.0, 127.2, 127.0, 118.5, 111.2, 60.9, 21.4.

Analytical data in accordance with the literature.[10]

_N_-(2-Chlorophenyl)(phenyl)methyl-4-methylbenzenesulfonamide (2o)

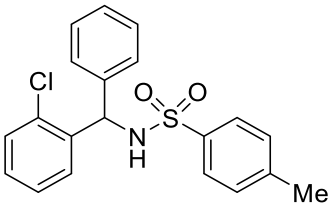

Colourless solid (62% from **1o** and $ZnPh_2$).

^{1}H NMR (300.1 MHz, 298 K, $CDCl_3$): δ 7.62 (d, *J* = 8.3 Hz, 2H, Ar-C*H*), 7.39-7.32 (m, 1H, Ar-C*H*), 7.28-7.20 (m, 4H, Ar-C*H*), 7.19-7.12 (m, 2H, Ar-C*H*), 5.94 (d, *J* = 7.4 Hz, 1H, C*H*), 5.46 (d, *J* = 7.4 Hz, 1H, N*H*), 2.38 (s, 3H, CH_3).

^{13}C{^{1}H} NMR (75.5 MHz, 298 K, $CDCl_3$): δ 143.5, 139.4, 137.7, 137.1, 132.9, 130.0, 129.5, 129.4, 128.9, 128.8, 127.9, 127.4, 127.3, 127.1, 58.7, 21.6.

Analytical data in accordance with the literature.[11]

_N_-(3-Pyridyl)(phenyl)methyl-4-methylbenzenesulfonamide (2p)

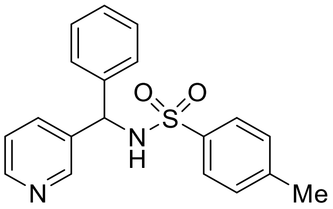

Colourless solid (83% yield from **1q** and $ZnPh_2$).

^{1}H NMR (300.1 MHz, 298 K, $CDCl_3$): δ 8.37 (d, *J* = 3.9 Hz, 1H, Ar-C*H*), 8.28 (s, 1H, Ar-C*H*), 7.54 (t, *J* = 8.2, 3H, Ar-C*H*), 7.20 (m, 3H, Ar-C*H*), 7.16–7.09 (m, 3H, Ar-C*H*), 7.04 (m, 2H, Ar-C*H*), 6.44 (d, *J* = 7.4 Hz, 1H, C*H*), 5.57 (d, *J* = 7.4 Hz, 1H, N*H*), 2.36 (s, 3H, CH_3).

^{13}C{^{1}H} NMR (75.5 MHz, 298 K, $CDCl_3$): δ 148.6, 148.4, 143.3, 139.5, 137.1, 136.4, 135.1, 129.4, 128.8, 127.3, 127.0, 123.3, 59.2 (<u>C</u>H), 21.4 (<u>C</u>H_3).

Analytical data in accordance with the literature.[6]

Synthesis of Zinc Complexes (3a–5a)

3a

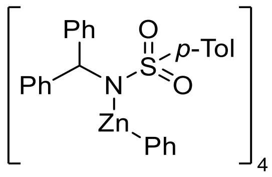

N-Benzylidene-4-methylbenzenesulfonamide **1a** (52 mg, 0.2 mmol) and $ZnPh_2$ (44 mg, 0.2 mmol) were dissolved in toluene (2 mL) and left undisturbed at room temperature for 48 hours affording colourless crystals. The supernatant was decanted, and the crystals were washed with hexane (2 × 1 mL) and dried *in vacuo*. Yield – 90 mg (94%).

^{1}H NMR (300.1 MHz, 298 K, THF-d_8): δ 7.53 (d, *J* = 8.2 Hz, 2H, Ar-C*H*), 7.41 (br, 2H, Ar-C*H*), 7.21 (d, *J* = 7.0 Hz, 4H, Ar-C*H*), 7.14–7.03 (m, 8H, Ar-C*H*), 6.99 (t, *J* = 8.1 Hz, 3H, Ar-C*H*), 5.51 (s, 1H, C*H*), 2.22 (s, 3H, CH_3).

^{13}C{^{1}H} NMR (75.5 MHz, 298 K, TH-d_8): δ 150.8, 145.7, 141.8, 141.7, 139.5, 129.5, 129.0, 128.7, 128.3, 127.8, 127.4, 126.2, 64.1, 21.4.

Elemental Analysis: Calculated for $\{C_{26}H_{23}NO_2SZn\}_4$: C, 65.21; H, 4.84; N, 2.92. Found: C, 66.25; H, 4.94; N, 2.86.

3a.TMEDA

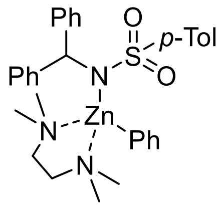

N-Benzylidene-4-methylbenzenesulfonamide **1a** (100 mg, 0.386 mmol) and $ZnPh_2$ (85 mg, 0.386 mmol) were dissolved in toluene (3 mL) and stirred at room temperature for 2 hours. TMEDA (0.06 mL, 0.40 mmol) was added causing the formation of a colourless precipitate which was redissolved by the addition of toluene (2 mL) and hexane (1 mL). Storage at -30 °C for 24 hours afforded colourless crystals that were isolated by filtration and dried *in vacuo* (155 mg, 68%).

^{1}H NMR (300.1 MHz, 298 K, C_6D_6): δ 7.89.7.82 (m, 2H, Ar-C*H*), 7.78-7.70 (m, 2H, Ar-C*H*), 7.48-7.37 (m, 7H, Ar-C*H*), 7.32 (t, *J* = 7.3 Hz, 1H, Ar-C*H*), 7.14-6.90 (m, 7H, Ar-C*H*), 6.67 (d, *J* = 8.0 Hz, 2H, Ar-C*H*), 5.99 (s, 1H, C*H*), 2.07 (s, 12H, CH_3), 1.81 (s, 3H, CH_3), 1.76 (m, 4H, CH_2).

^{13}C{^{1}H} NMR (75.5 MHz, 298 K, C_6D_6): δ 158.2, 144.5, 141.7, 140.0, 139.7, 129.4, 129.0, 128.3, 127.5, 126.5, 126.1, 125.3, 125.1, 63.4, 56.9, 47.9, 20.6.

Elemental Analysis: Calculated for $C_{32}H_{39}N_3O_2SZn$; C, 64.58, H, 6.61, N, 7.06. Found: C, 64.47; H, 6.51; N, 7.13.

4a

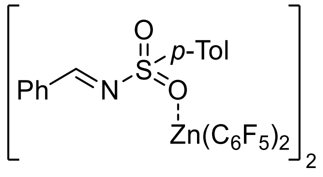

N-Benzylidene-4-methylbenzenesulfonamide **1a** (80 mg, 0.31 mmol) and sublimed $Zn(C_6F_5)_2$ (125 mg, 0.31 mmol) were combined in toluene (6 mL) and gently warmed to form a clear solution. On cooling to room temperature and standing overnight, colourless crystals of **4a** were isolated by filtration and dried *in vacuo*. Yield – 69 mg, 34%.

N.B. Due to poor solubility in non-donor solvents, $^{13}C\{^1H\}$ NMR characterisation of **4a** was not possible.

^{1}H NMR (300.1 MHz, 298 K, Tol-d_8): δ 8.91 (s, 1H, C*H*=N), 7.86 (d, *J* = 8.3 Hz, 2H, Ar-C*H*), 7.37 (d, *J* = 8.4 Hz, 2H, Ar-C*H*), 7.0-6.96 (m, 1H, Ar-C*H*), 6.85 (t, *J* = 7.52 Hz, 2H, Ar-C*H*), 6.72 (d, *J* = 8.1 Hz, 2H, Ar-C*H*), 1.87 (s, 3H, CH_3).

$^{19}F\{^1H\}$ NMR (282.4 MHz, 298 K, Tol-d_8): δ -117.0 (m), -156.8 (t), -162.0 (m).

Elemental Analysis: Calculated for $\{C_{26}H_{13}F_{10}NO_2SZn\}_2$; C, 47.40; H, 1.99; N, 2.13. Found: C, 46.93; H, 1.95; N, 2.33.

5a

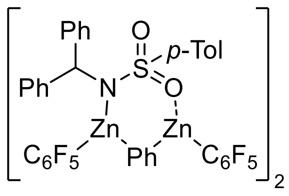

N-Benzylidene-4-methylbenzenesulfonamide **1a** (52 mg, 0.2 mmol) and $ZnPh_2$ (44 mg, 0.2 mmol) were combined in toluene (1 mL) and a solution of sublimed $Zn(C_6F_5)_2$ (80 mg, 0.2 mmol) in toluene (1 mL) was added. After stirring at room temperature for 4 hours, the colourless suspension was filtered through a short Celite plug and the filtrate was allowed to stand undisturbed for 48 hours affording a crop of large colourless crystals. The supernatant was removed, and the solids were washed with hexane (2 × 1 mL) and dried *in vacuo*. Yield – 33 mg (19%).

^{1}H NMR (300.1 MHz, 298 K, THF-d_8): δ 7.51 (t, *J* = 8.0 Hz, 2H, Ar-C*H*), 7.40 (br, 1H, Ar-C*H*), 726 (m, 1H, Ar-C*H*), 7.20 (d, *J* = 7.2 Hz, 2H), 7.14–6.96 (m, 8H, Ar-C*H*), 5.50 (br, 1H, C*H*), 2.22 (s, 3H, CH_3).

N.B. Due to poor solubility and its apparent dissociation in THF-d_8, it was not possible to assign signals attributable to **5a** in the $^{13}C\{^1H\}$ or $^{19}F\{^1H\}$ NMR spectrum.

Elemental Analysis: Calculated for $\{C_{38}H_{23}F_{10}NO_2SZn_2\}_2$: C, 51.96; H, 2.64; N, 1.59. Found: C, 51.95; H, 2.53; N, 2.13.

Reaction Optimisation

N-(4-Fluorobenzylidene)-4-methylbenzenesulfonamide **1k** (0.25 mmol) and the corresponding phenyl-zinc reagent were combined in 2.5 mL of anhydrous solvent (toluene or THF) and stirred at room temperature for 2 hours. The reaction was quenched by the addition of MeOH (1 mL) and hexamethylbenzene (0.042, 0.16 equivalents) was added as an internal standard. An aliquot of the reaction mixture was evaporated to dryness and then redissolved in $CDCl_3$ for ^{1}H and ^{19}F NMR spectroscopic analysis, with yields determined by comparison of integrals with the internal standard. **Table S1** summarises the reaction optimisation with various solvents, phenyl-zinc reagents and stoichiometries.

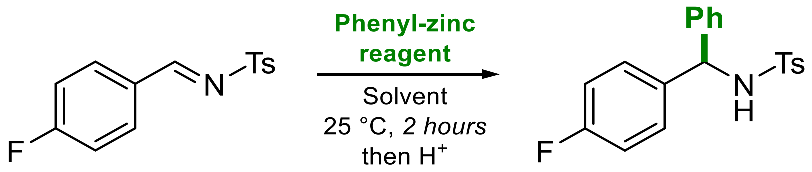

1k **2k**

Entry	Phenyl-zinc reagent	Equivalents	Solvent	Yield (%)
1	$ZnPh_2$	1	THF	12
2	$ZnPh_2$	1	Toluene	80
3	$ZnPh_2$	0.5	Toluene	45
**4**	**$ZnPh_2$ + $Zn(C_6F_5)_2$**	**0.5 each**	**Toluene**	**80**
5	PhZnBr	1	Toluene	0
6	PhZnBr	1	THF	0
7	PhZnBr.LiBr[a]	1	THF	<5
8	$Ph_2Zn.2LiBr$[b]	1	THF	<5
9	PhLi	1	THF	90
10	Ph_3ZnLi[c]	1	THF	70
11	Ph_3ZnLi[c]	0.66	THF	42

Table S1: Reaction optimisation using different phenyl-zinc reagents under different conditions (solvents and stoichiometries). [a] Prepared in situ through the addition of PhLi (1 equiv) to $ZnBr_2$. [b] Prepared in situ through the addition of PhLi (2 equiv) to $ZnBr_2$. [c] Prepared in situ through the addition of PhLi (1 equiv) to $ZnPh_2$.

Comparison of Conditions

Whilst comparable yields of addition product were observed when using 1 equivalent of $ZnPh_2$ (**Table S1**, entry 2) or 0.5 equivalents each of $ZnPh_2$ and $Zn(C_6F_5)_2$ (**Table S1**, entry 4), this was not necessarily true for all diarylzinc reagents tested. **Scheme S1** summaries the yields obtained for each diarylzinc reagent under these two different reaction conditions. In general, the yields obtained when using 0.5 equivalents each of $ZnAr_2$ and $Zn(C_6F_5)_2$ were higher than when simply using 1 equivalent of $ZnAr_2$ in the absence of $Zn(C_6F_5)_2$, particularly for less nucleophilic $ZnAr_2$ compounds.

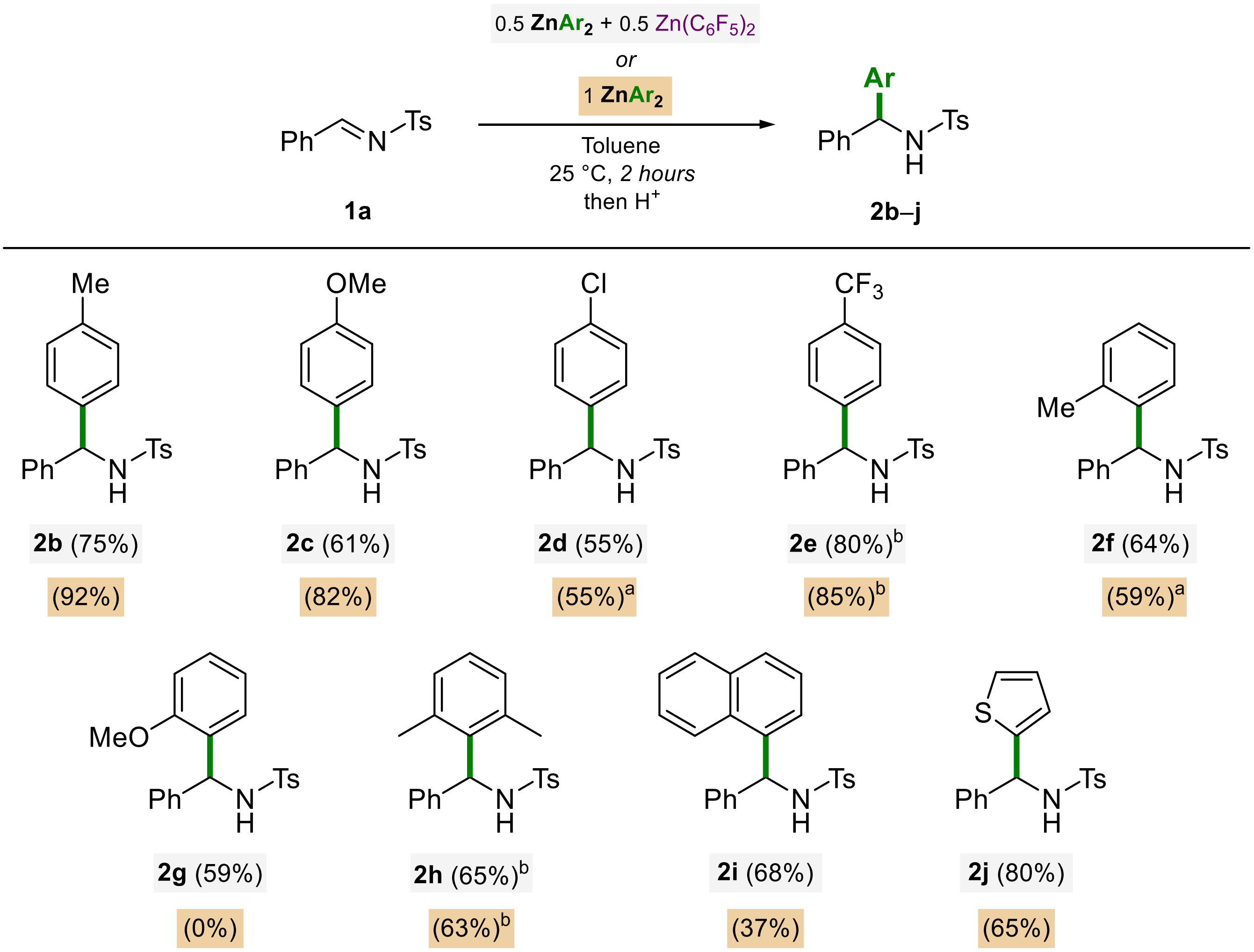

Scheme S1: Comparison of yields obtained when using 0.5 equivalents each of $ZnAr_2$ and $Zn(C_6F_5)_2$ versus 1 equivalent of $ZnAr_2$. [a] Reaction heated to 80 °C for 2 hours. [b] Reaction heated to 80 °C for 20 hours.

Spectroscopic Studies

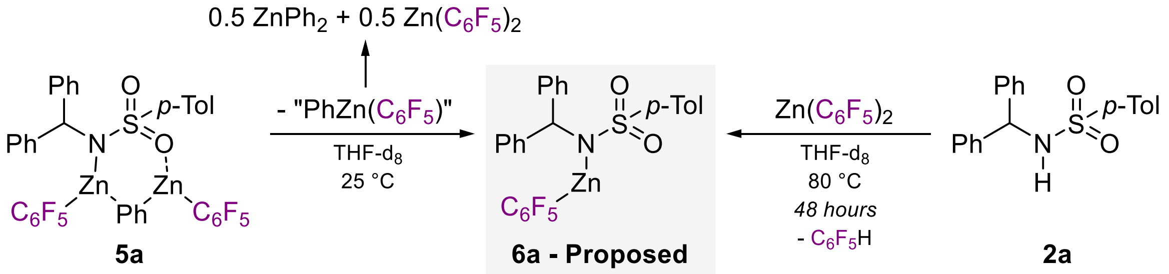

A series of spectroscopic studies were carried out to provide further insights into the reaction mechanism, and to specifically assess the dissociation of "$PhZn(C_6F_5)$" from intermediate **5a**. Compound **5a** is insoluble in toluene-d_8 but dissolution in THF-d_8 gives three major species by ^{19}F NMR spectroscopy attributed to **5a** [δ -117.2 (2F), -159.6 (1F), -163.6 (2F)], **6a** [δ -116.2 (2F), -159.5 (1F), -163.6 (2F)] and $Zn(C_6F_5)_2$ [δ -118.1 (2F), -159.1 (1F), -163.6 (2F)] (**Figures S1–3**). The proposed species **6a** can be rationally prepared by the zincation of **2a** with $Zn(C_6F_5)_2$ – the formation of C_6F_5H confirms the clean deprotonation of **2a**. The formation of **6a** by dissolution of **5a** confirms that "$PhZn(C_6F_5)$" dissociates (to some degree) and

redistributes to its homoleptic components, $ZnPh_2$ and $Zn(C_6F_5)_2$. Given that the quantity of $Zn(C_6F_5)_2$ is significantly larger in the green and blue traces (**Figures S1–3**), it is possible that **6a** further redistributes into homoleptic species, $Zn(C_6F_5)_2$ and the corresponding *bis*-amido-zinc.

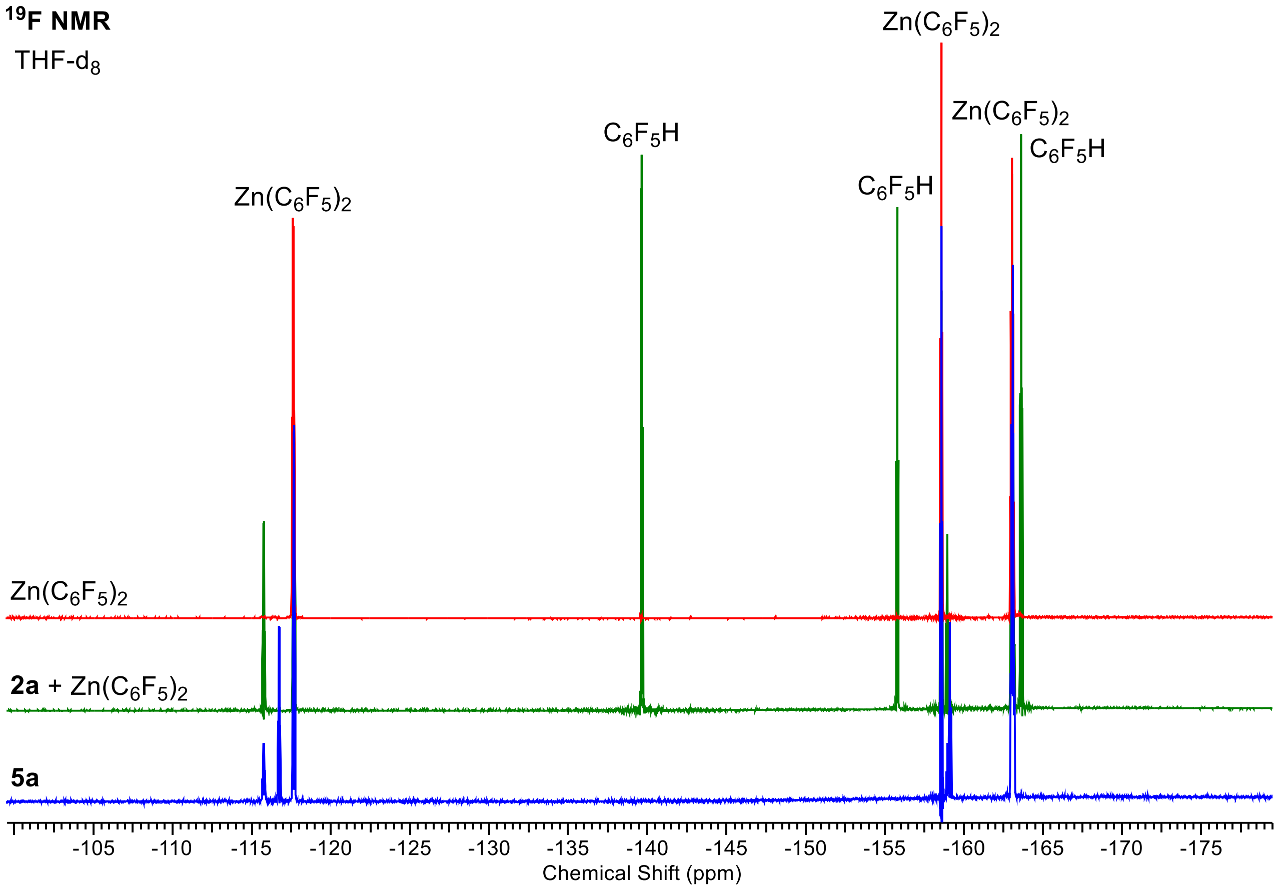

Figure S1: Stacked ^{19}F NMR spectra comparing **5a** (blue trace), the *in situ* formation of **6a** from **2a** (green trace), and $Zn(C_6F_5)_2$ (red trace).

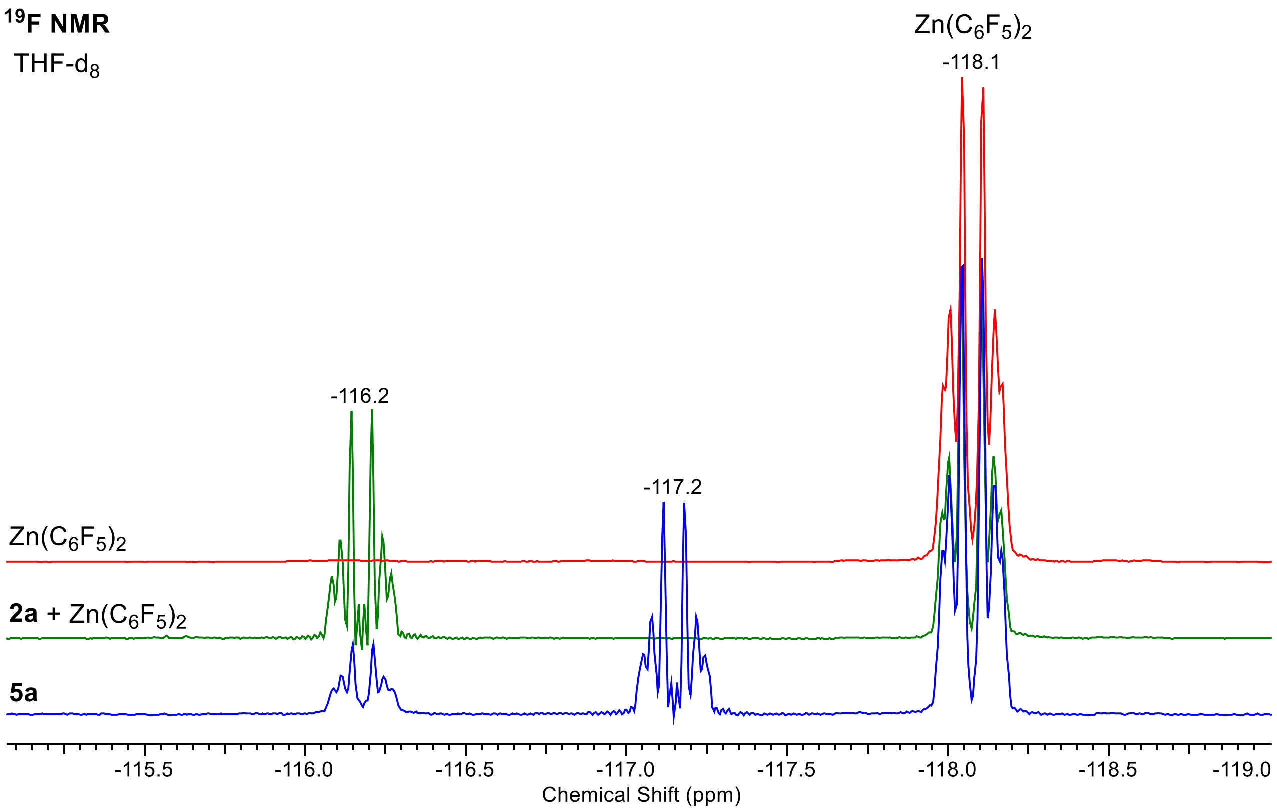

Figure S2: Stacked ^{19}F NMR spectra comparing **5a** (blue trace), the *in situ* formation of **6a** from **2a** (green trace), and $Zn(C_6F_5)_2$ (red trace).

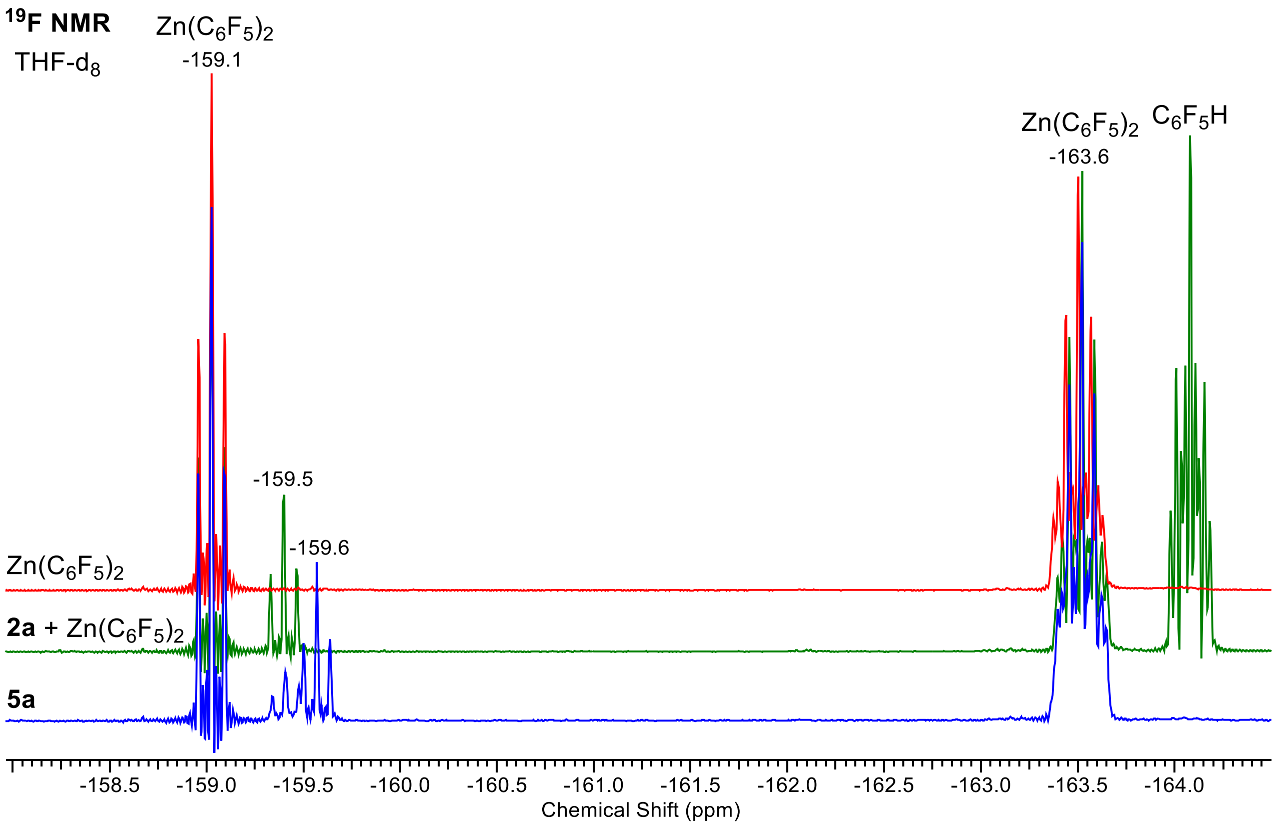

Figure S3: Stacked ^{19}F NMR spectra comparing **5a** (blue trace), the *in situ* formation of **6a** from **2a** (green trace), and $Zn(C_6F_5)_2$ (red trace).

Monitoring these reactions by ^{1}H NMR spectroscopy support that $ZnPh_2$ is liberated from **5a** upon dissolution in THF-d_8 (**Figures S4–5**). The ^{1}H NMR spectra of **5a** and *in situ* generated **6a** both give two signals for in the C*H* and C*H*$_3$ region (**Figure S4 insets**) which are tentatively attributed to **6a** and the corresponding homoleptic bis-amido-zinc species.

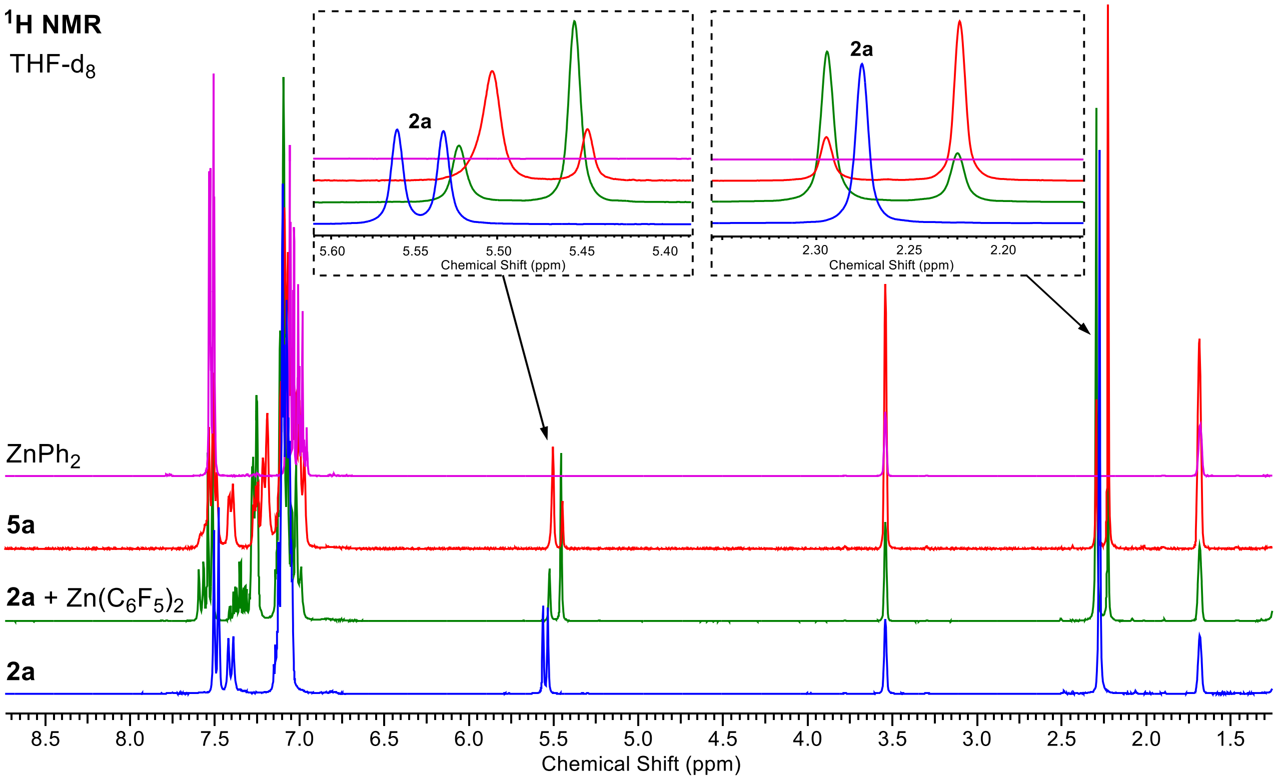

Figure S4: Stacked ^{1}H NMR spectra comparing **2a** (blue trace), the *in situ* formation of **6a** from **2a** (green trace), **5a** (red trace), and $ZnPh_2$ (purple trace).

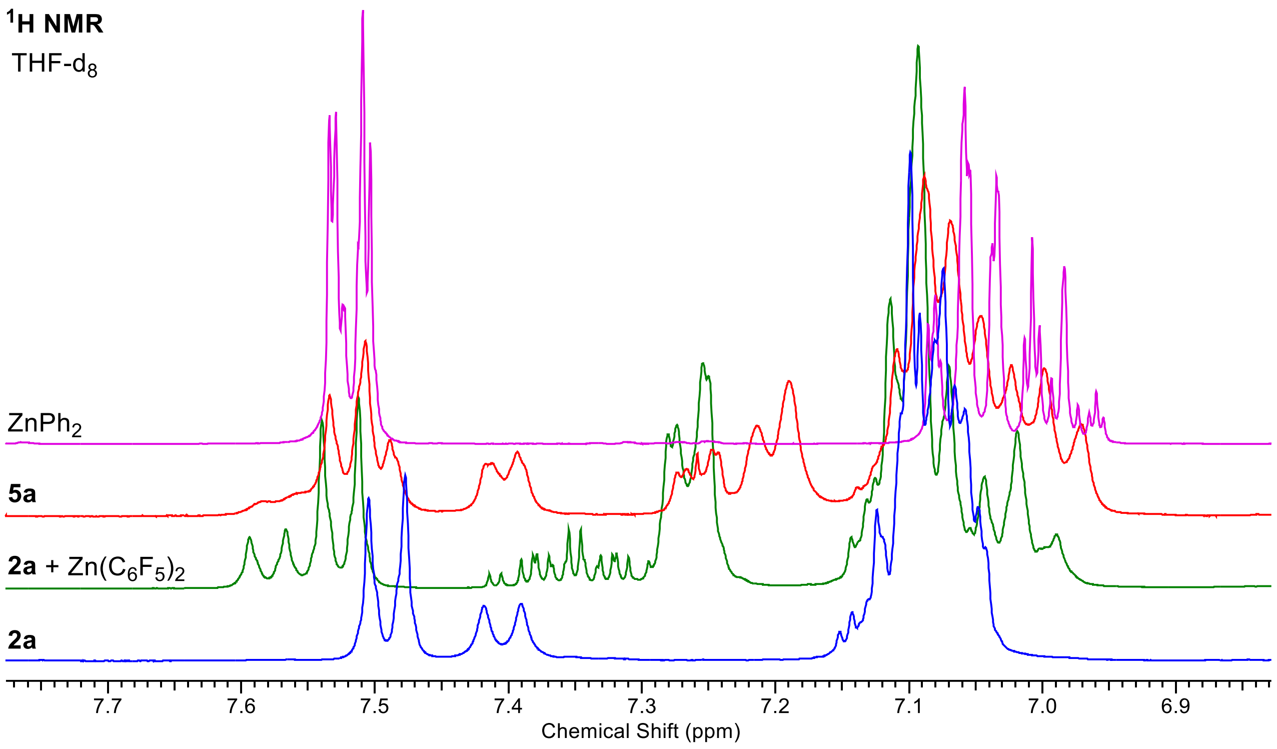

Figure S4: Stacked ^{1}H NMR spectra (aromatic expansion) comparing **2a** (blue trace), the *in situ* formation of **6a** from **2a** (green trace), **5a** (red trace), and $ZnPh_2$ (purple trace).

X-ray Crystallography

The crystal structures have been deposited into the Cambridge Crystallographic Data Centre (CCDC) and have been assigned the following numbers: **3a** – 2251615; **3a.TMEDA** – 2251616; **4a** –2251617; **5a** – 2251618. Selected crystallographic and refinement parameters are presented below (**Tables S2–3**). In all cases, crystals immersed in an inert oil were mounted at ambient conditions and transferred into the nitrogen stream (100 or 173 K).

All measurements were made on a *RIGAKU Synergy S* area-detector diffractometer using mirror optics monochromated Cu Kα radiation (λ = 1.54184 Å). Data reduction was performed using the *CrysAlisPro* program.[14] The intensities were corrected for Lorentz and polarization effects, and an absorption correction based on the Gaussian method using SCALE3 ABSPACK in *CrysAlisPro* was applied. The structure was solved by direct methods or intrinsic phasing using *SHELXT*,[15] which revealed the positions of all non-hydrogen atoms of the compounds. All non-hydrogen atoms were refined anisotropically. H-atoms were assigned in geometrically calculated positions and refined using a riding model where each H-atom was assigned a fixed isotropic displacement parameter with a value equal to 1.2Ueq of its parent atom (1.5Ueq for methyl groups). Refinement of the structure was carried out on F^2 using full-matrix least-squares procedures, which minimized the function $\Sigma w(F_o^2 - F_c^2)^2$. The weighting scheme was based on counting statistics and included a factor to downweight the intense reflections. All calculations were performed using the *SHELXL-2014/7*[16] program in OLEX2.[17]

For compound **3a**, areas containing disordered solvents were found where a satisfactory solvent model could not be achieved, therefore, a solvent mask was used to include the contribution of electron density

found in void areas into the calculated structure factor. The total number of electrons found in the void areas was 89 electrons – this corresponds to a disordered toluene molecule.

For compound **3a.TMEDA**, areas containing disordered solvents were found where a satisfactory solvent model could not be achieved, therefore, a solvent mask was used to include the contribution of electron density found in void areas into the calculated structure factor. The total number of electrons found in the void areas was 202 electrons – this corresponds to a disordered toluene or hexane molecule.

Compound	**3a**	**3a.TMEDA**
Identification code	22EH167_TK-137	20EH166_JN187
Empirical formula	$C_{104}H_{92}N_4O_8S_4Zn_4$	$C_{32}H_{39}N_3O_2SZn$
Formula weight	1915.53	595.09
Temperature/K	173.00(10)	100.0(3)
Crystal system	triclinic	monoclinic
Space group	P-1	C2/c
a/Å	13.5602(3)	28.6033(8)
b/Å	13.8377(3)	9.8468(3)
c/Å	14.8499(2)	23.6134(5)
α/°	70.109(2)	90
β/°	81.732(2)	94.965(3)
γ/°	72.621(2)	90
Volume/Å^3	2497.85(9)	6625.8(3)
Z	1	8
ρ_{calc}g/cm^3	1.273	1.193
μ/mm^{-1}	2.305	1.846
F(000)	992	2512
Crystal size/mm^3	0.157 × 0.075 × 0.033	0.326 × 0.215 × 0.1
Radiation	Cu Kα (λ = 1.54184)	CuKα (λ = 1.54184)
2Θ range for data collection/°	6.336 to 149.006	6.204 to 161.124
Index ranges	-16 ≤ h ≤ 15, -17 ≤ k ≤ 17, -18 ≤ l ≤ 18	-36 ≤ h ≤ 36, -12 ≤ k ≤ 12, -24 ≤ l ≤ 30
Reflections collected	96707	56084
Independent reflections	10200 [R_{int} = 0.0657, R_{sigma} = 0.0240]	7182 [R_{int} = 0.0809, R_{sigma} = 0.0358]
Data/restraints/parameters	10200/144/617	7182/0/357
Goodness-of-fit on F^2	1.071	1.051
Final R indexes [I>=2σ (I)]	R_1 = 0.0531, wR_2 = 0.1477	R_1 = 0.0678, wR_2 = 0.1727
Final R indexes [all data]	R_1 = 0.0570, wR_2 = 0.1506	R_1 = 0.0839, wR_2 = 0.1872
Largest diff. peak/hole / e Å^{-3}	0.74/-1.00	0.98/-0.86

Table S2: Crystal data and structure refinement details for **3a** and **3a.TMEDA**.

Compound	**4a**	**5a**
Identification code	20EH188_JN200	22EH166_TK-138
Empirical formula	$C_{26}H_{13}F_{10}NO_2SZn$	$C_{76}H_{46}F_{20}N_2O_4S_2Zn_4$
Formula weight	658.8	1756.75
Temperature/K	173.00(10)	173.00(10)
Crystal system	monoclinic	monoclinic
Space group	$P2_1/n$	$P2_1/n$
a/Å	11.67120(10)	12.61445(7)
b/Å	16.99090(10)	17.36835(10)
c/Å	12.47900(10)	15.87255(9)
α/°	90	90
β/°	92.2700(10)	93.2751(5)
γ/°	90	90
Volume/Å^3	2472.70(3)	3471.87(3)
Z	4	2
ρ_{calc}g/cm^3	1.77	1.68
μ/mm^{-1}	3.133	3.081
F(000)	1312	1760
Crystal size/mm^3	0.333 × 0.162 × 0.137	0.407 × 0.225 × 0.158
Radiation	Cu Kα (λ = 1.54184)	Cu Kα (λ = 1.54184)
2Θ range for data collection/°	8.796 to 159.616	7.552 to 148.988
Index ranges	-14 ≤ h ≤ 14, -21 ≤ k ≤ 21, -15 ≤ l ≤ 15	-15 ≤ h ≤ 15, -21 ≤ k ≤ 21, -19 ≤ l ≤ 19
Reflections collected	45935	69714
Independent reflections	45935 [R_{int} = ?, R_{sigma} = 0.0100]	7101 [R_{int} = 0.0337, R_{sigma} = 0.0139]
Data/restraints/parameters	45935/0/372	7101/0/489
Goodness-of-fit on F^2	1.154	1.073
Final R indexes [I>=2σ (I)]	R_1 = 0.0430, wR_2 = 0.1517	R_1 = 0.0266, wR_2 = 0.0684
Final R indexes [all data]	R_1 = 0.0536, wR_2 = 0.2183	R_1 = 0.0267, wR_2 = 0.0685
Largest diff. peak/hole / e Å^{-3}	0.83/-0.65	0.34/-0.34

Table S3: Crystal data and structure refinement details for **4a** and **5a**.

Molecular Structure of 3a

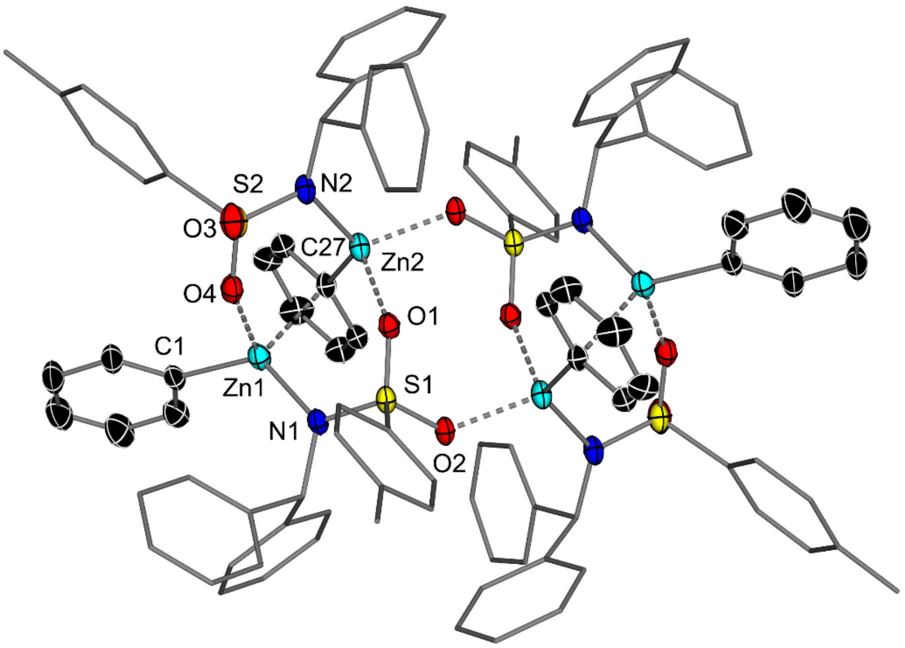

Figure S5: Molecular structure of **3a**. Thermal ellipsoids shown at 30% probability. Hydrogen atoms omitted and aryl-substituents not on Zn shown as wireframe for clarity. Selected bond lengths [Å]: Zn1–C1 1.983(3); Zn1···C27 2.364(3); Zn1–N1 2.006(3); O4···Zn1 2.122(2); Zn2–C27 2.003(3); Zn2–N2 1.965(2); O1···Zn2 2.112(2); O2···Zn2 2.033(2). Selected bond angles [°]: C1–Zn1–N1 127.1(1); N2–Zn2–C27 124.4(1).

Molecular Structure of 3a.TMEDA

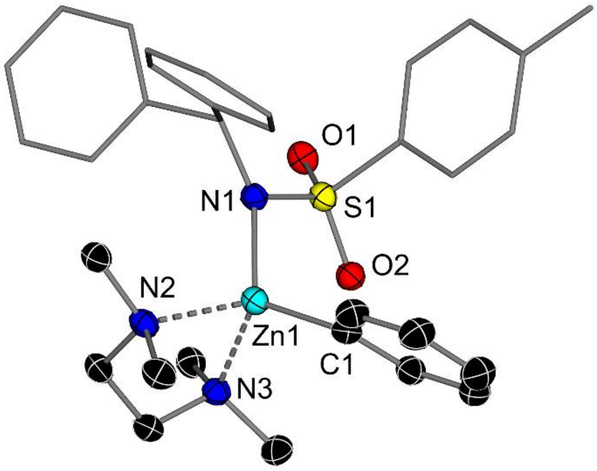

Figure S6: Molecular structure of **3a.TMEDA**. Thermal ellipsoids shown at 30% probability. Hydrogen atoms omitted and aryl-substituents not on Zn shown as wireframe for clarity. Selected bond lengths [Å]: Zn1–C1 2.018(4); Zn1–N1 2.055(3); N2···Zn1 2.196(3); N3···Zn1 2.176(3). Selected bond angles [°]: C1–Zn1–N1 120.9(1).

Molecular Structure of 4a

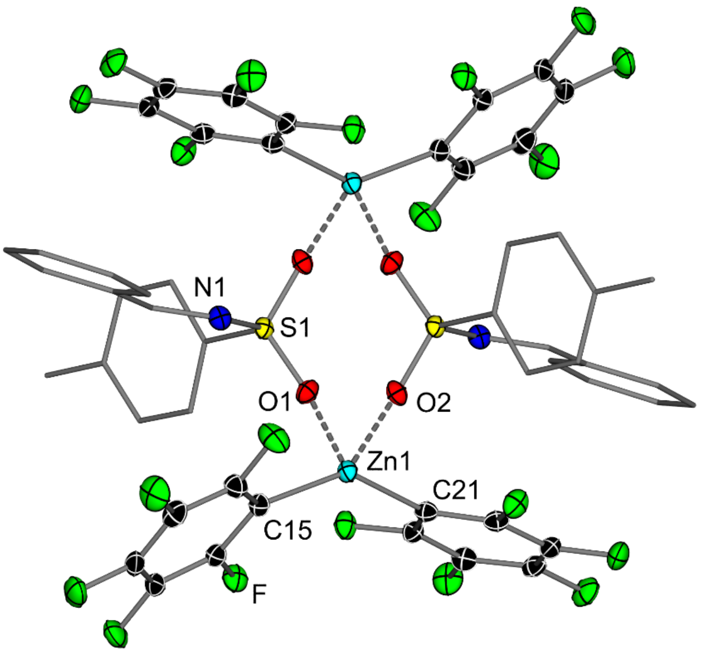

Figure S7: Molecular structure of **4a**. Thermal ellipsoids shown at 30% probability. Hydrogen atoms omitted and aryl-substituents not on Zn shown as wireframe for clarity. Selected bond lengths [Å]: Zn1–C15 1.974(5); Zn1–C21 1.987(5); O1⋯Zn1 2.200(3); O2⋯Zn1 2.129(2). Selected bond angles [°]: C15–Zn1–C21 138.8(2).

Molecular Structure of 5a

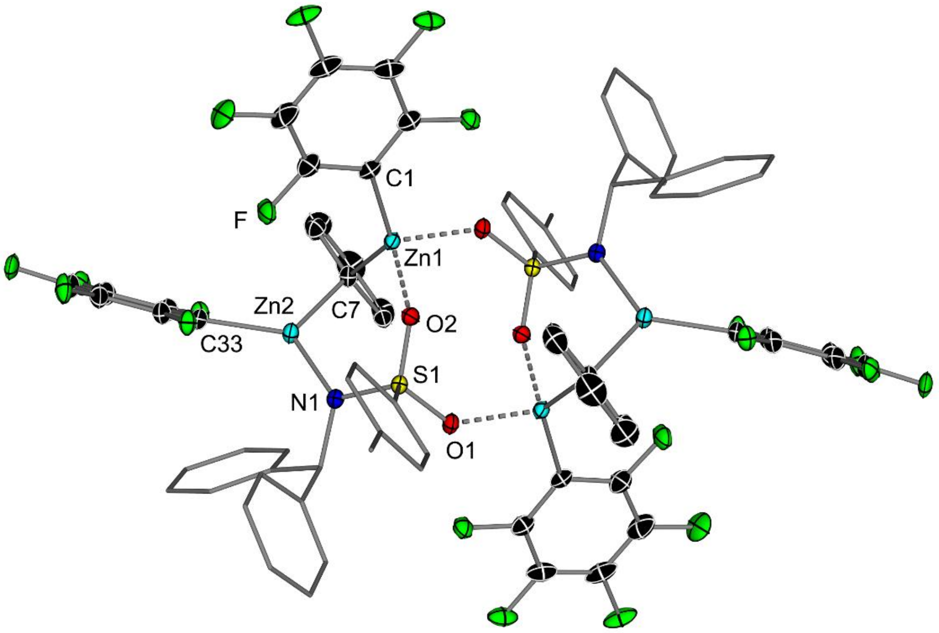

Figure S8: Molecular structure of **5a**. Thermal ellipsoids shown at 30% probability. Hydrogen atoms omitted and aryl-substituents not on Zn shown as wireframe for clarity. Selected bond lengths [Å]: Zn1–C1 1.989(2); Zn1–C7 2.058(2); O1⋯Zn1 2.055(1); O2⋯Zn1 2.066(1); Zn2–C7 2.111(2); Zn2–C33 1.983(2); Zn2–N1 1.995(1). Selected bond angles [°]: C1–Zn1–C7 131.41(7); Zn1–C7–Zn2 90.51(6); C7–Zn2–C33 118.22(7); C7–Zn2–N1 115.27(6); N1–Zn2–C33 126.50(6).

NMR Spectra of Reported Compounds

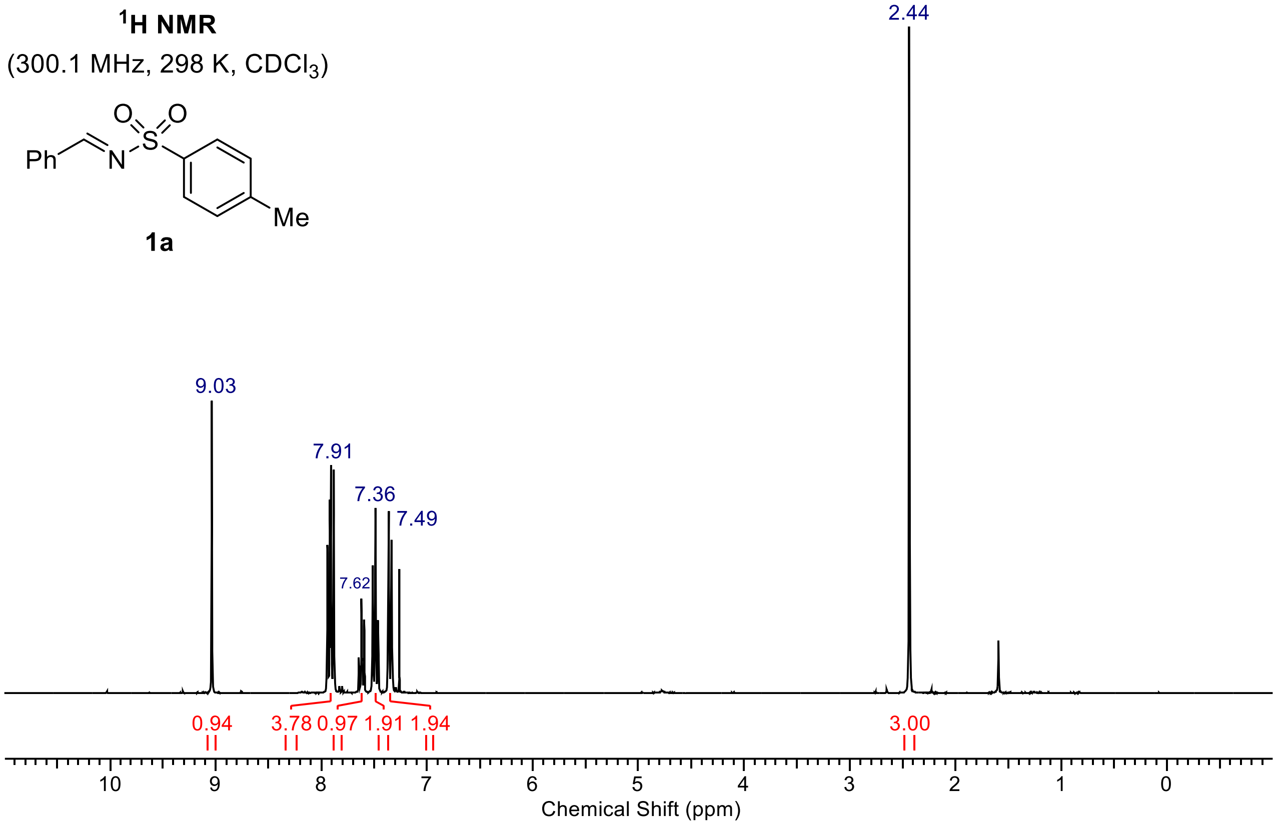

Spectra S1: ^{1}H NMR spectrum of **1a**.

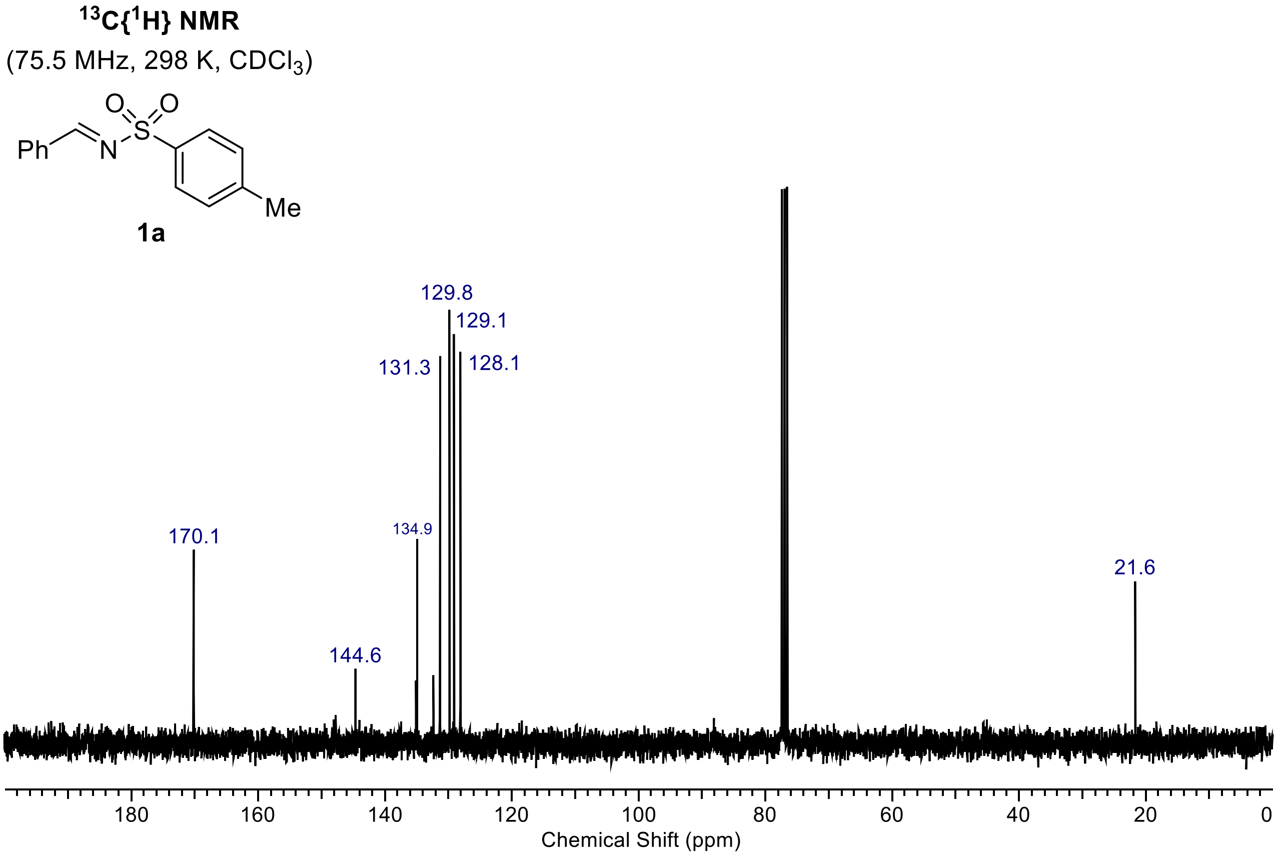

Spectra S2: ^{13}C{^{1}H} NMR spectrum of **1a**.

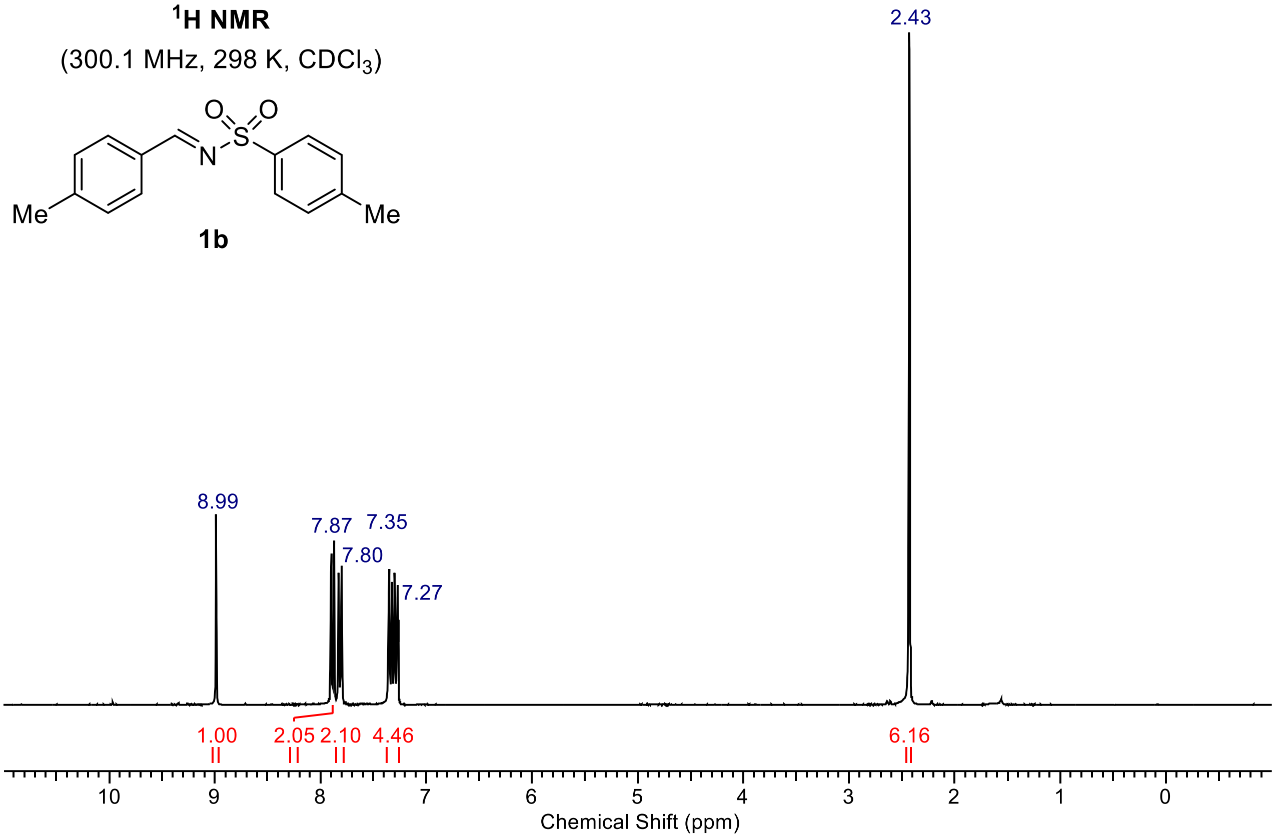

Spectra S3: ^{1}H NMR spectrum of **1b**.

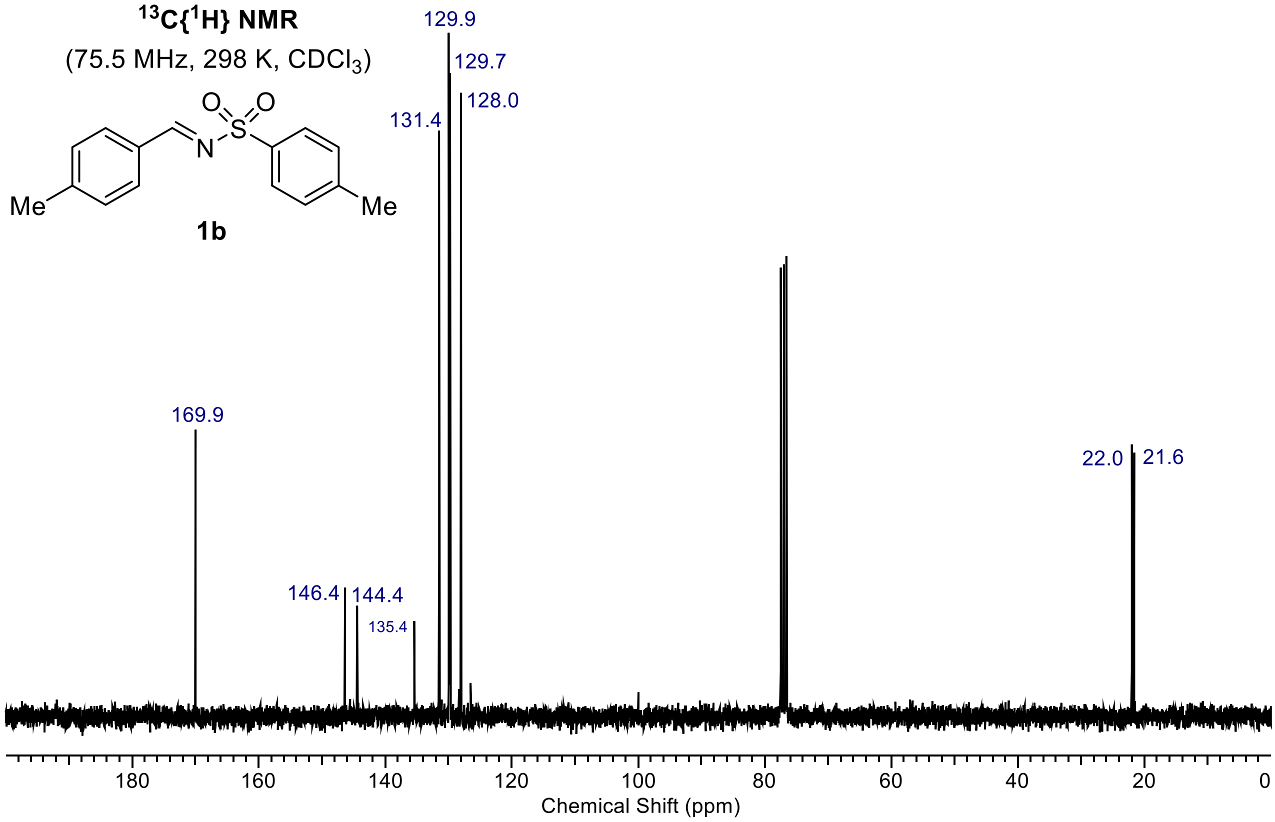

Spectra S4: ^{13}C{^{1}H} NMR spectrum of **1b**.

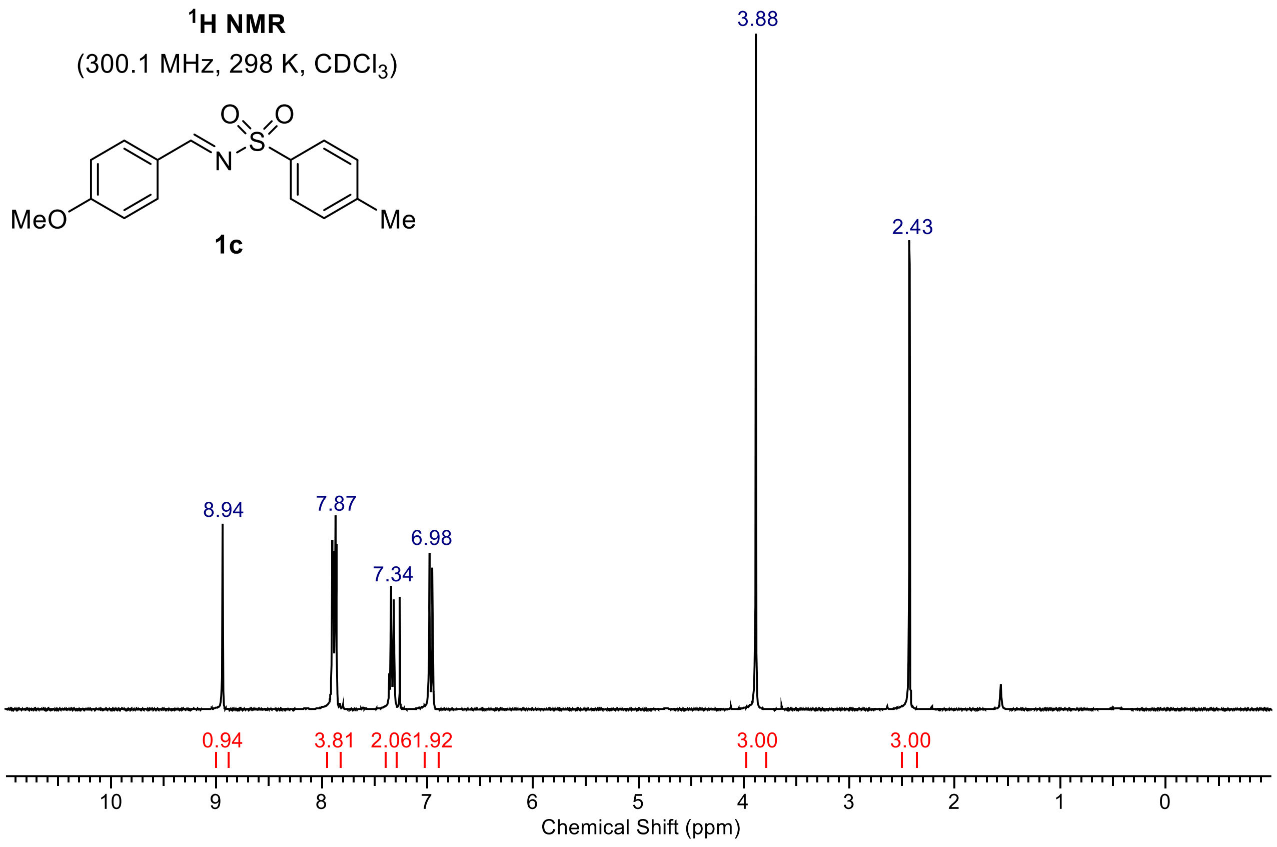

Spectra S5: ^{1}H NMR spectrum of **1c**.

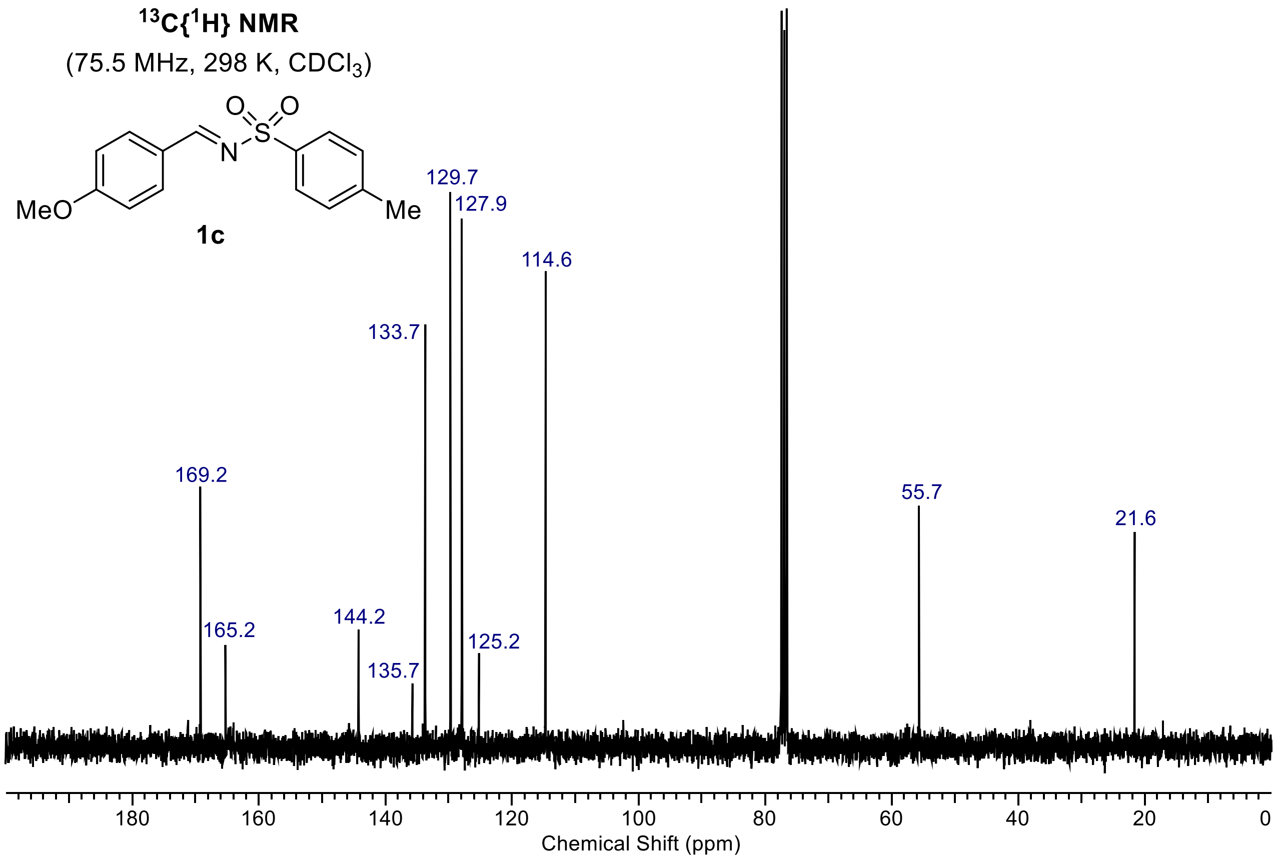

Spectra S6: $^{13}C\{^{1}H\}$ NMR spectrum of **1c**.

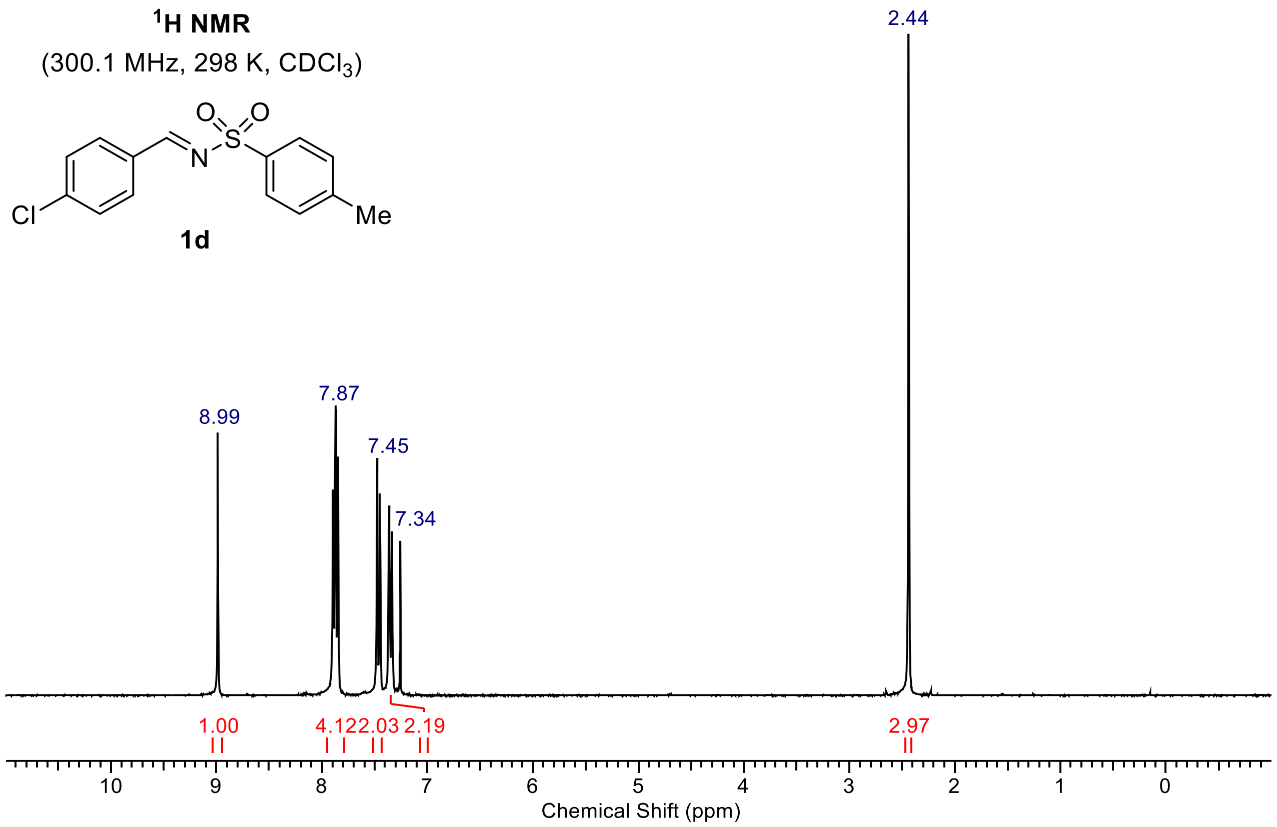

Spectra S7: ^{1}H NMR spectrum of **1d**.

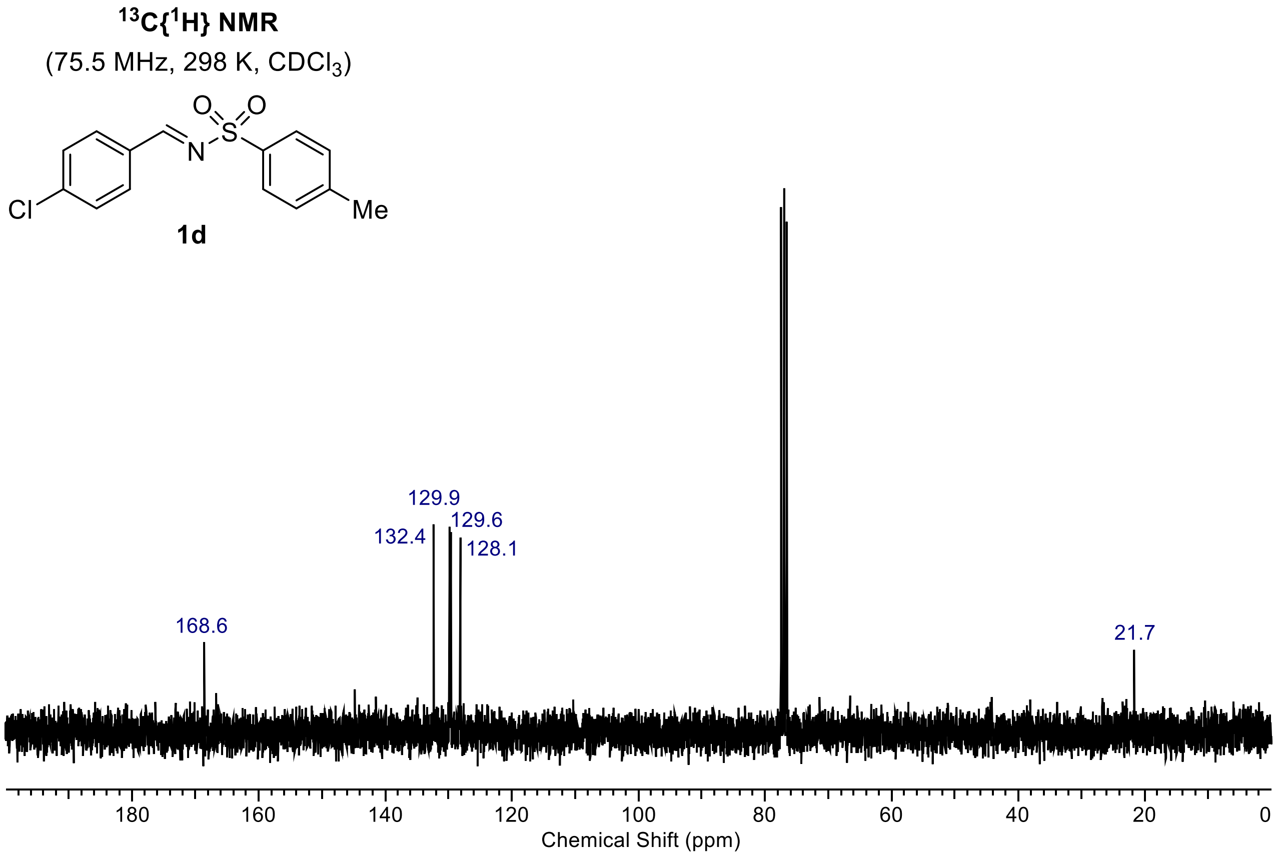

Spectra S8: ^{13}C{^{1}H} NMR spectrum of **1d**.

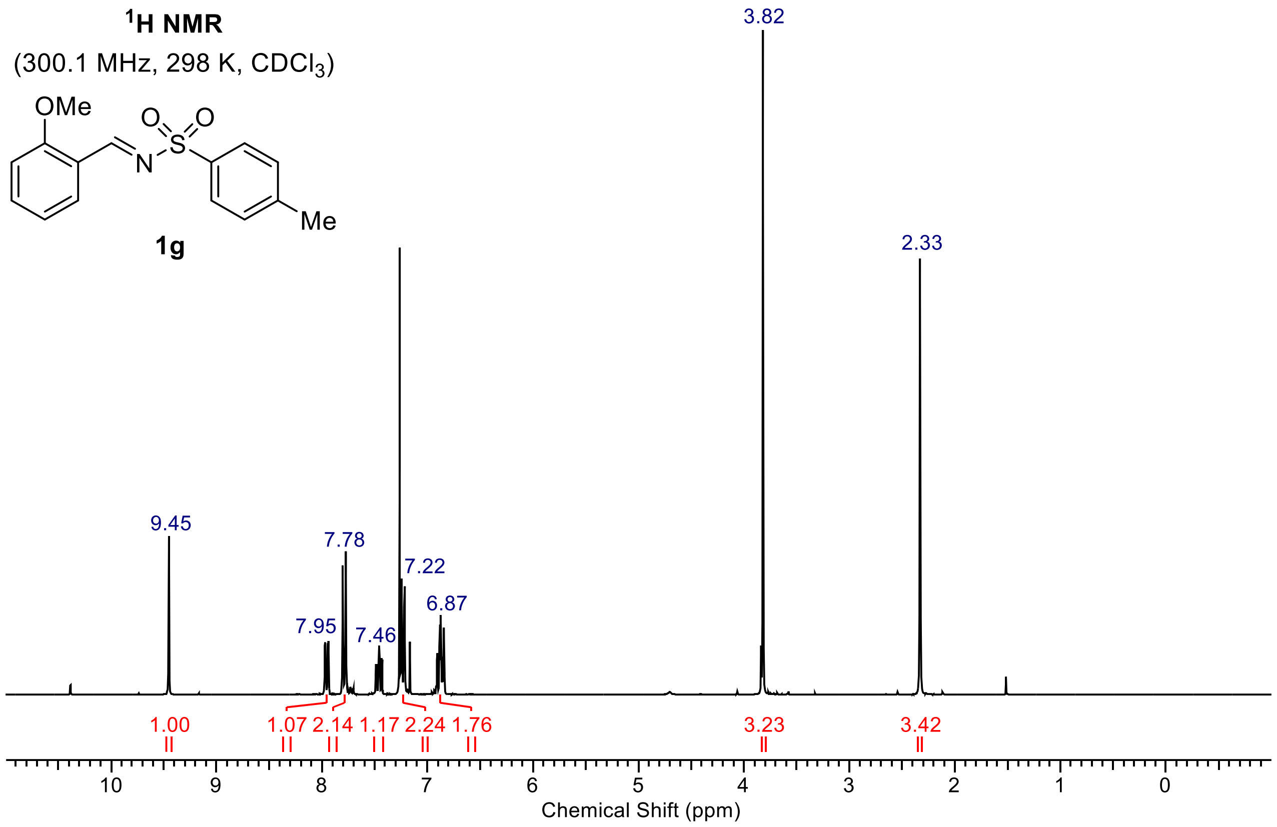

Spectra S9: ^{1}H NMR spectrum of **1g**.

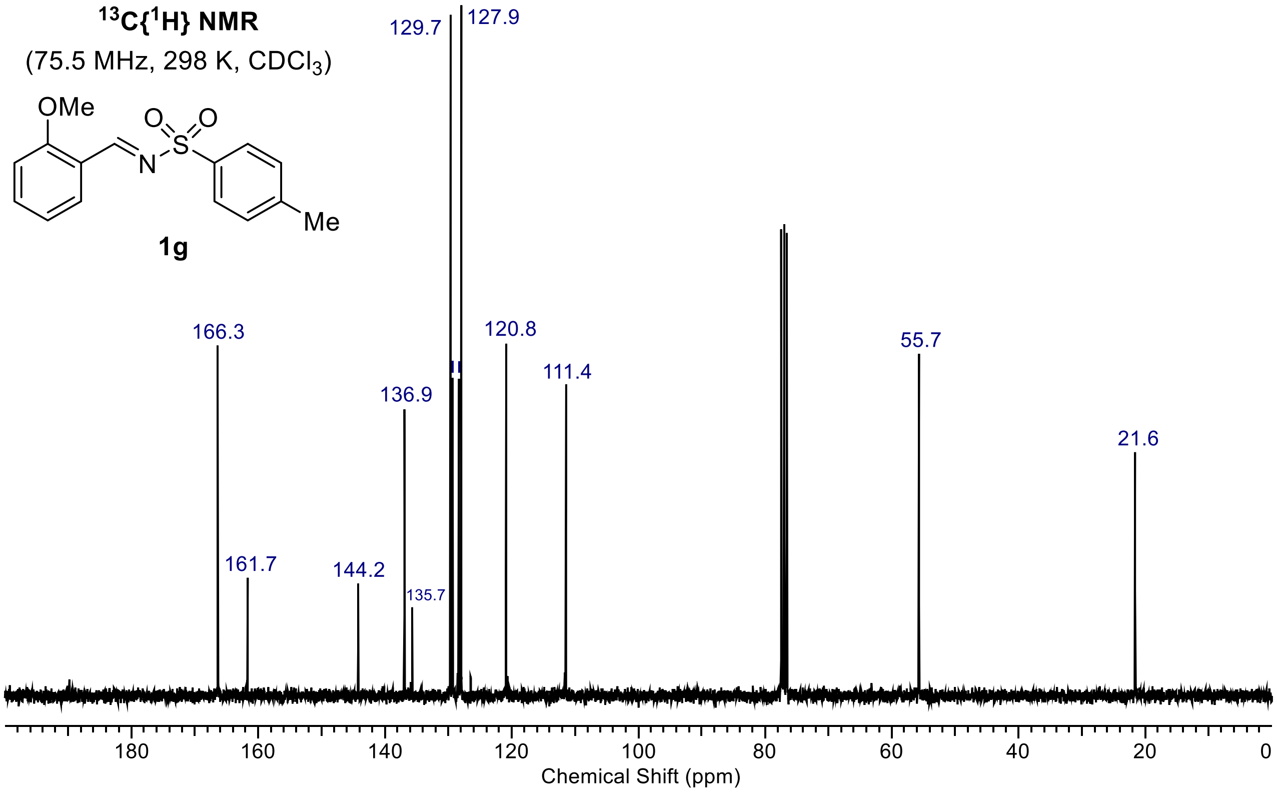

Spectra S10: ^{13}C{^{1}H} NMR spectrum of **1g**.

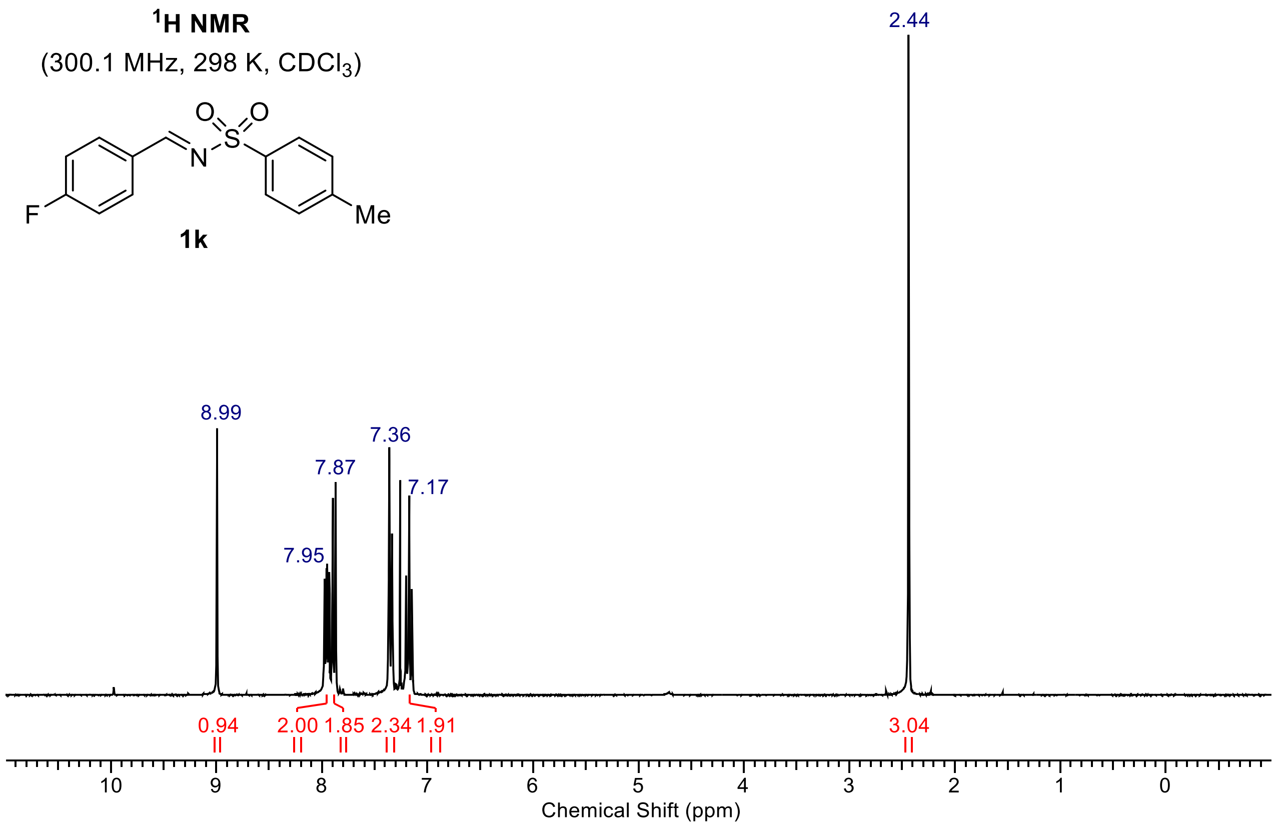

Spectra S11: ^{1}H NMR spectrum of **1k**.

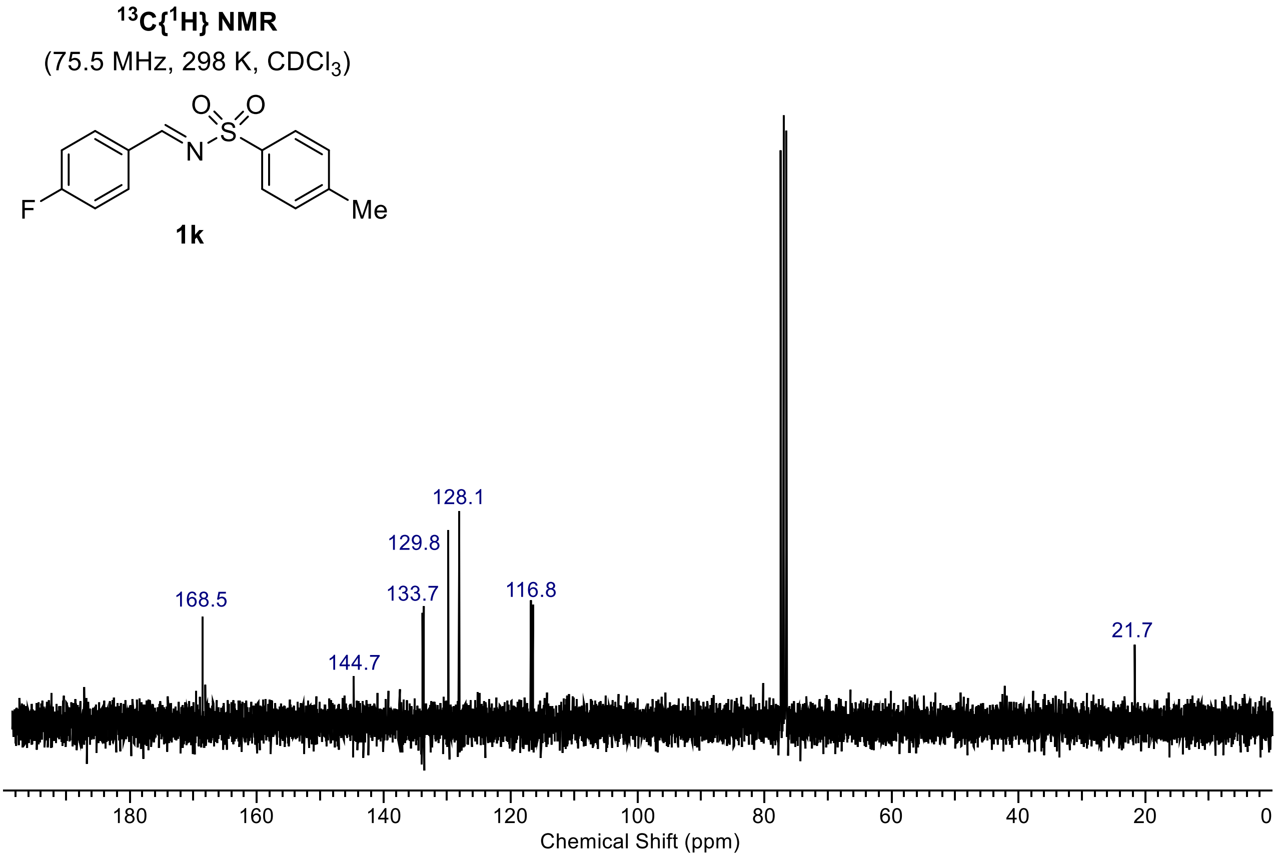

Spectra S12: ^{13}C{^{1}H} NMR spectrum of **1k**.

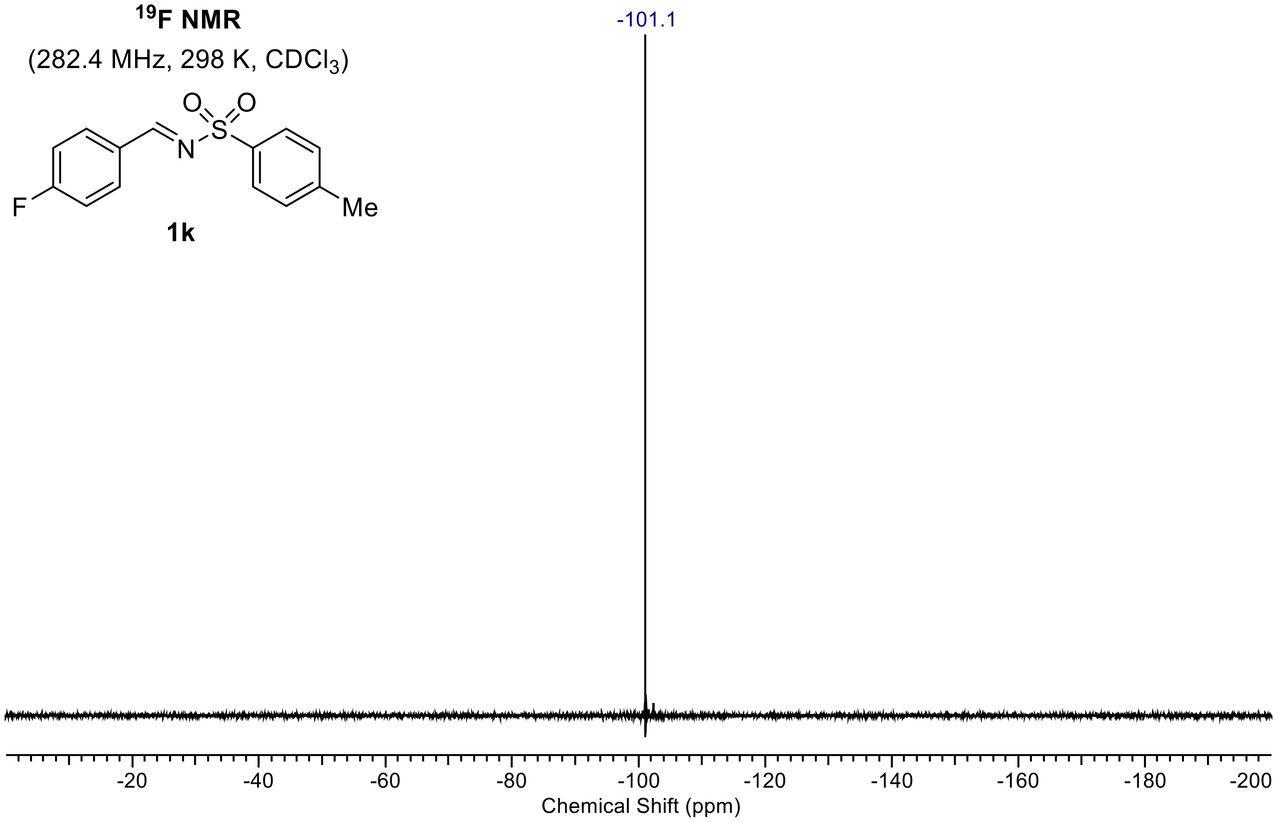

Spectra S13: ^{19}F NMR spectrum of **1k**.

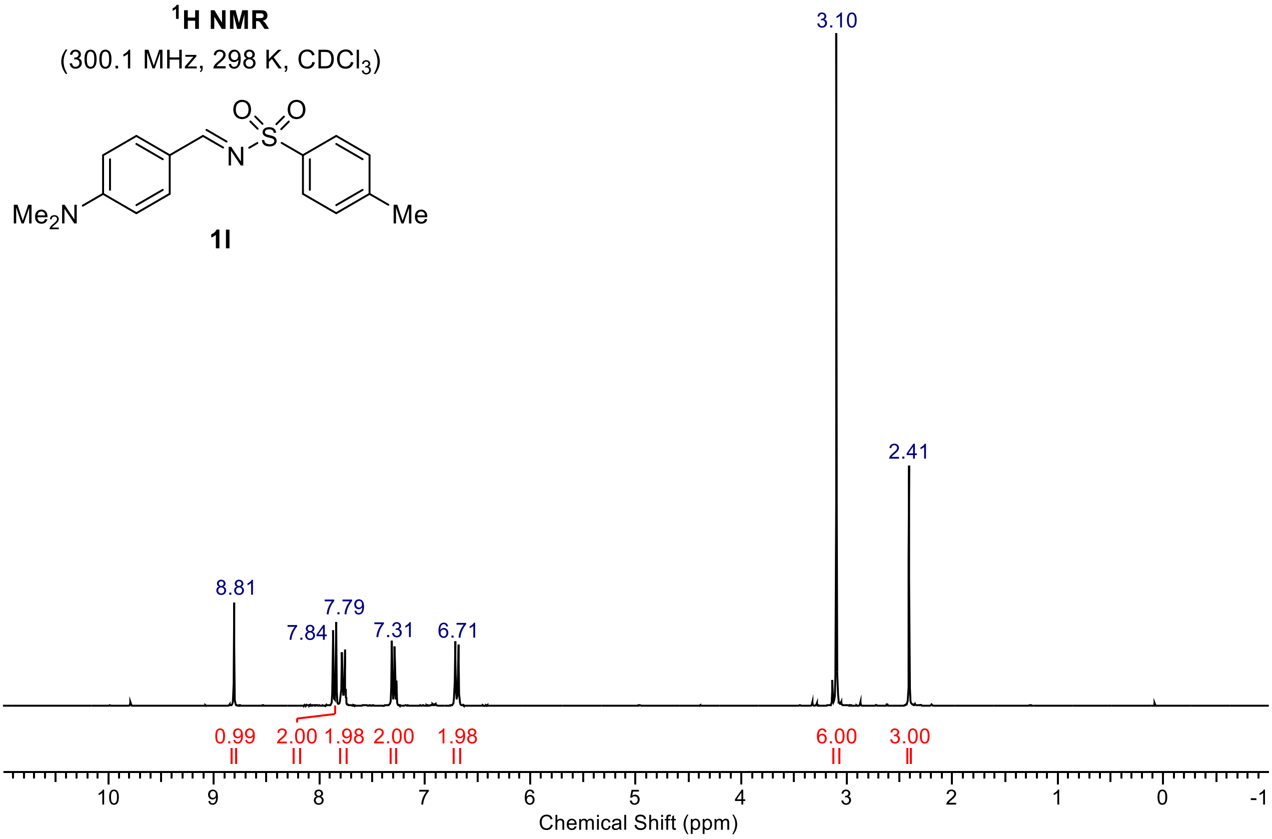

Spectra S14: ^{1}H NMR spectrum of **1l**.

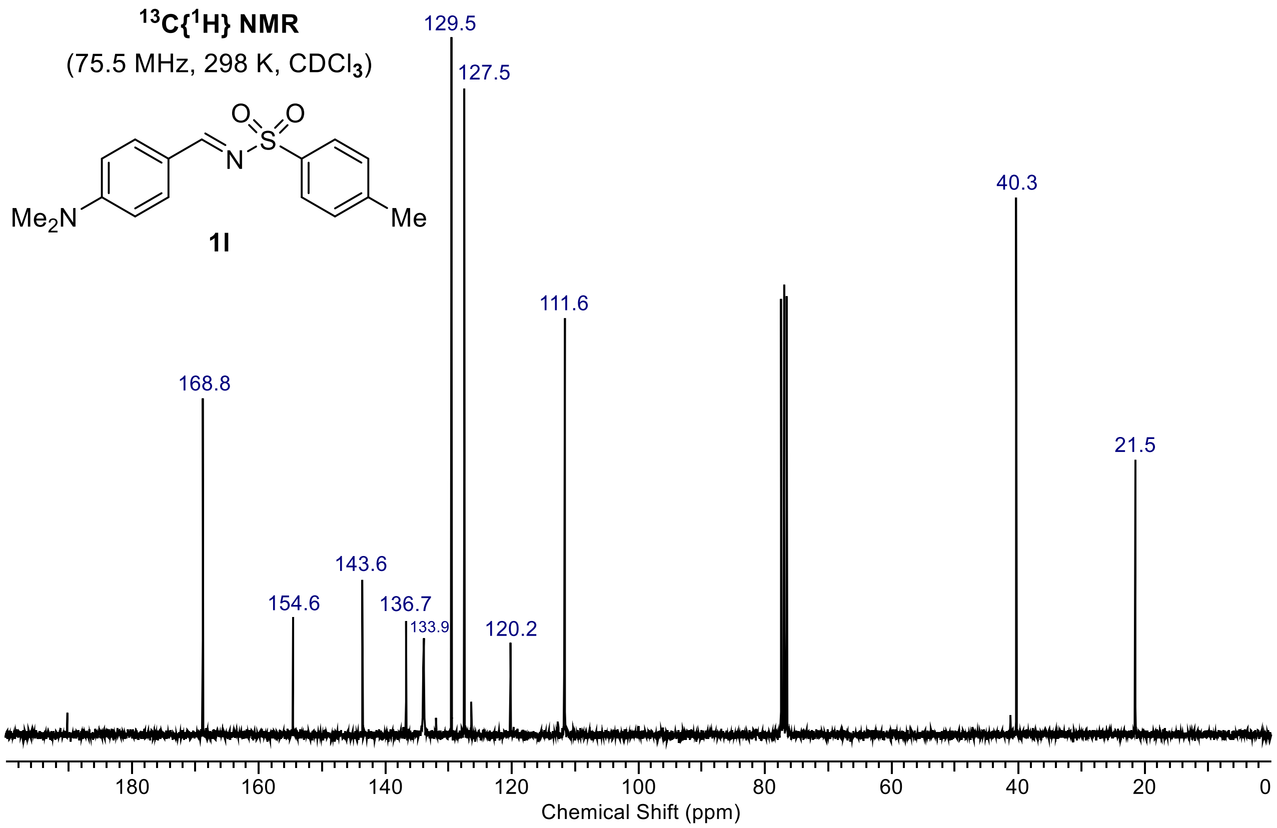

Spectra S15: ^{13}C{^{1}H} NMR spectrum of **1l**.

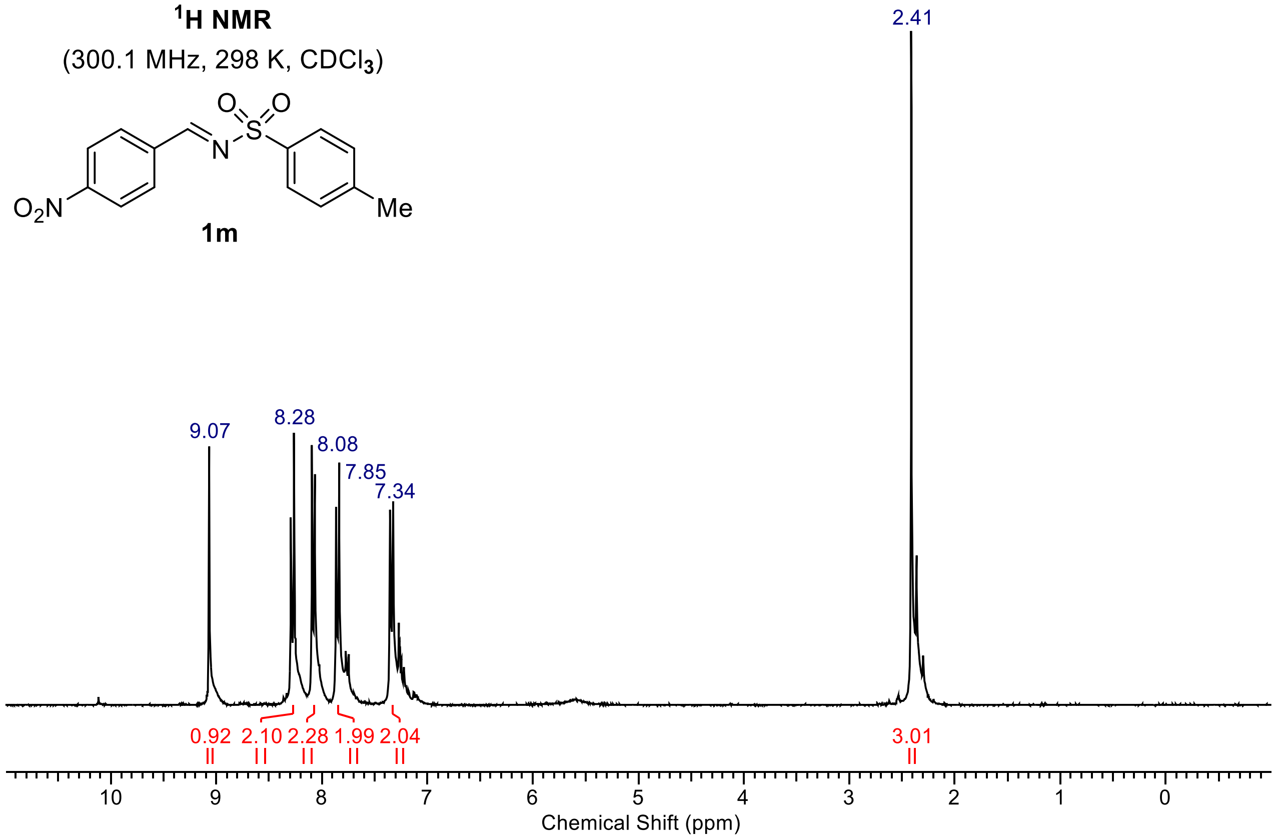

Spectra S16: ^{1}H NMR spectrum of **1m**.

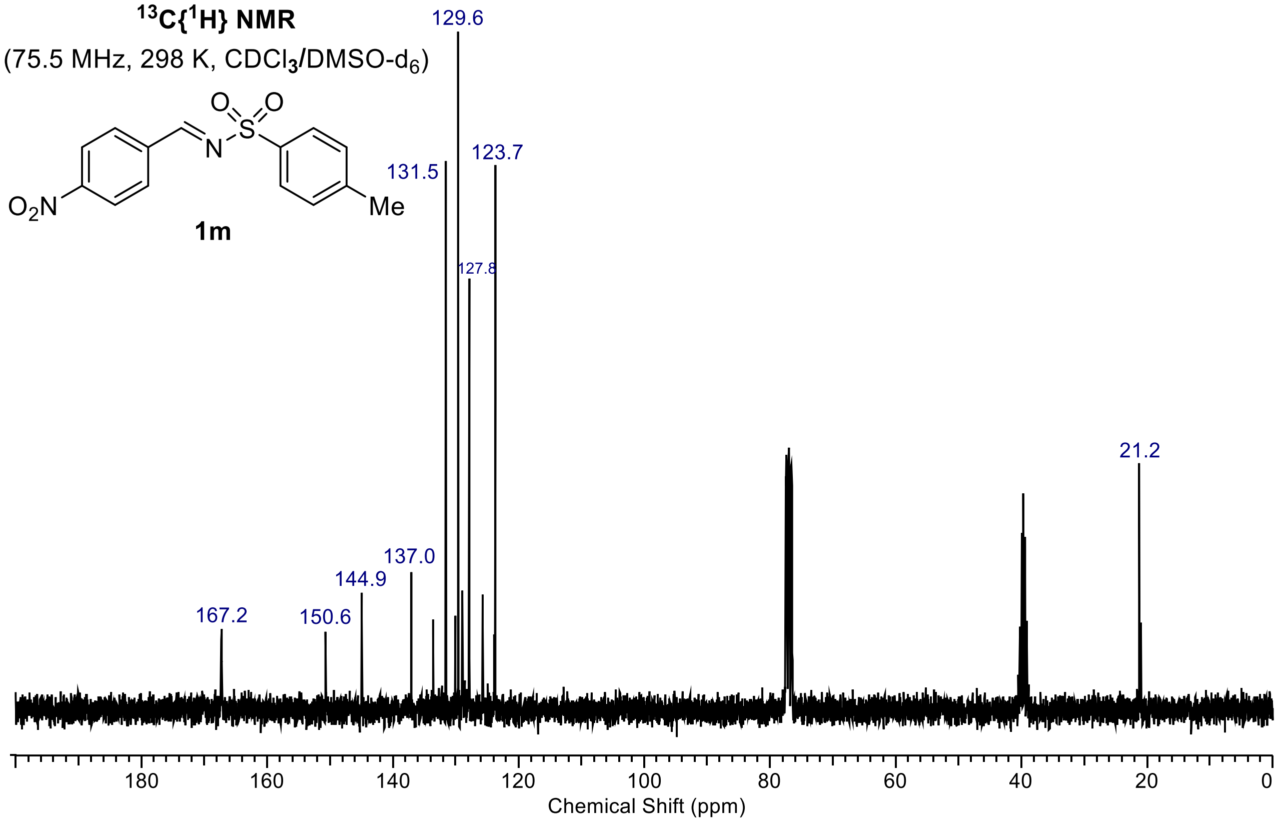

Spectra S17: $^{13}C\{^1H\}$ NMR spectrum of **1m**.

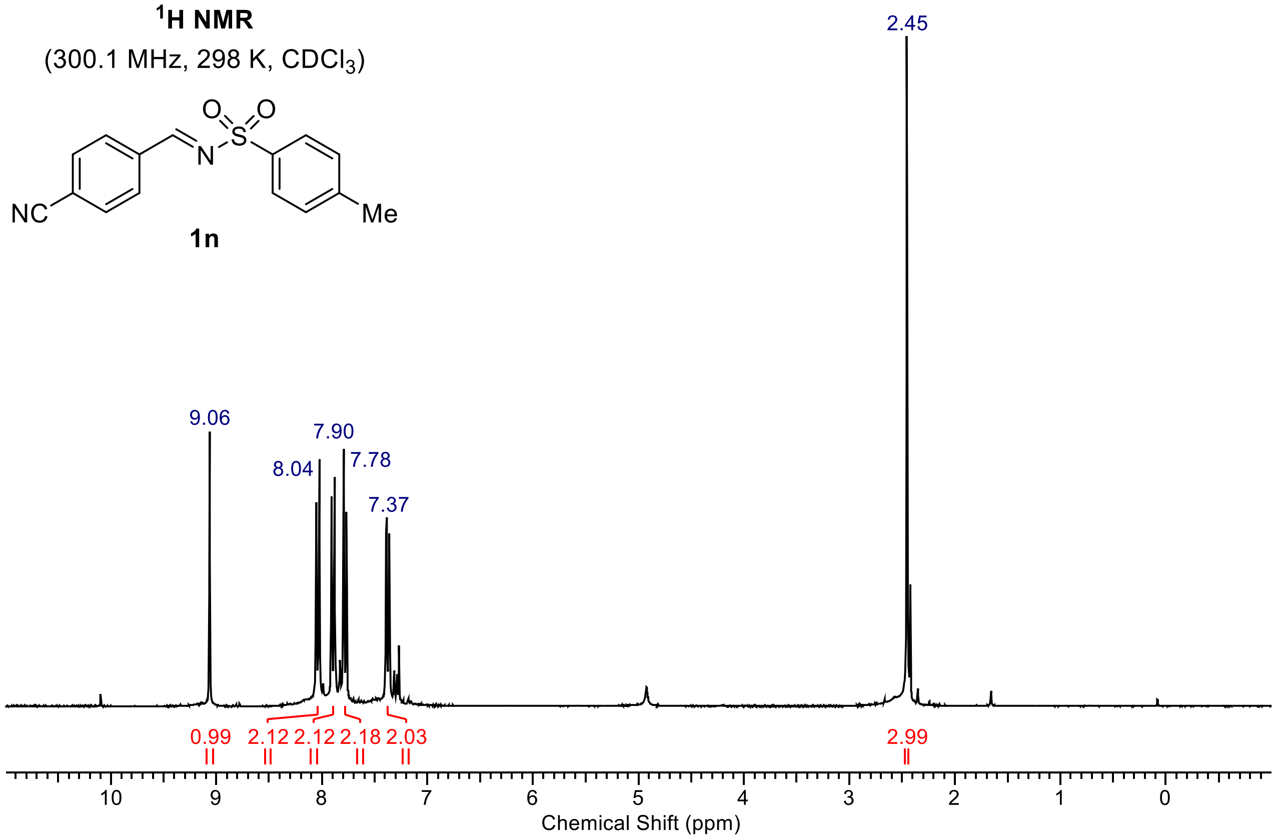

Spectra S18: ^1H NMR spectrum of **1n**.

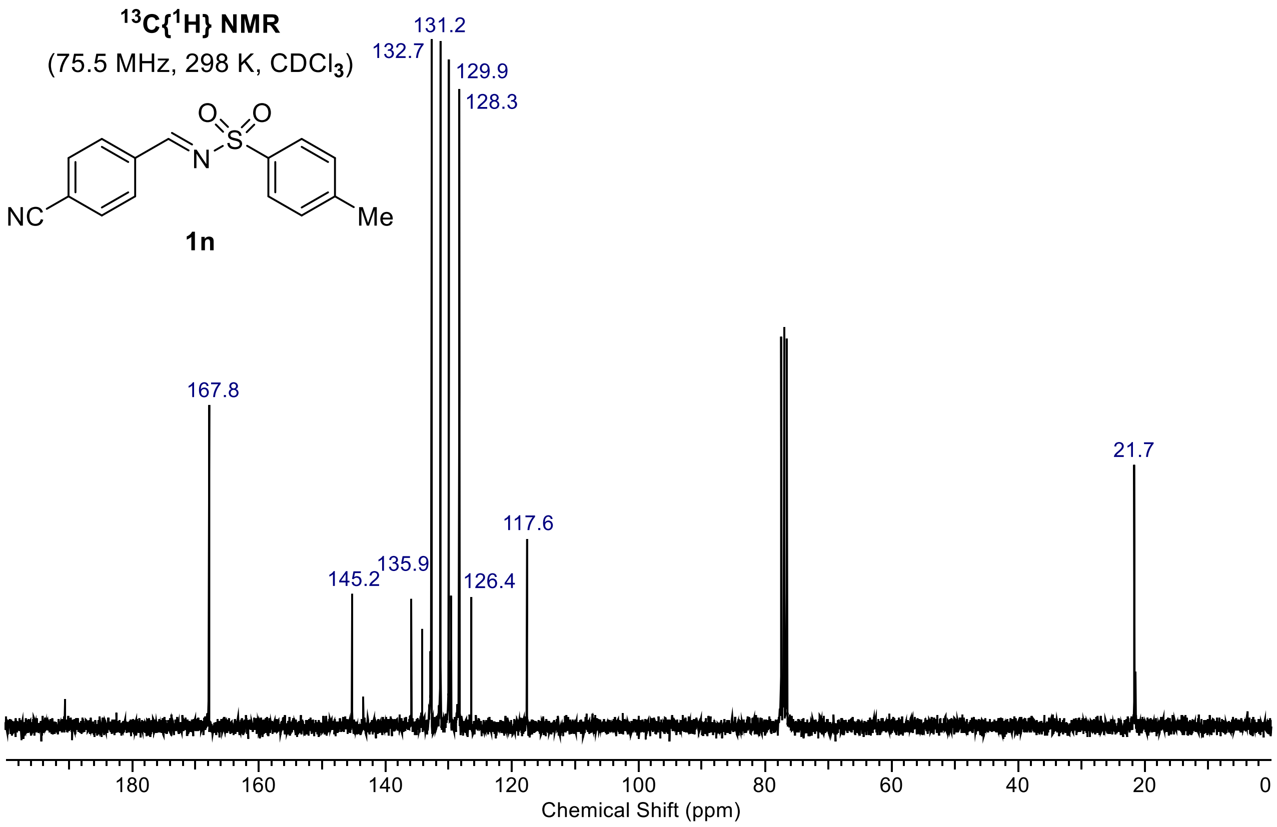

Spectra S19: $^{13}C\{^1H\}$ NMR spectrum of **1n**.

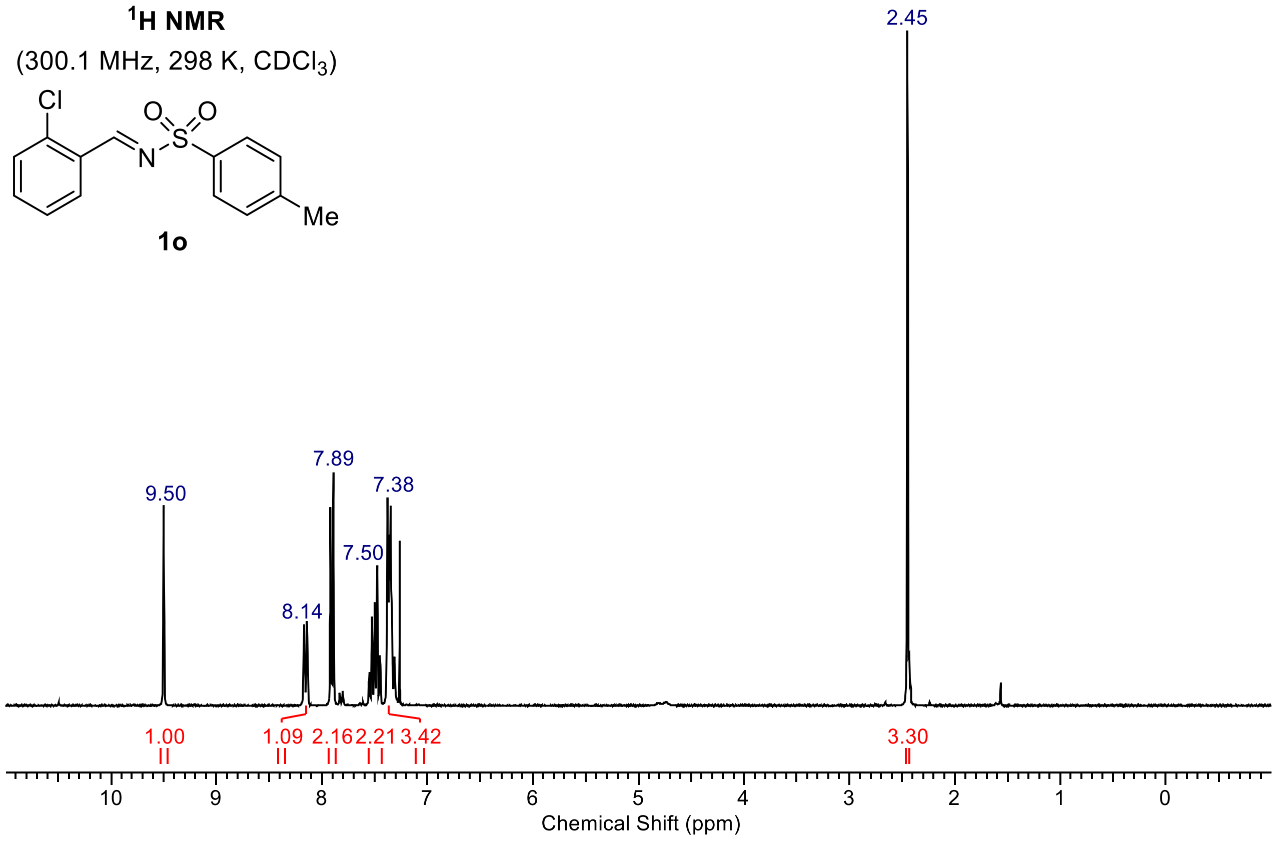

Spectra S20: ^1H NMR spectrum of **1o**.

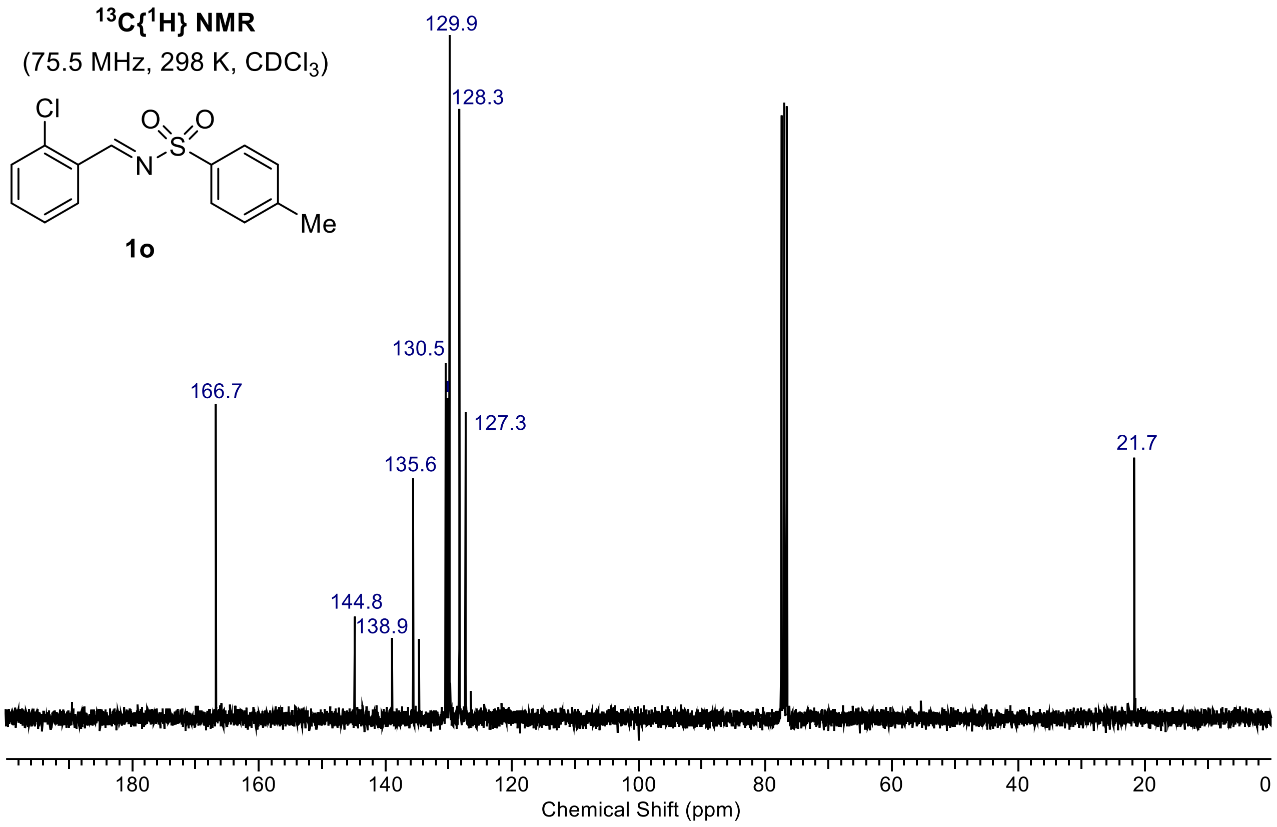

Spectra S21: $^{13}C\{^1H\}$ NMR spectrum of **1o**.

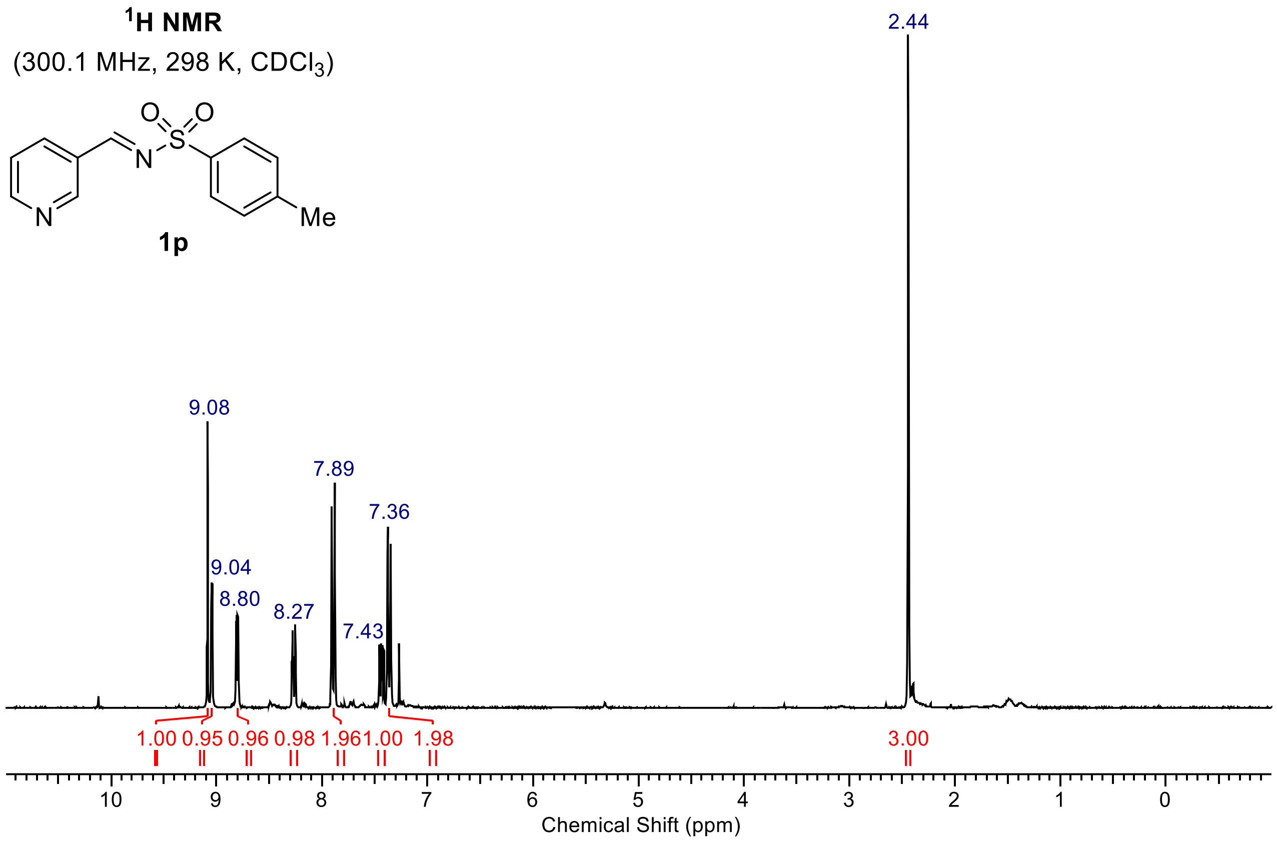

Spectra S22: ^1H NMR spectrum of **1p**.

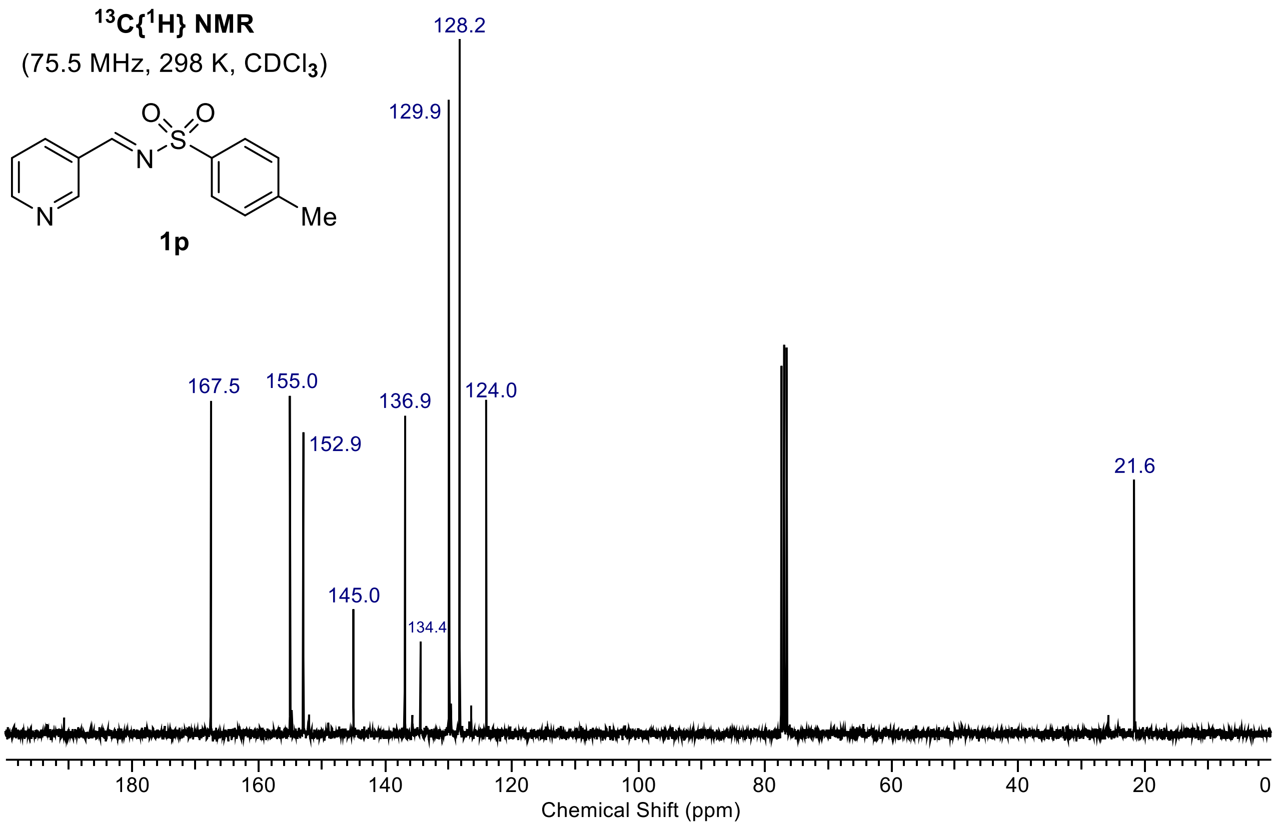

Spectra S23: $^{13}C\{^1H\}$ NMR spectrum of **1q**.

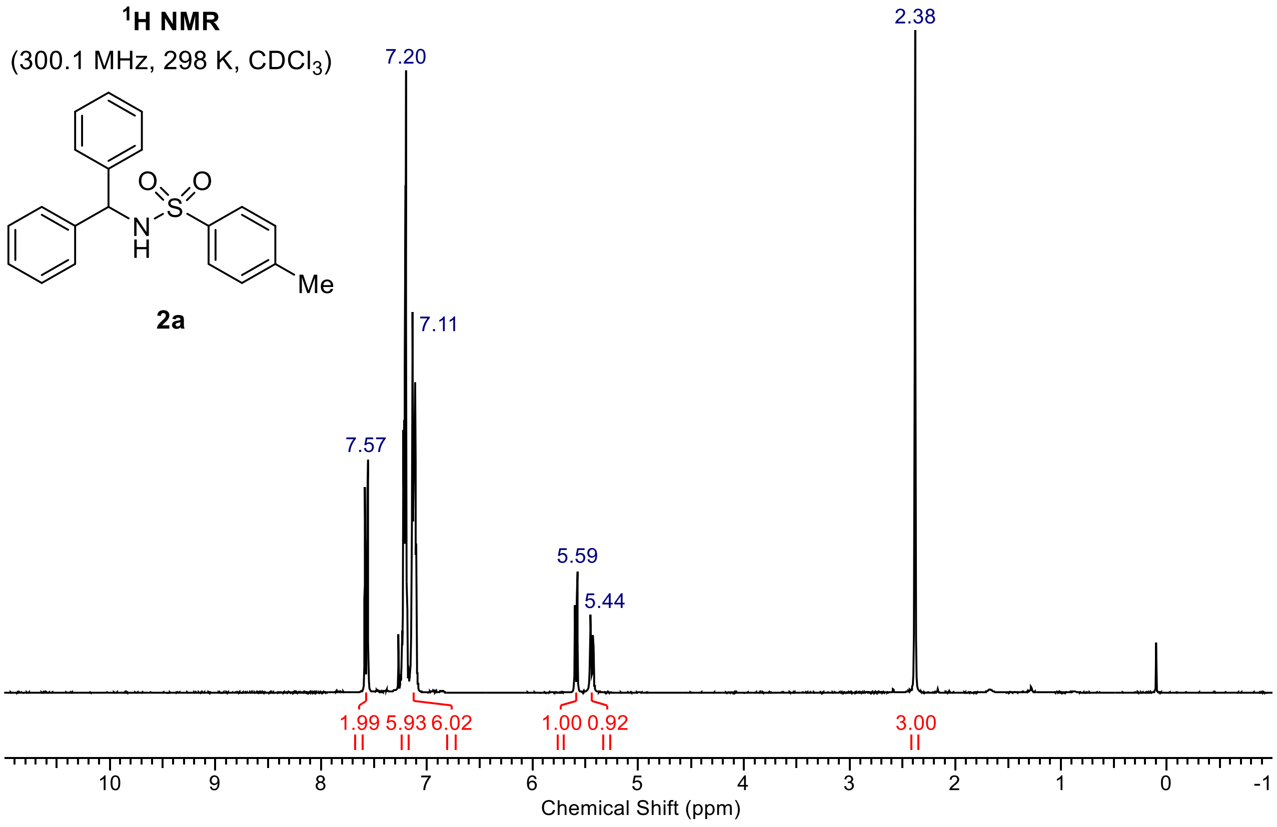

Spectra S24: ^1H NMR spectrum of **2a**.

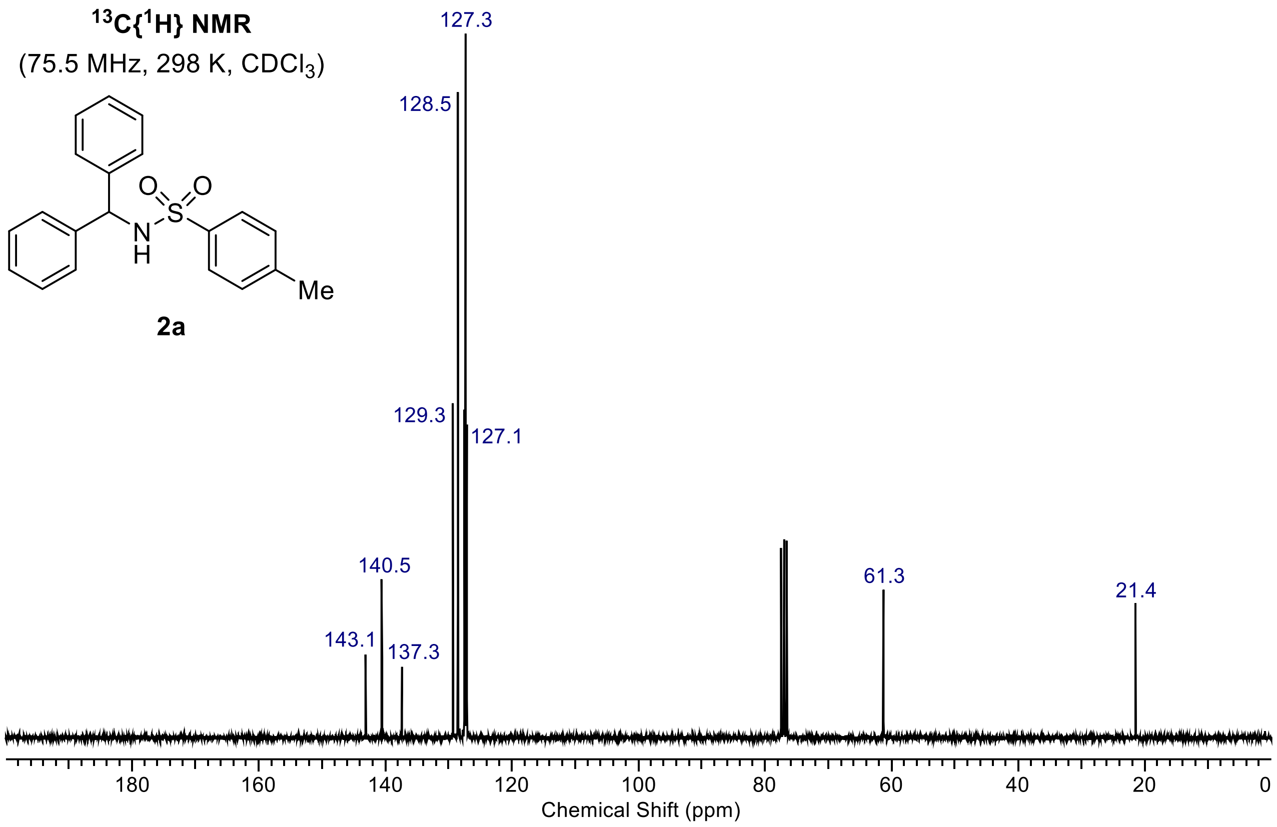

Spectra S25: $^{13}C\{^1H\}$ NMR spectrum of **2a**.

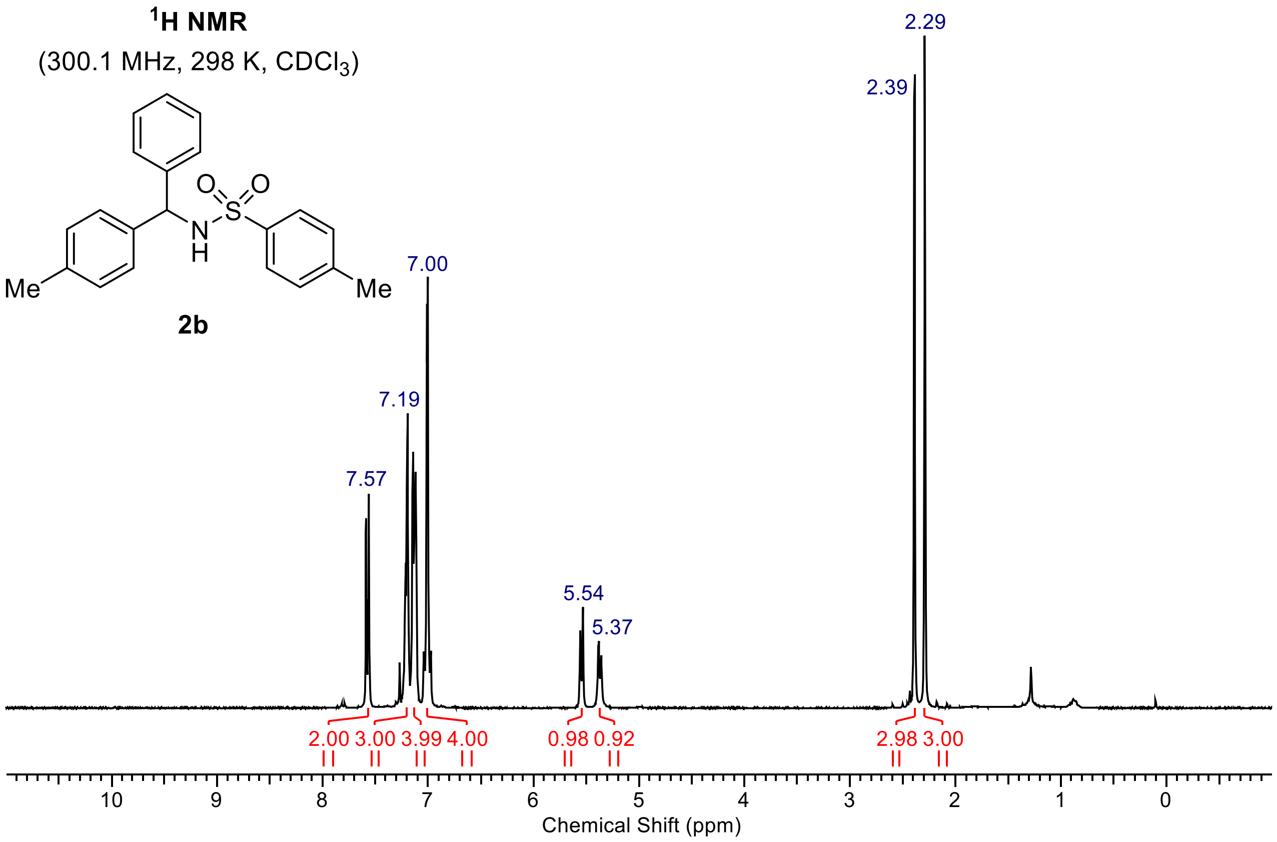

Spectra S26: ^1H NMR spectrum of **2b**.

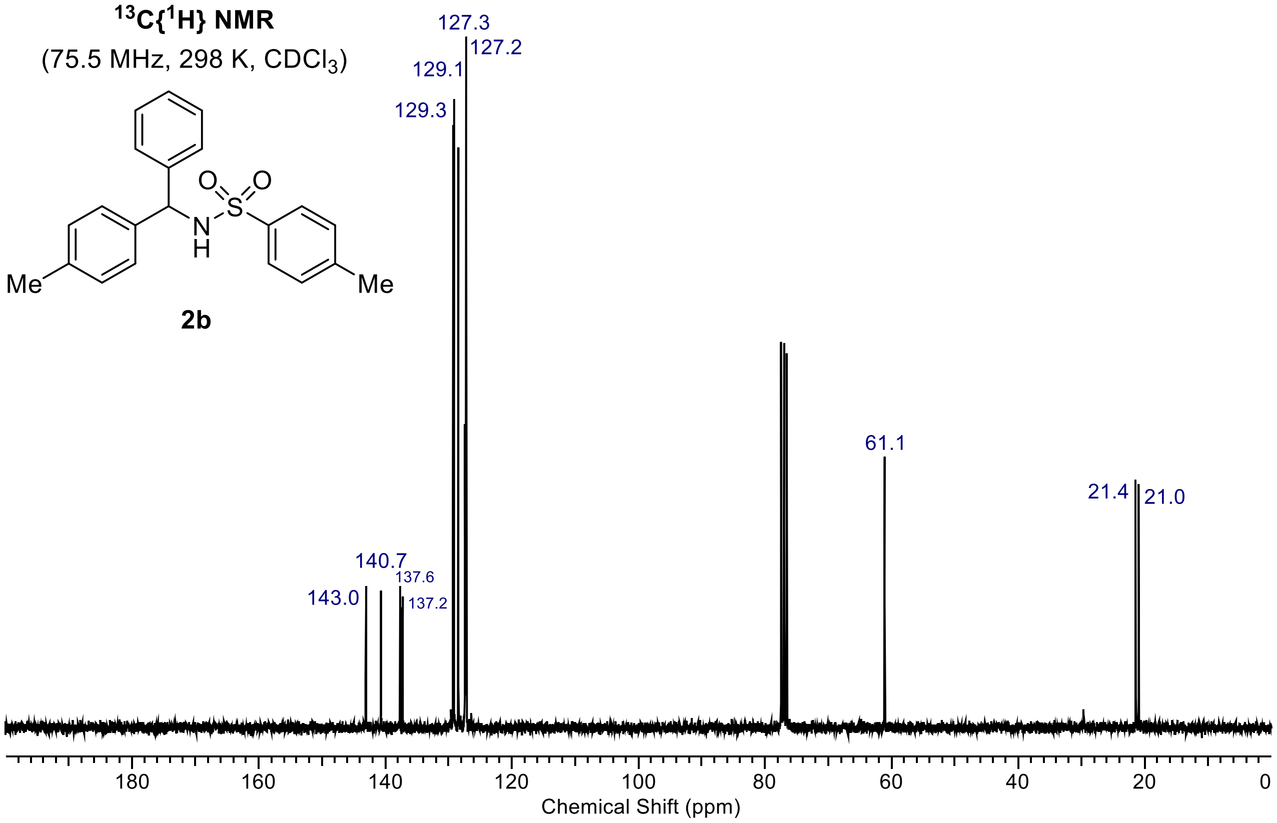

Spectra S27: $^{13}C\{^{1}H\}$ NMR spectrum of **2b**.

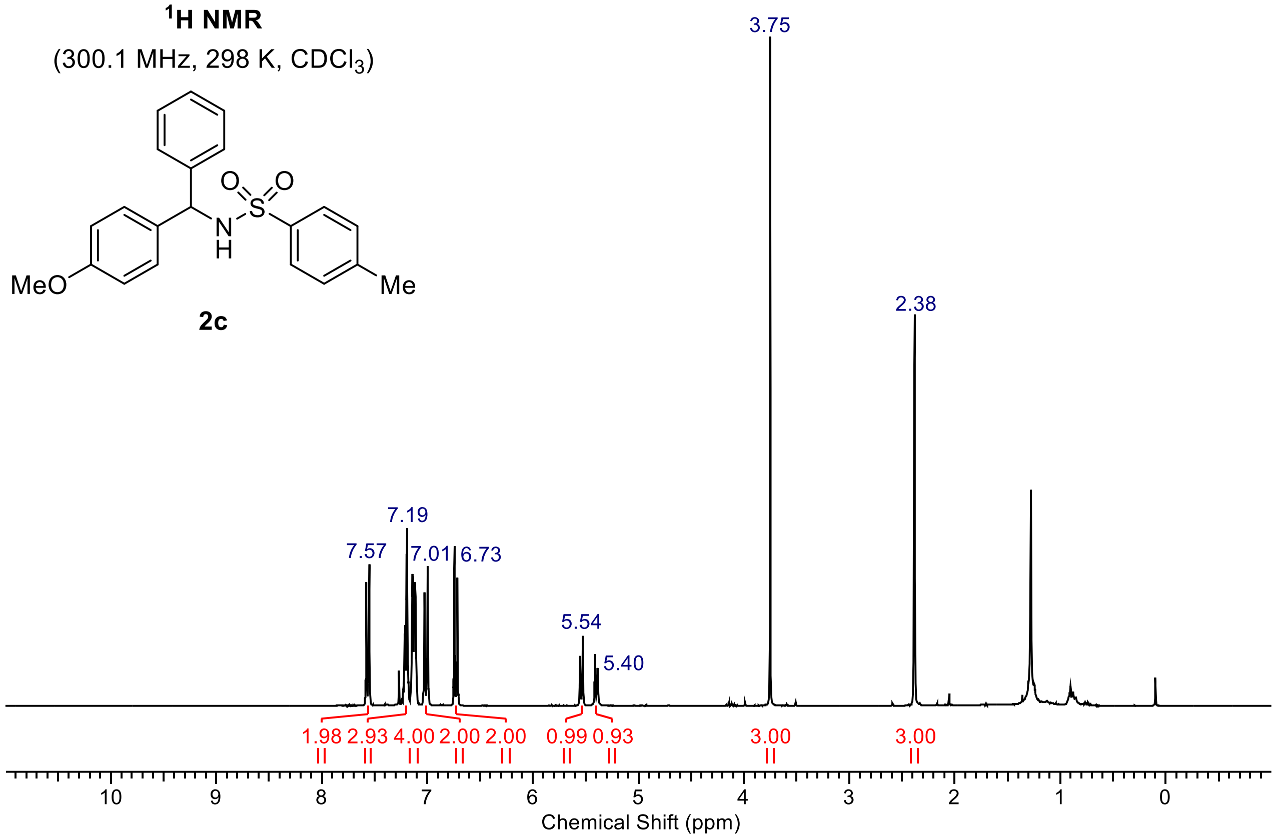

Spectra S28: ^{1}H NMR spectrum of **2c**.

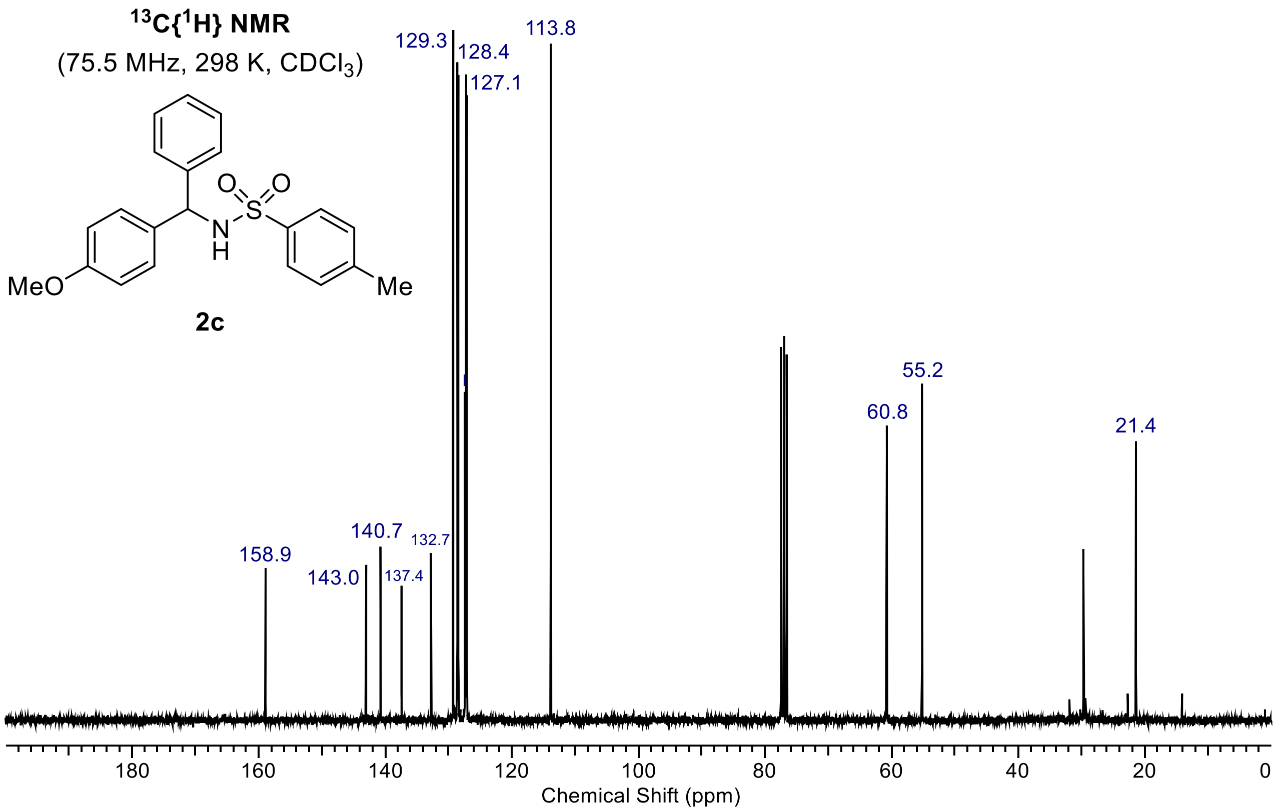

Spectra S29: $^{13}C\{^1H\}$ NMR spectrum of **2c**.

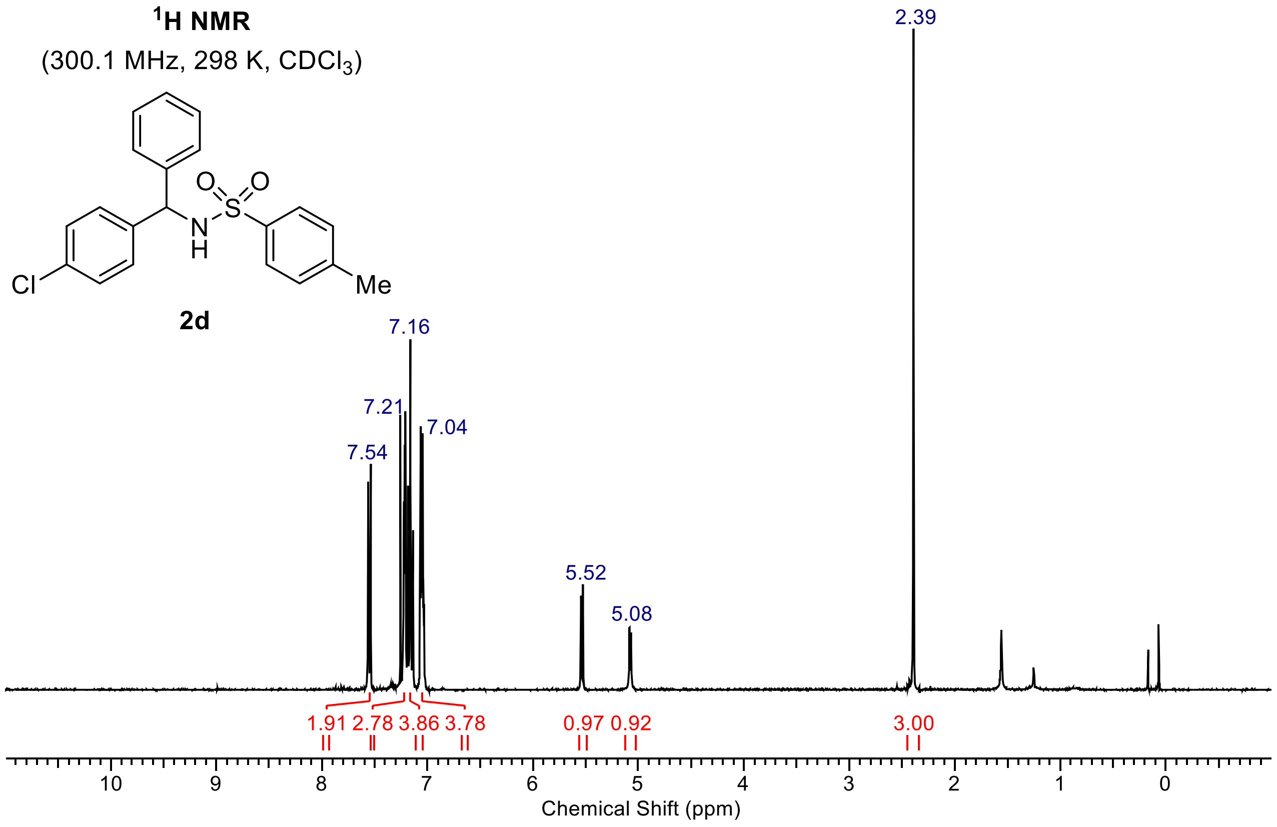

Spectra S30: ^1H NMR spectrum of **2d**.

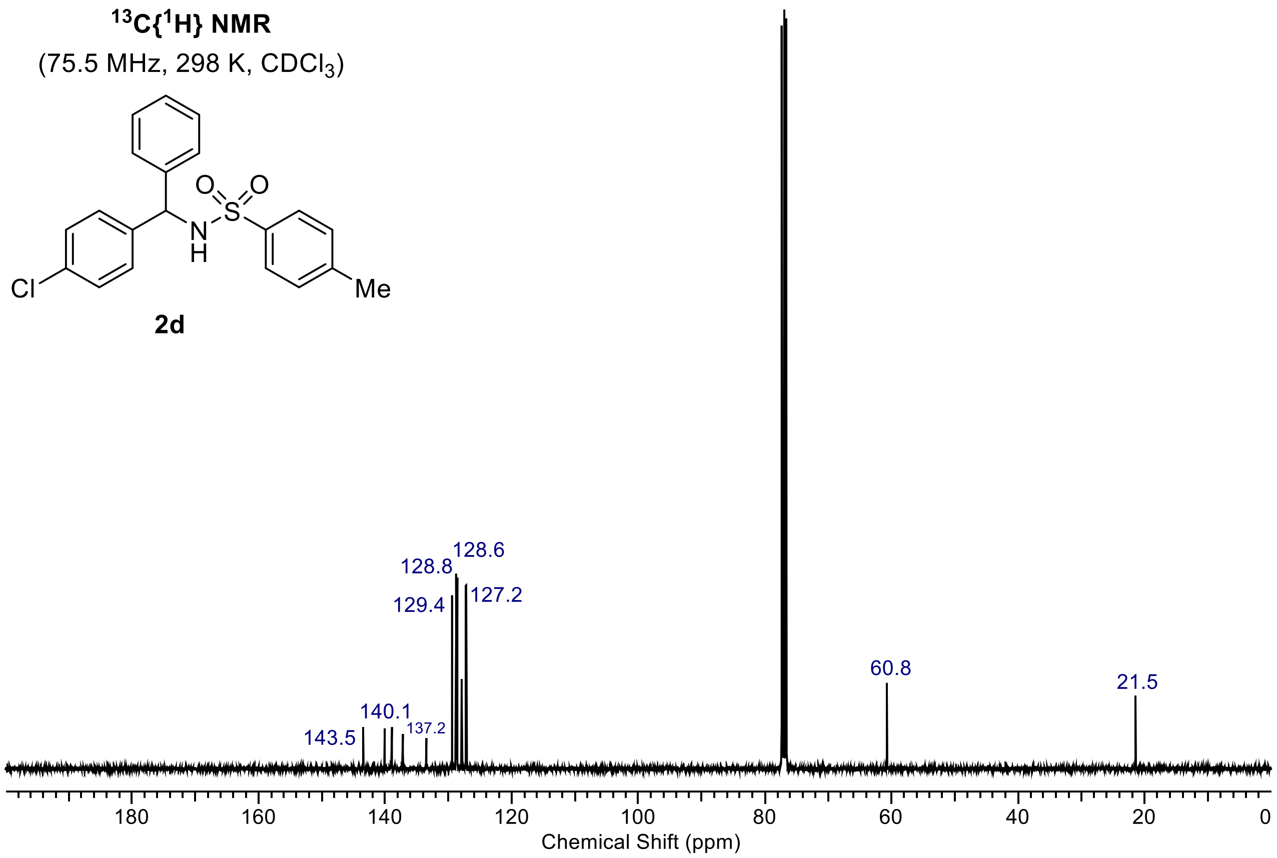

Spectra S31: $^{13}C\{^1H\}$ NMR spectrum of **2d**.

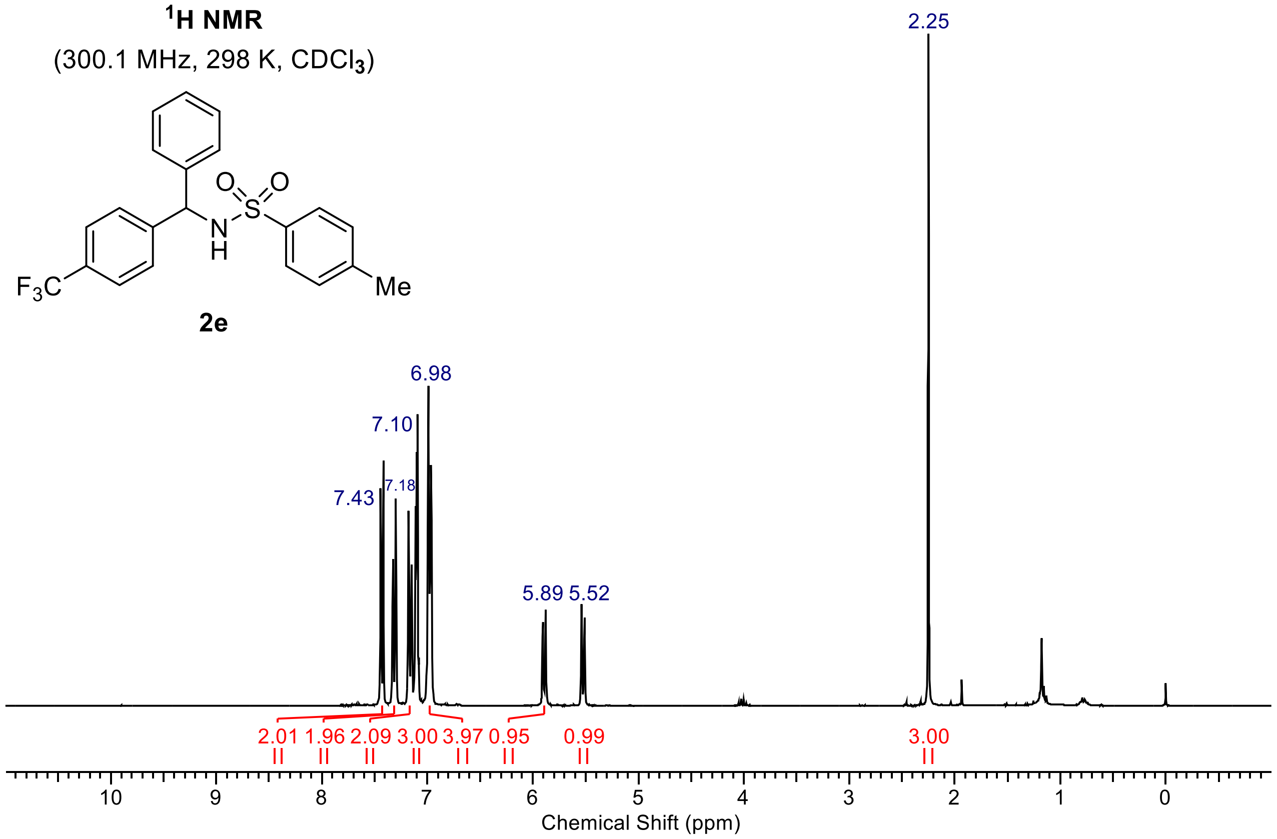

Spectra S32: ^1H NMR spectrum of **2e**.

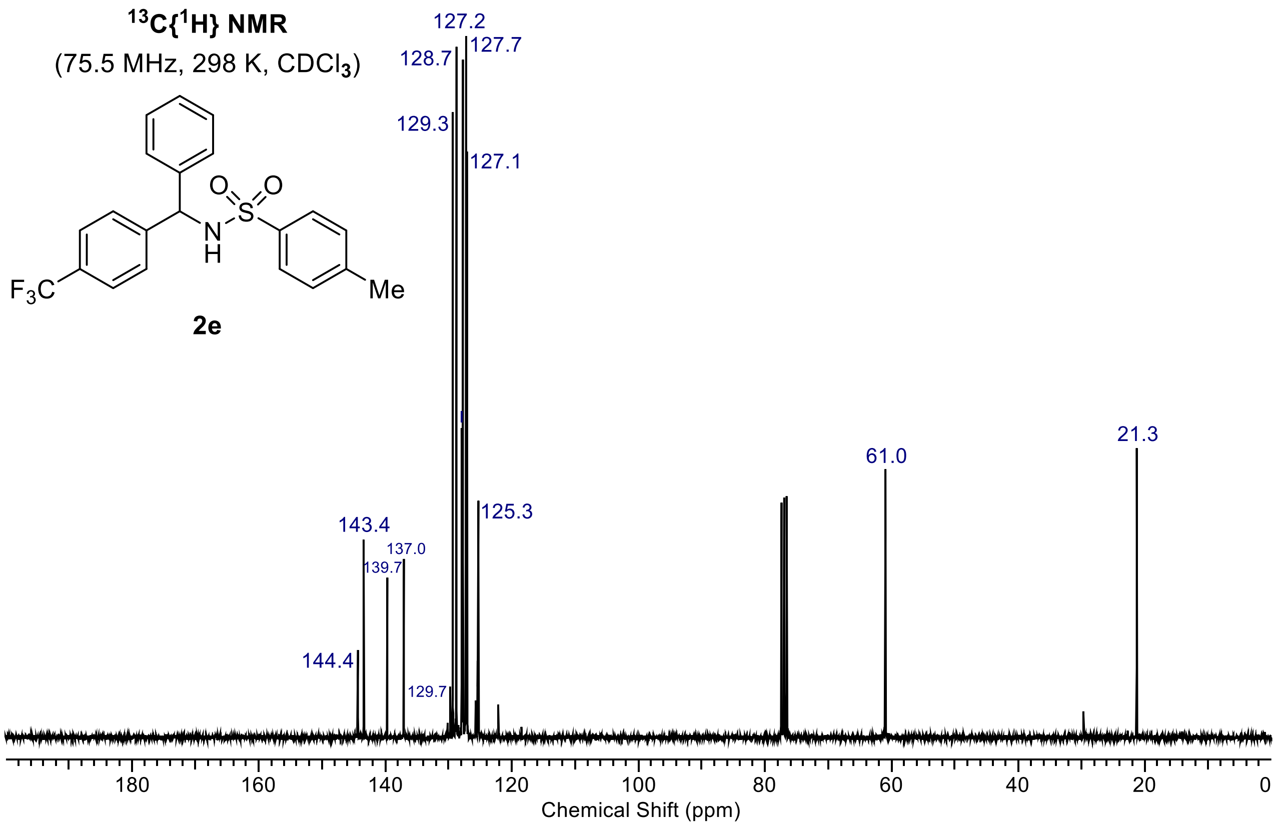

Spectra S33: $^{13}C\{^1H\}$ NMR spectrum of **2e**.

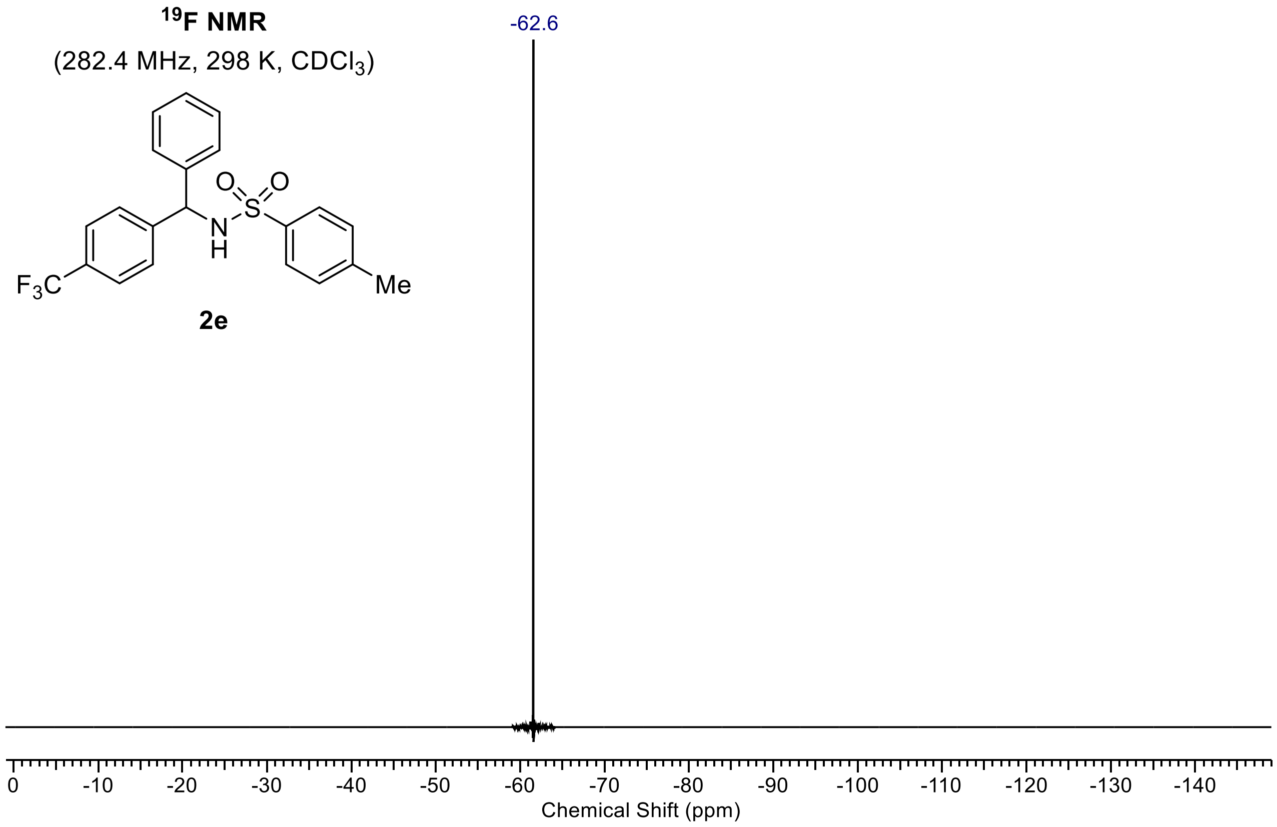

Spectra S34: ^{19}F NMR spectrum of **2e**.

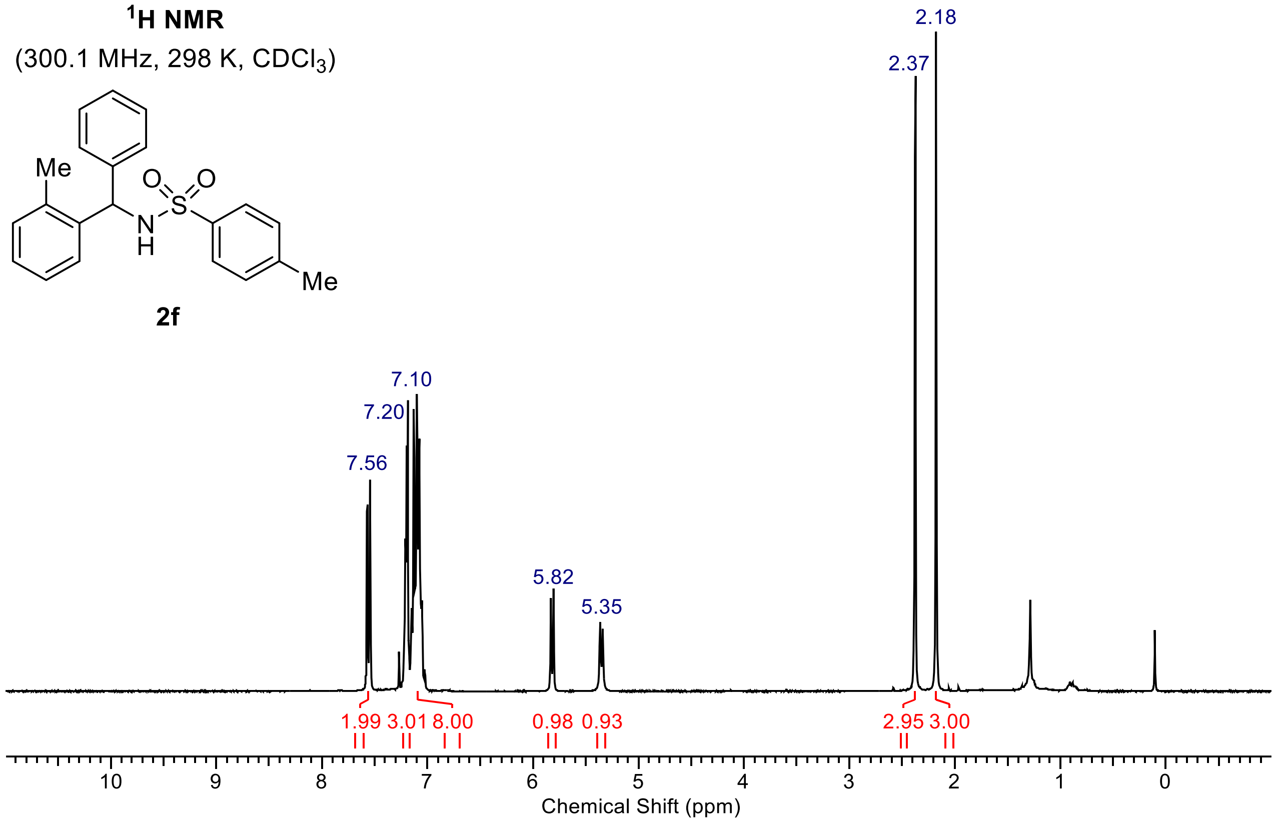

Spectra S35: ^{1}H NMR spectrum of **2f**.

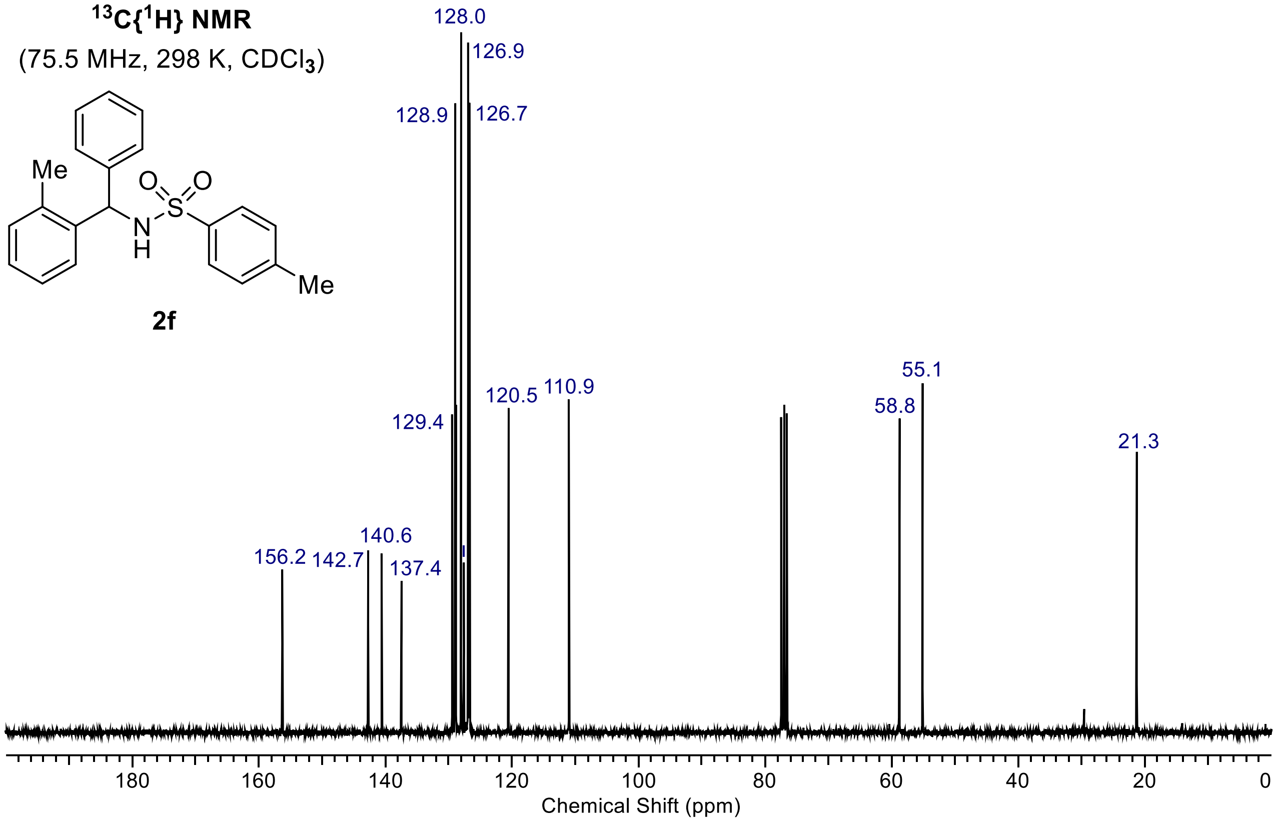

Spectra S36: ^{13}C{^{1}H} NMR spectrum of **2f**.

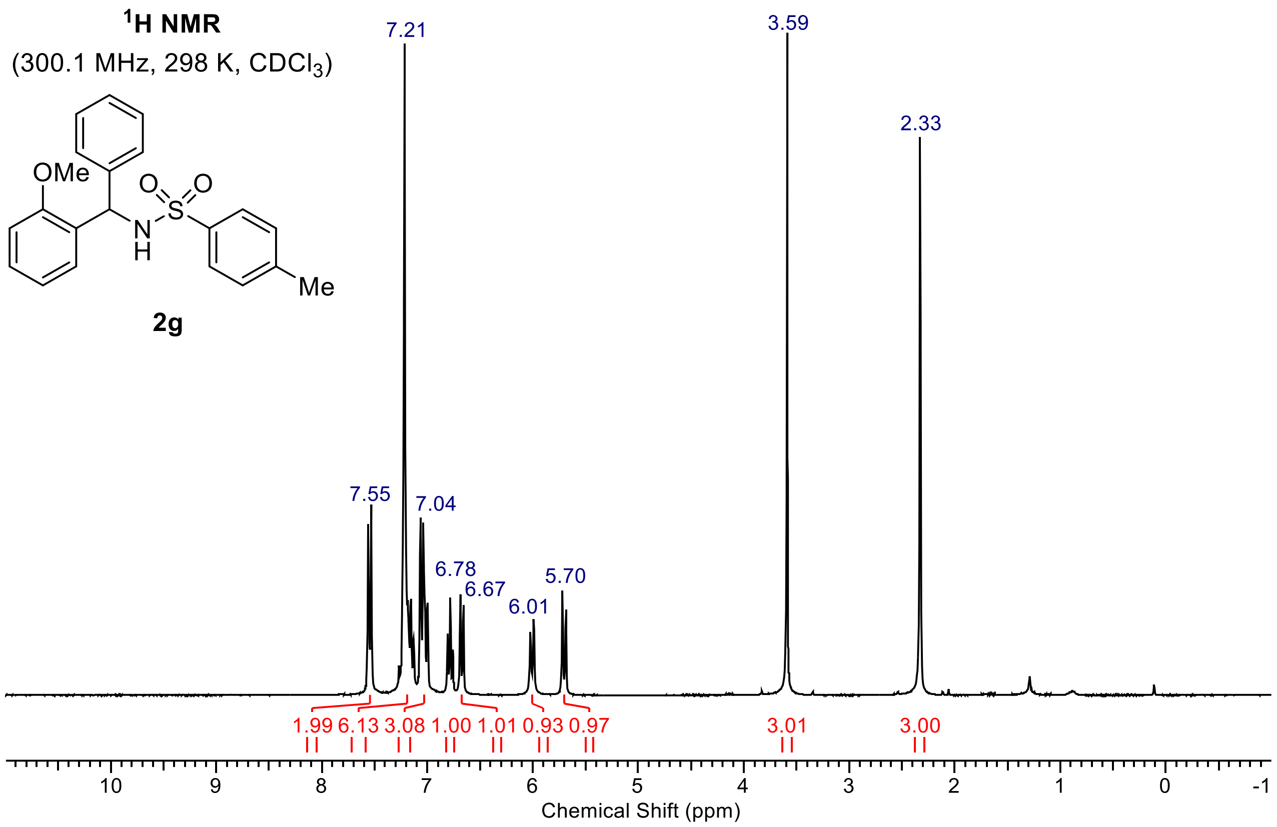

Spectra S37: ^{1}H NMR spectrum of **2g**.

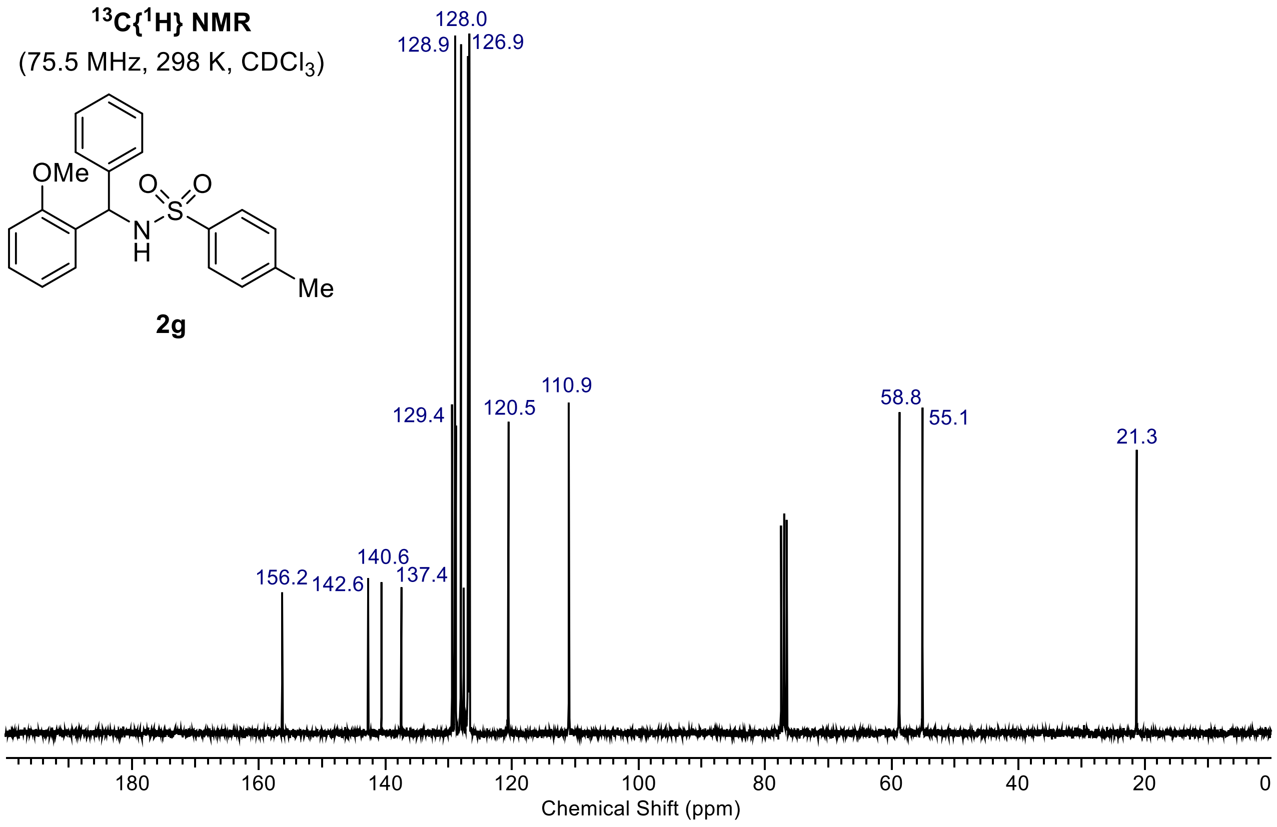

Spectra S38: ^{13}C{^{1}H} NMR spectrum of **2g**.

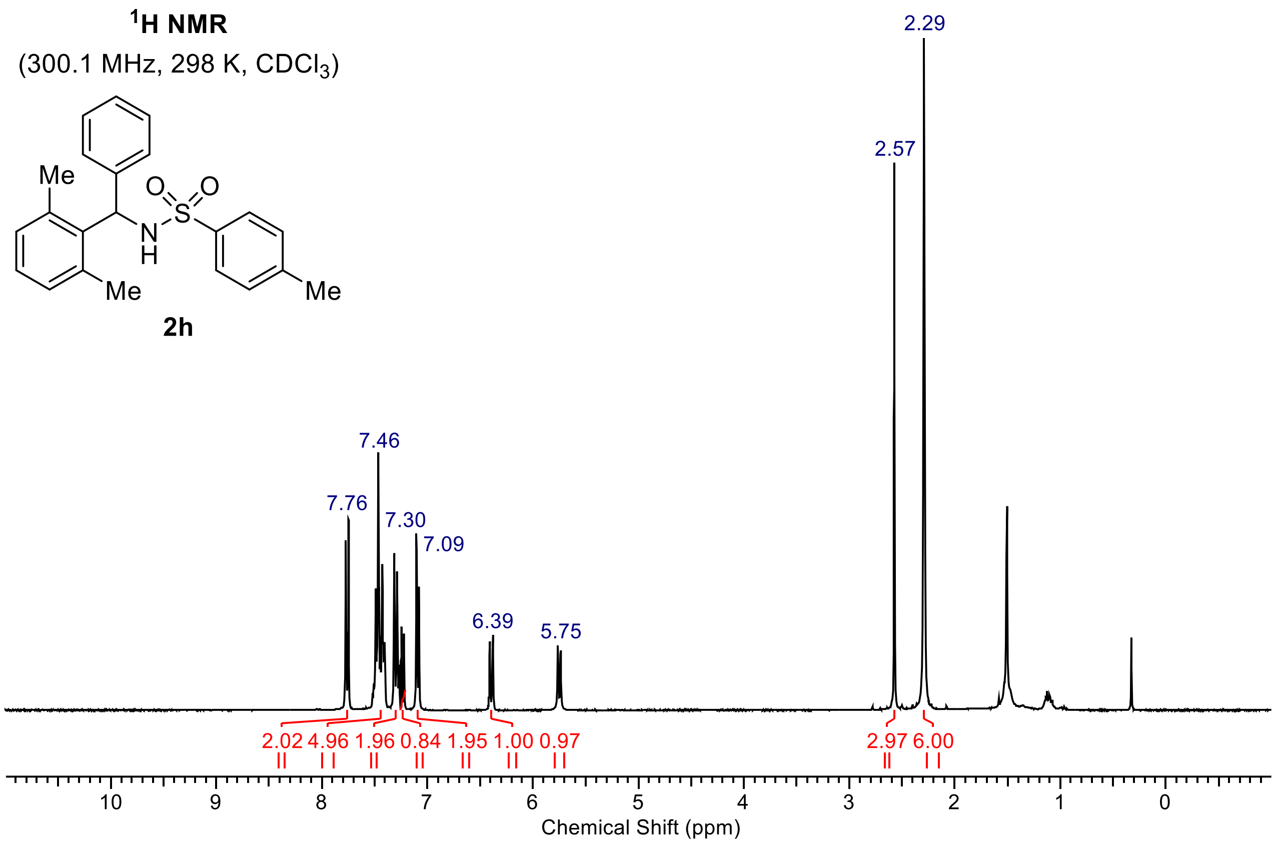

Spectra S39: ^{1}H NMR spectrum of **2h**.

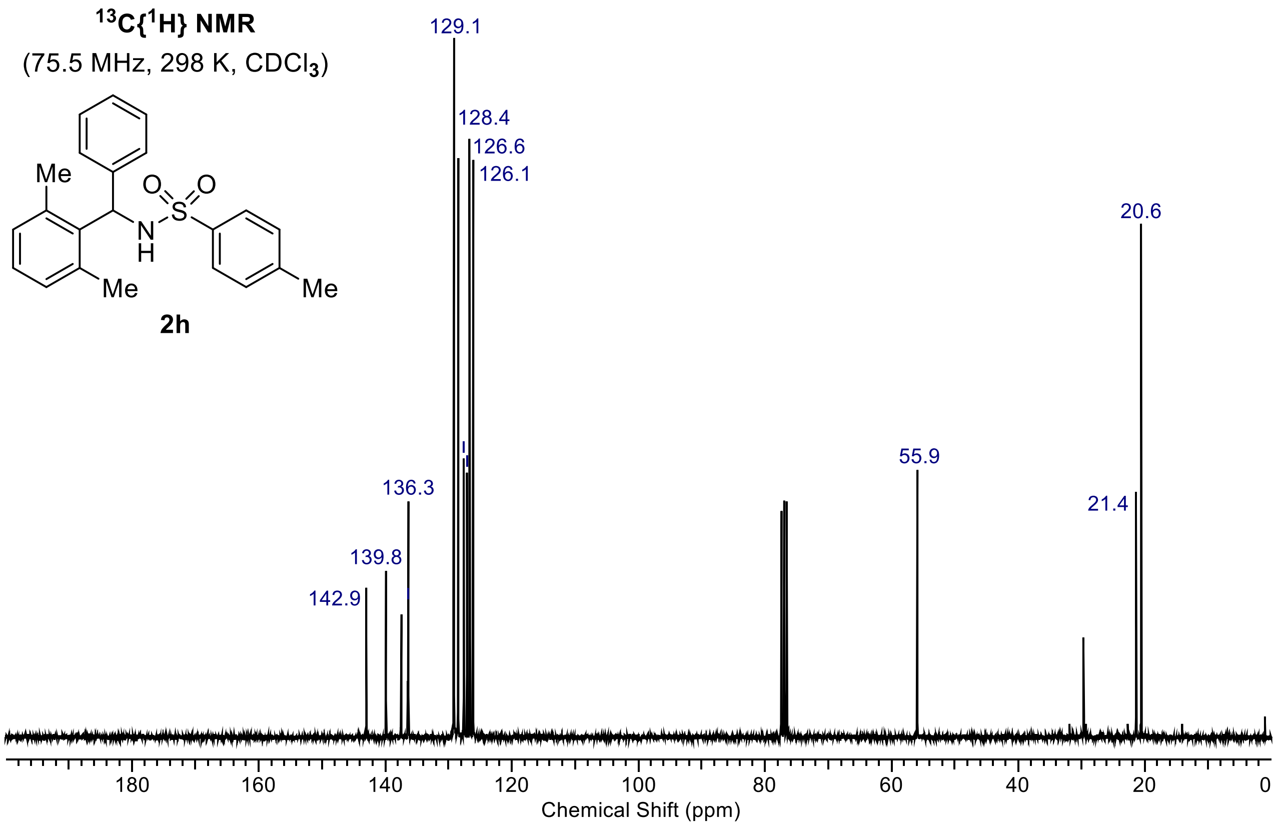

Spectra S40: ^{13}C{^{1}H} NMR spectrum of **2h**.

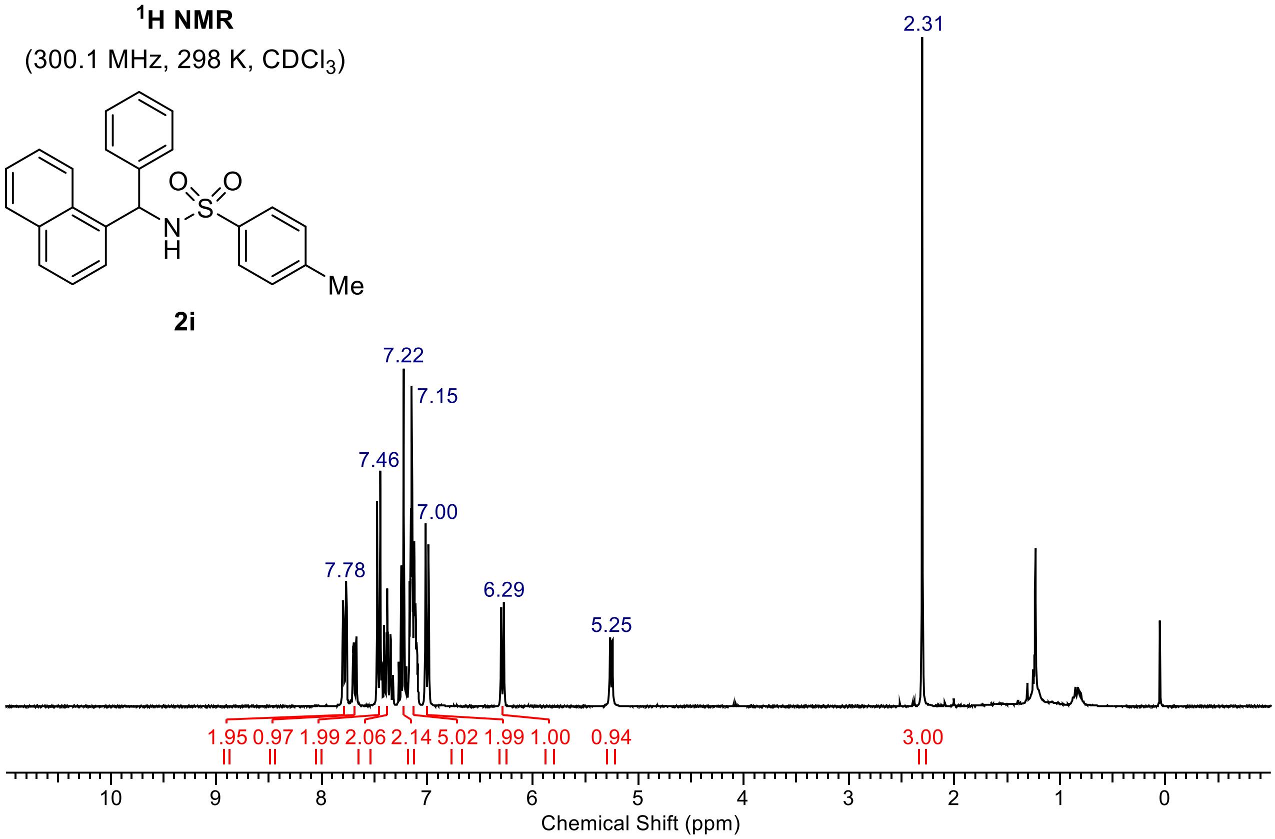

Spectra S41: ^{1}H NMR spectrum of **2i**.

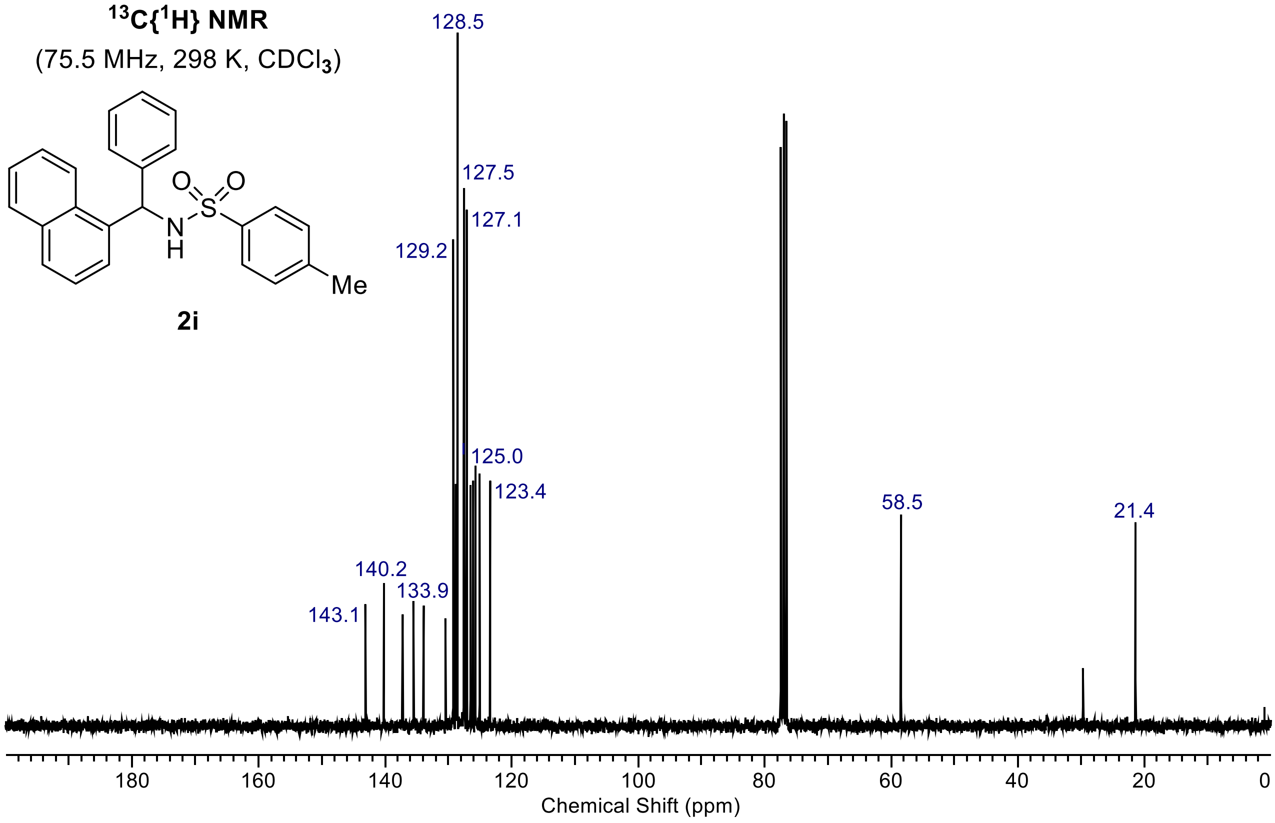

Spectra S42: ^{13}C{^{1}H} NMR spectrum of **2i**.

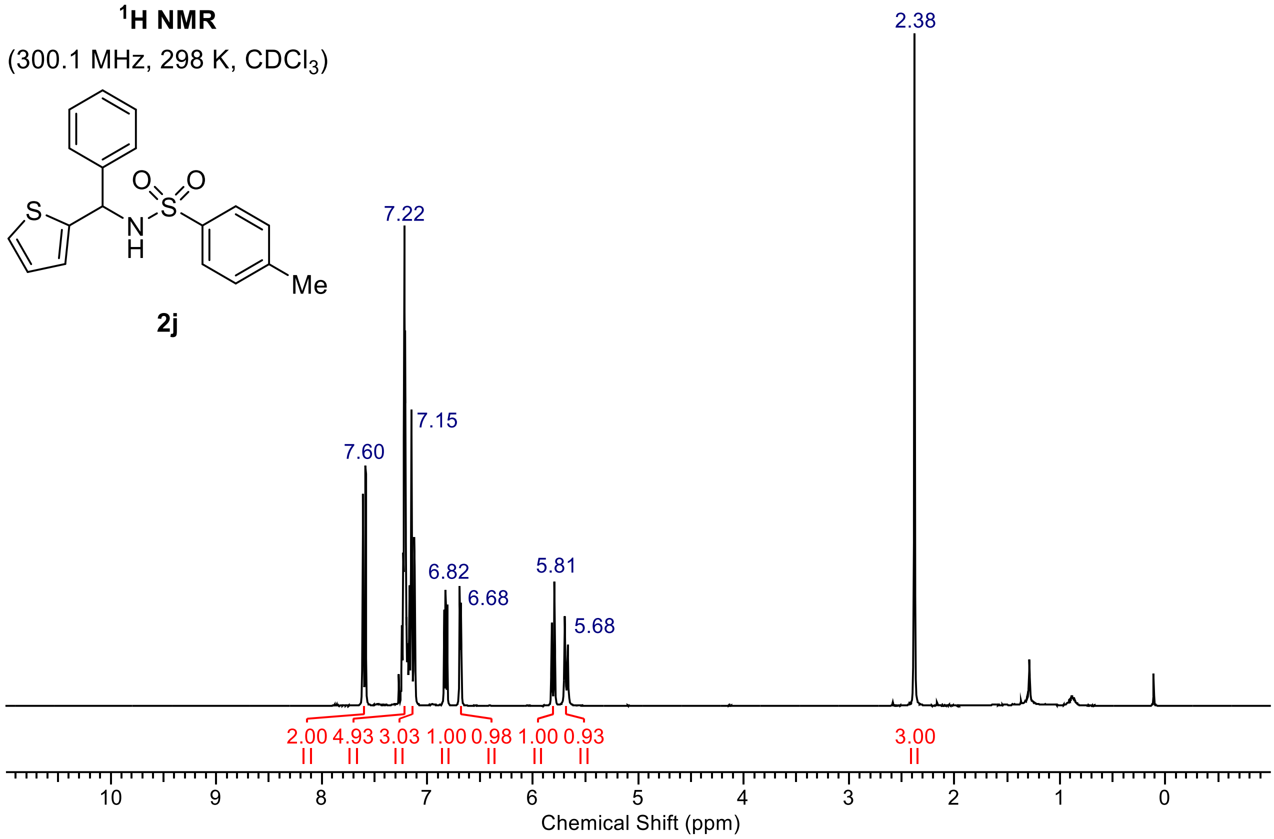

Spectra S43: ^{1}H NMR spectrum of **2j**.

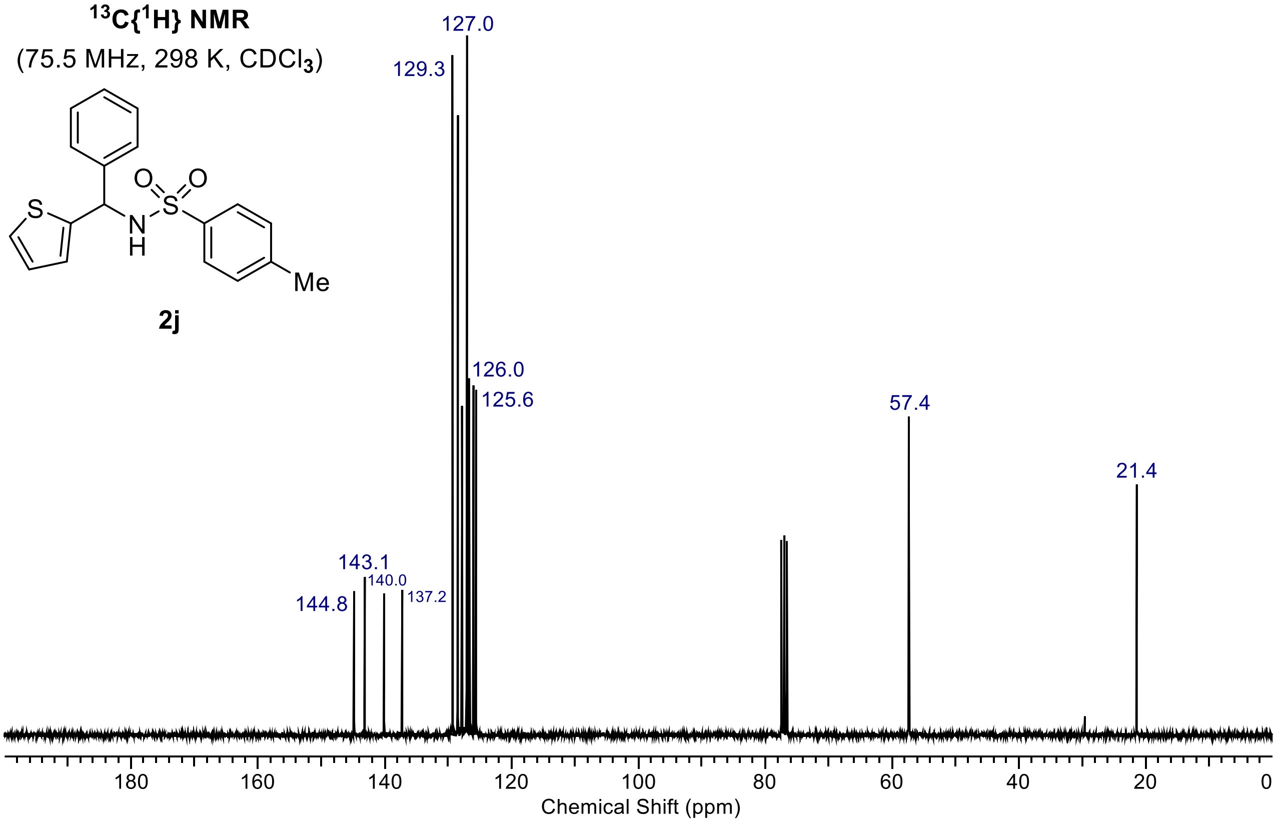

Spectra S44: ^{13}C{^{1}H} NMR spectrum of **2j**.

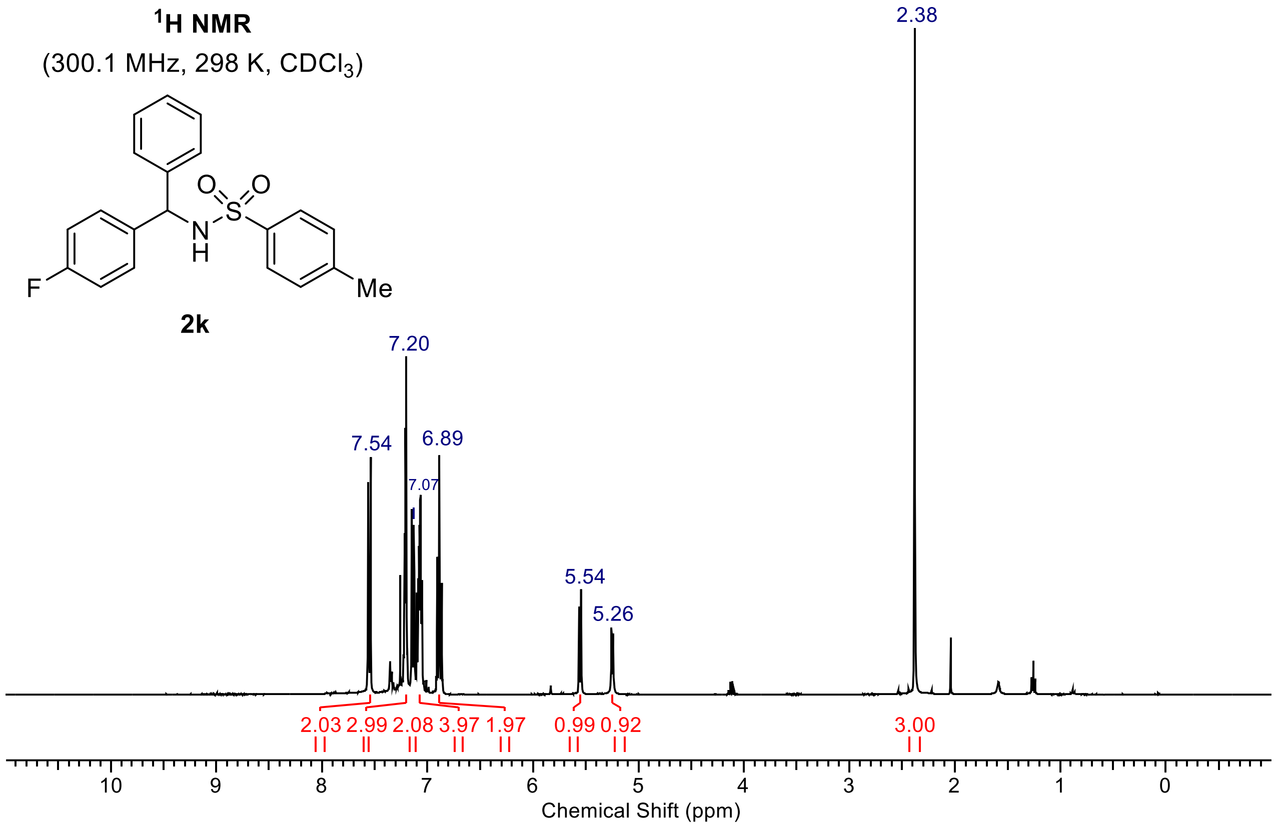

Spectra S45: ^{1}H NMR spectrum of **2k**.

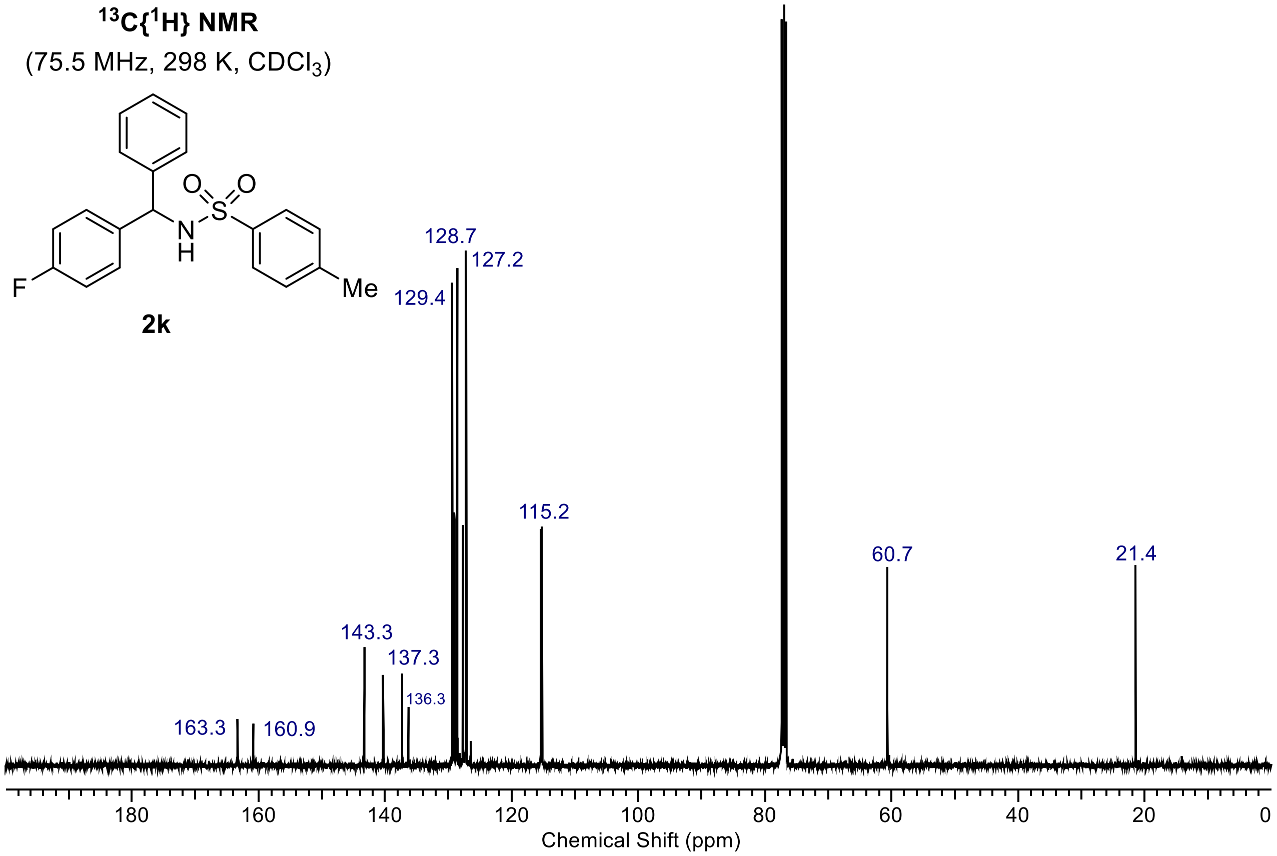

Spectra S46: ^{13}C{^{1}H} NMR spectrum of **2k**.

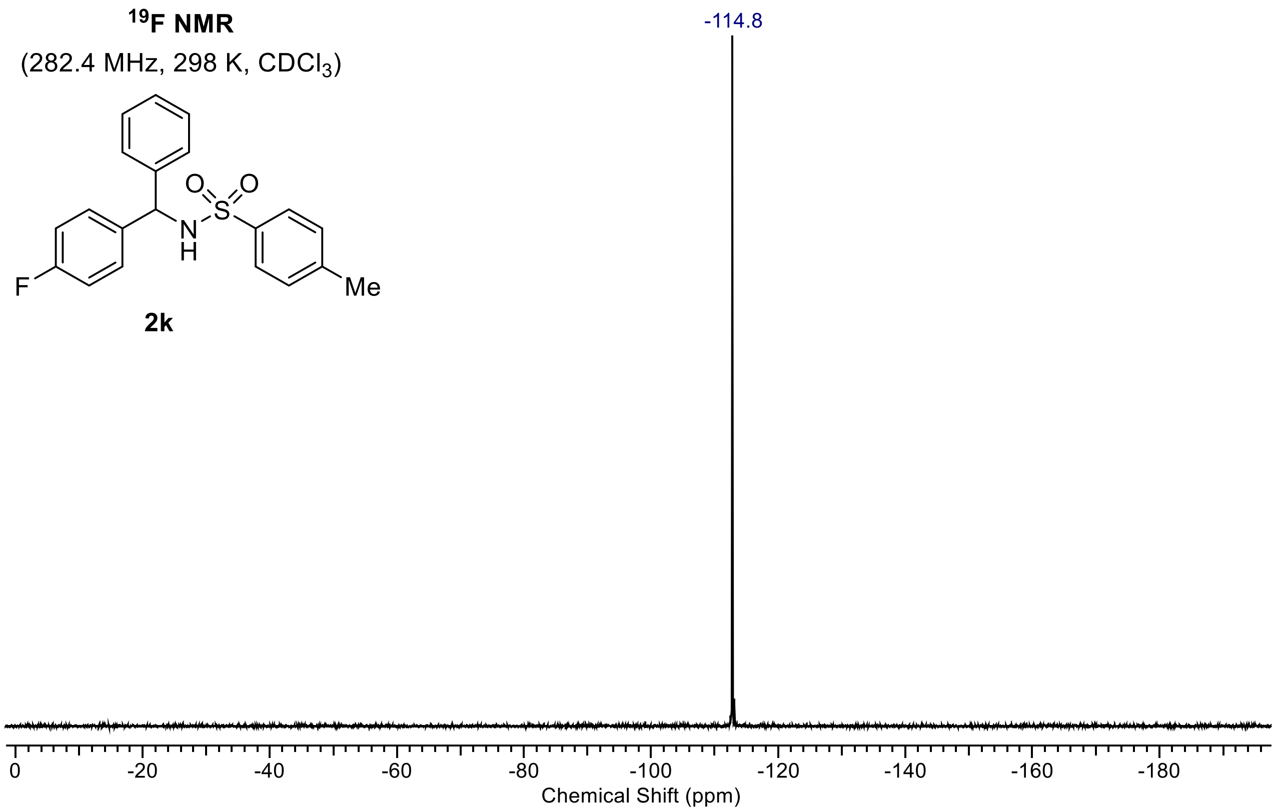

Spectra S47: ^{19}F NMR spectrum of **2k**.

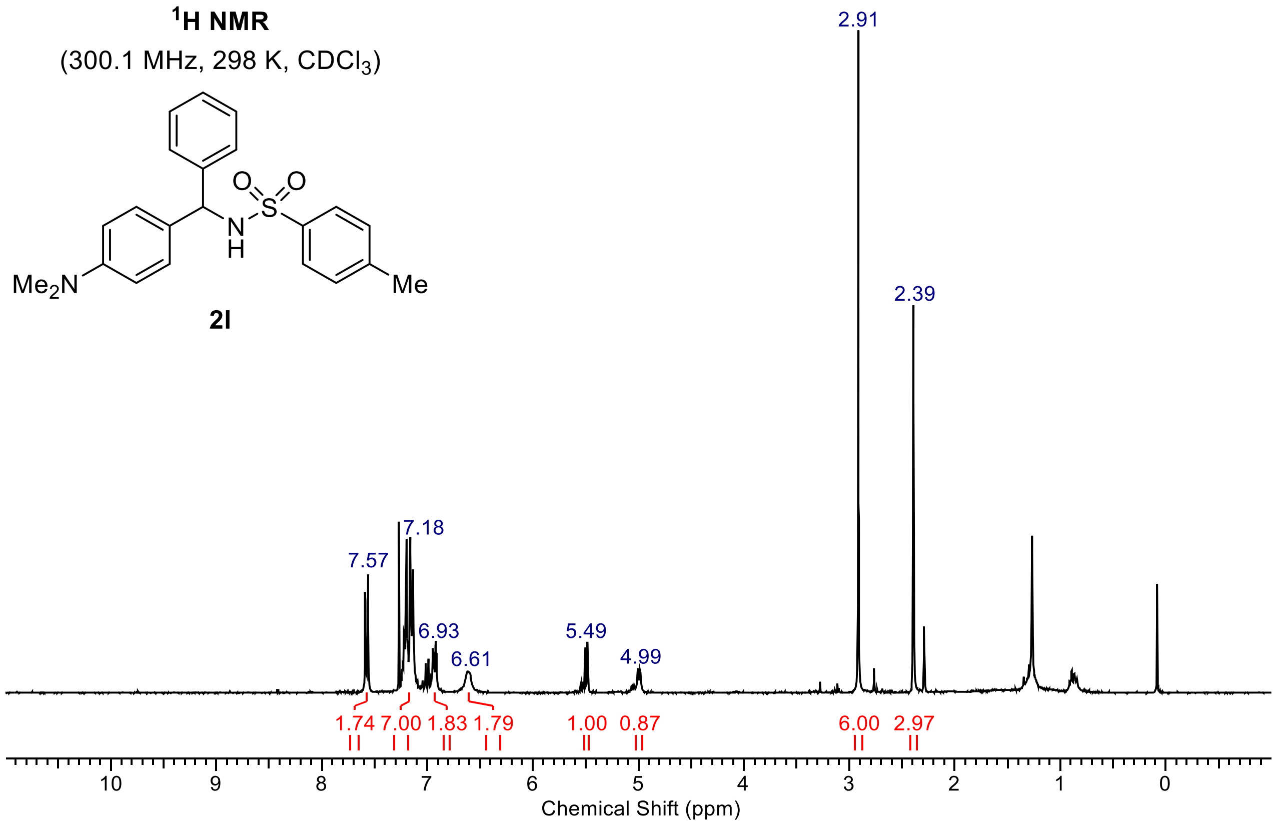

Spectra S48: ^{1}H NMR spectrum of **2l**.

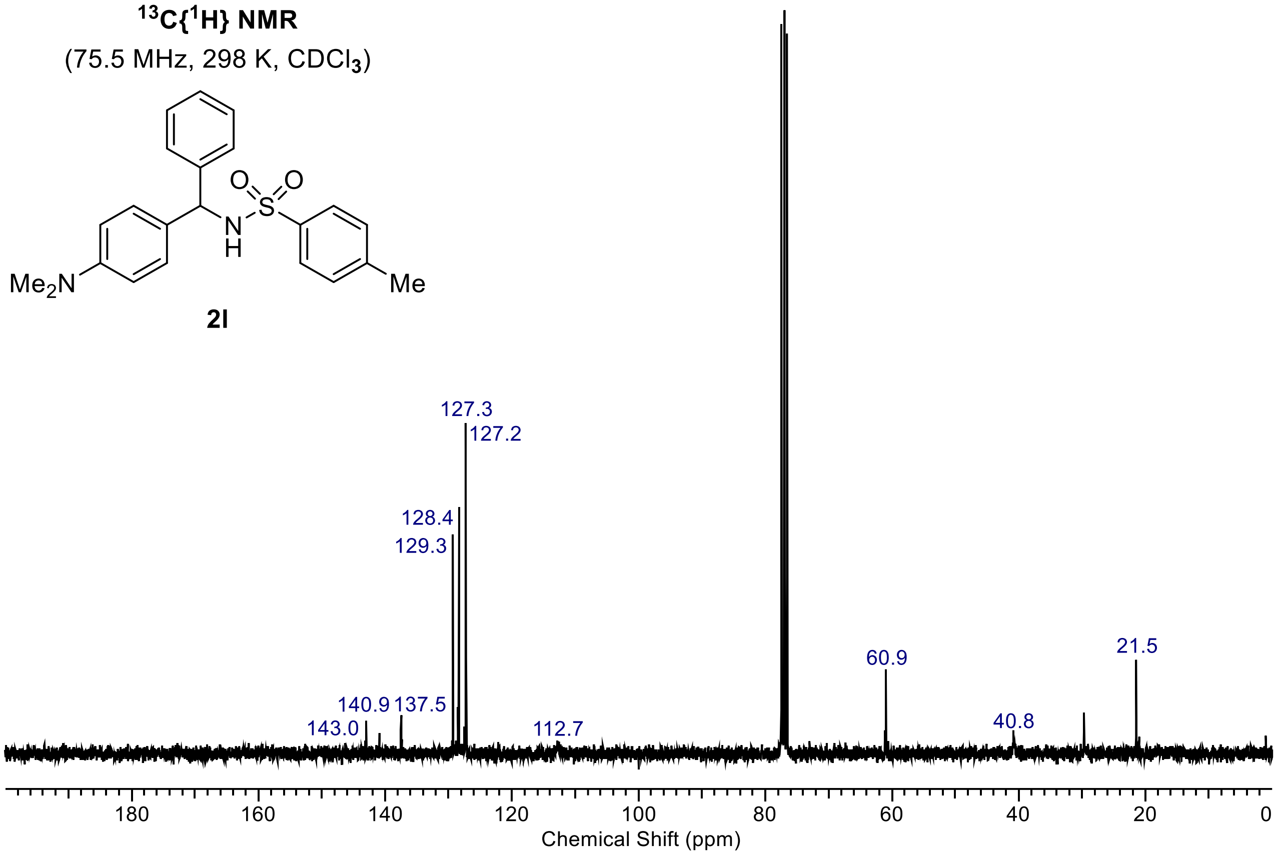

Spectra S49: $^{13}C\{^{1}H\}$ NMR spectrum of **2l**.

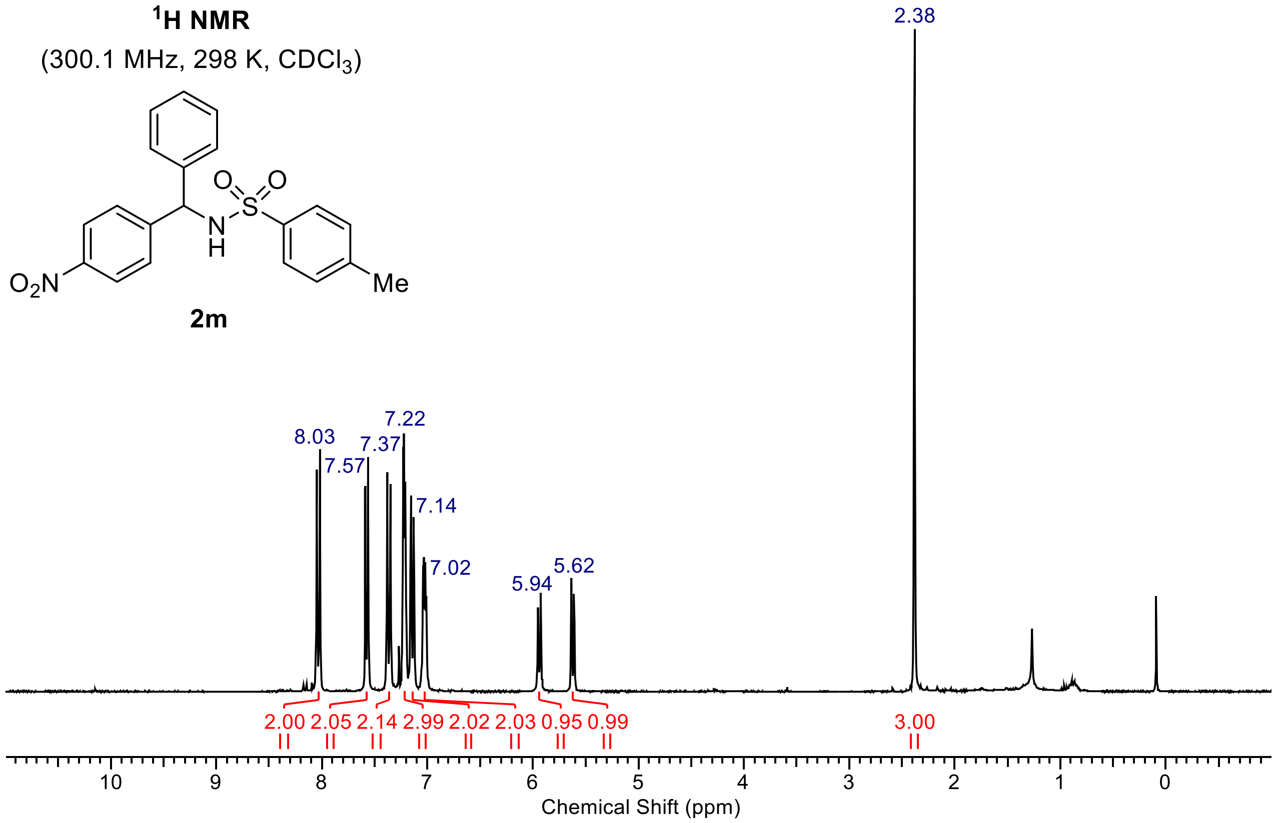

Spectra S50: ^{1}H NMR spectrum of **2m**.

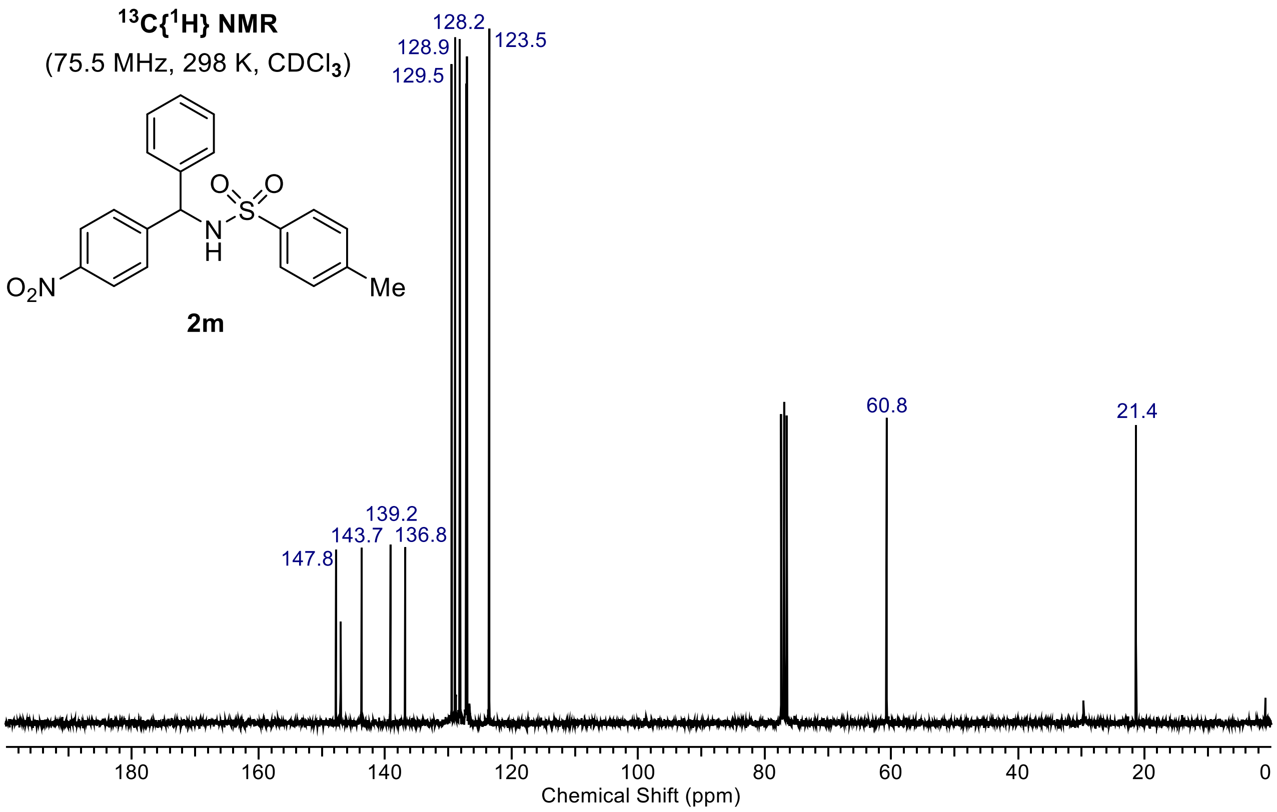

Spectra S51: $^{13}C\{^{1}H\}$ NMR spectrum of **2m**.

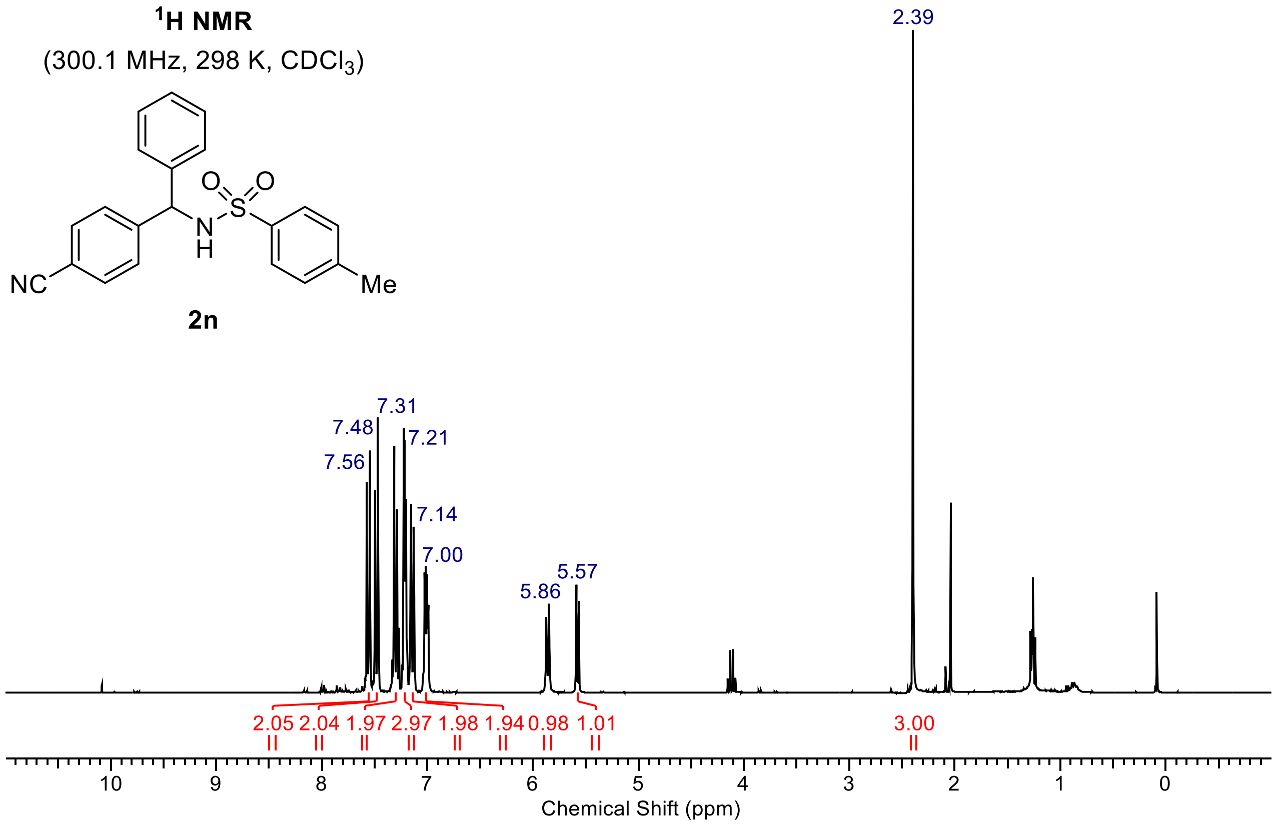

Spectra S52: ^{1}H NMR spectrum of **2n**.

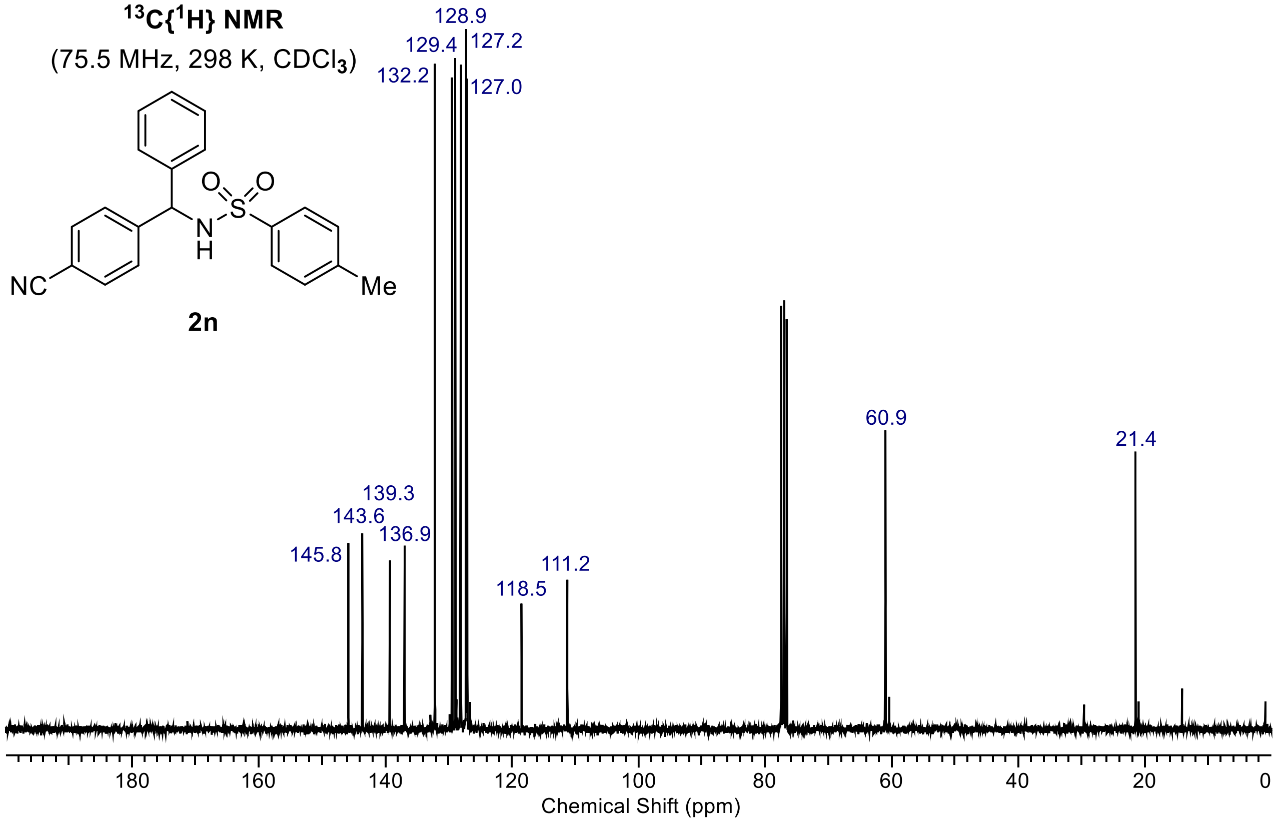

Spectra S53: $^{13}C\{^1H\}$ NMR spectrum of **2n**.

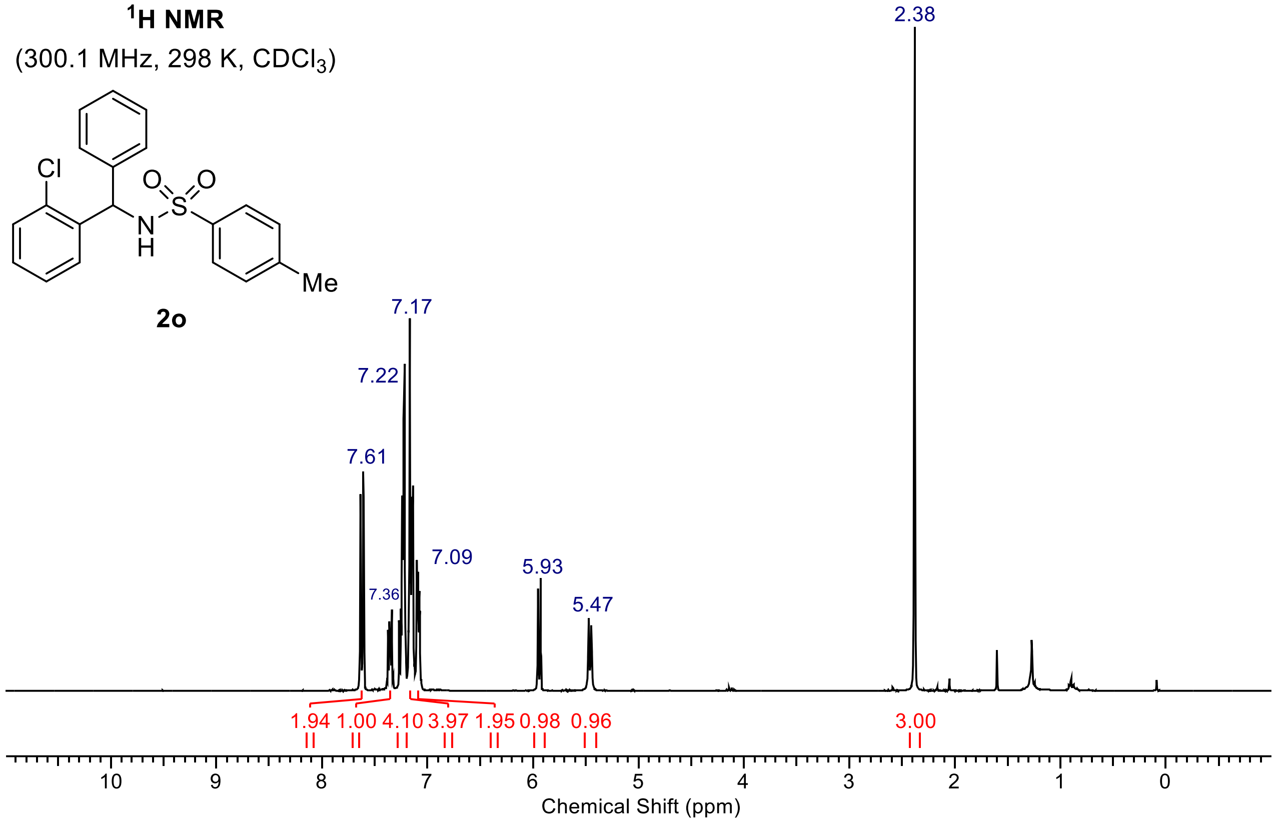

Spectra S54: ^1H NMR spectrum of **2o**.

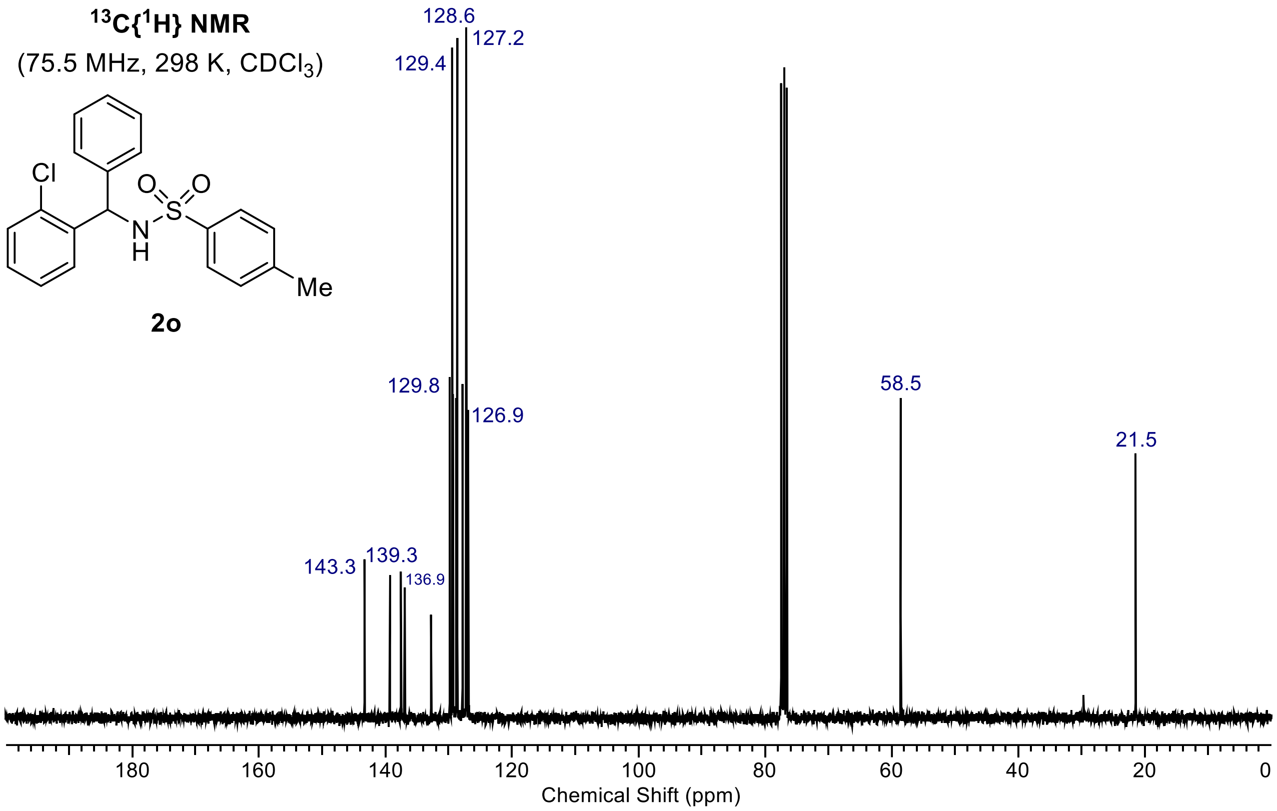

Spectra S55: $^{13}C\{^{1}H\}$ NMR spectrum of **2o**.

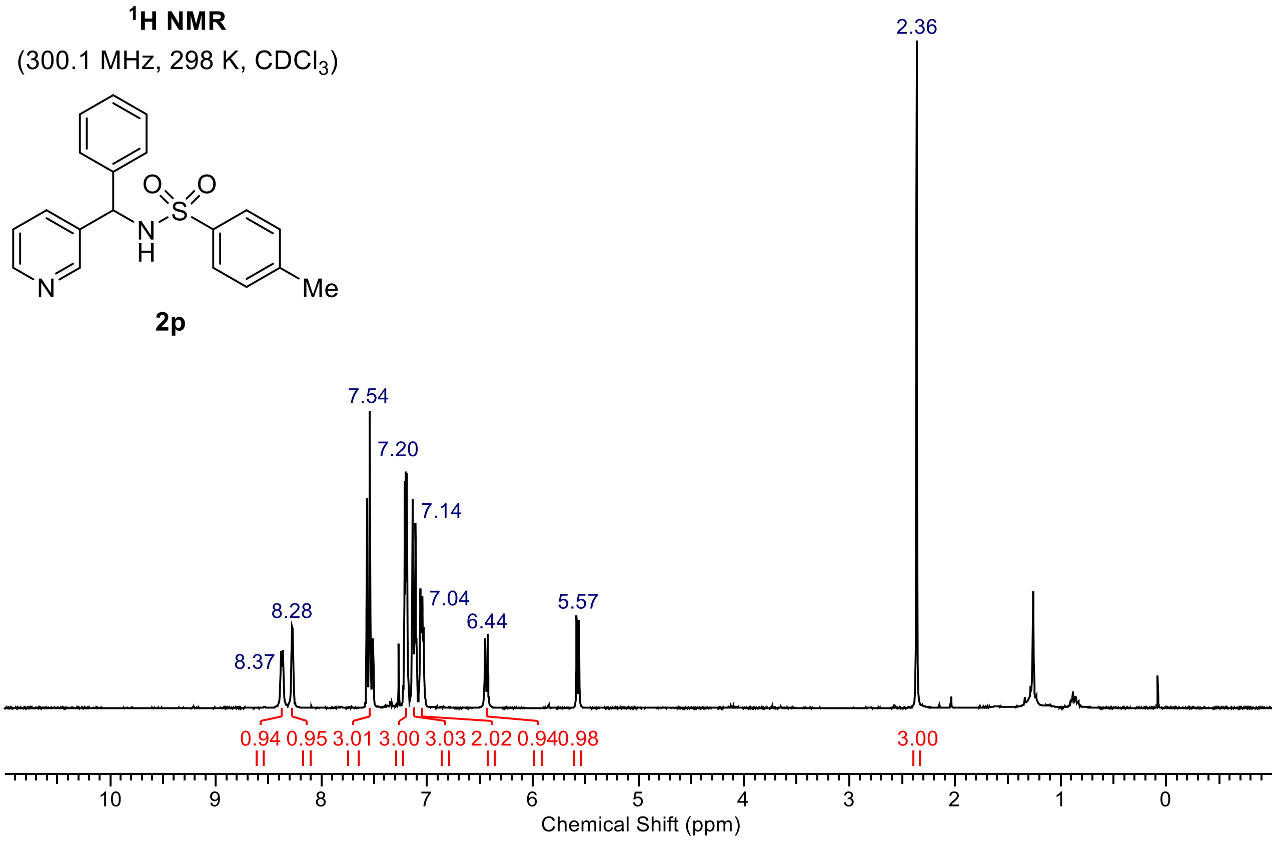

Spectra S56: ^{1}H NMR spectrum of **2p**.

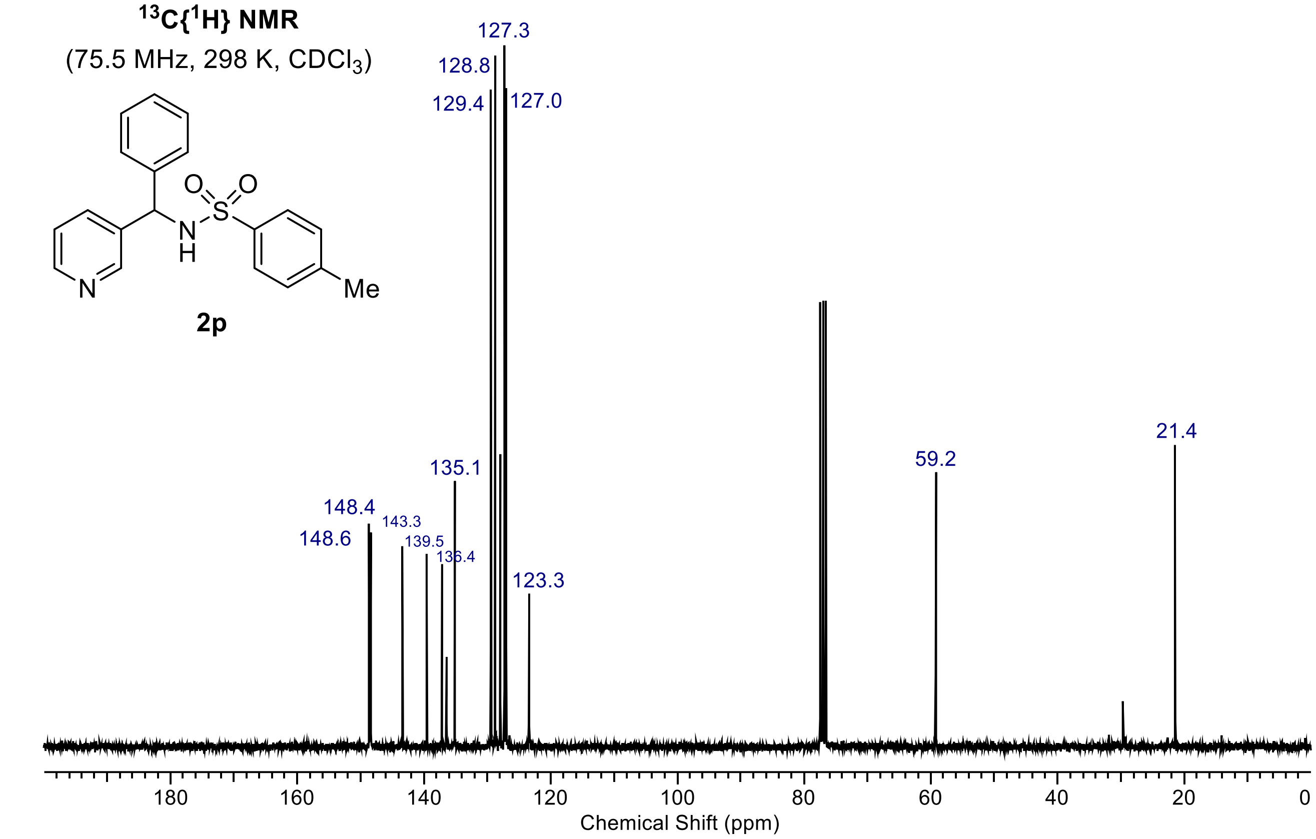

Spectra S57: $^{13}C\{^{1}H\}$ NMR spectrum of **2p**.

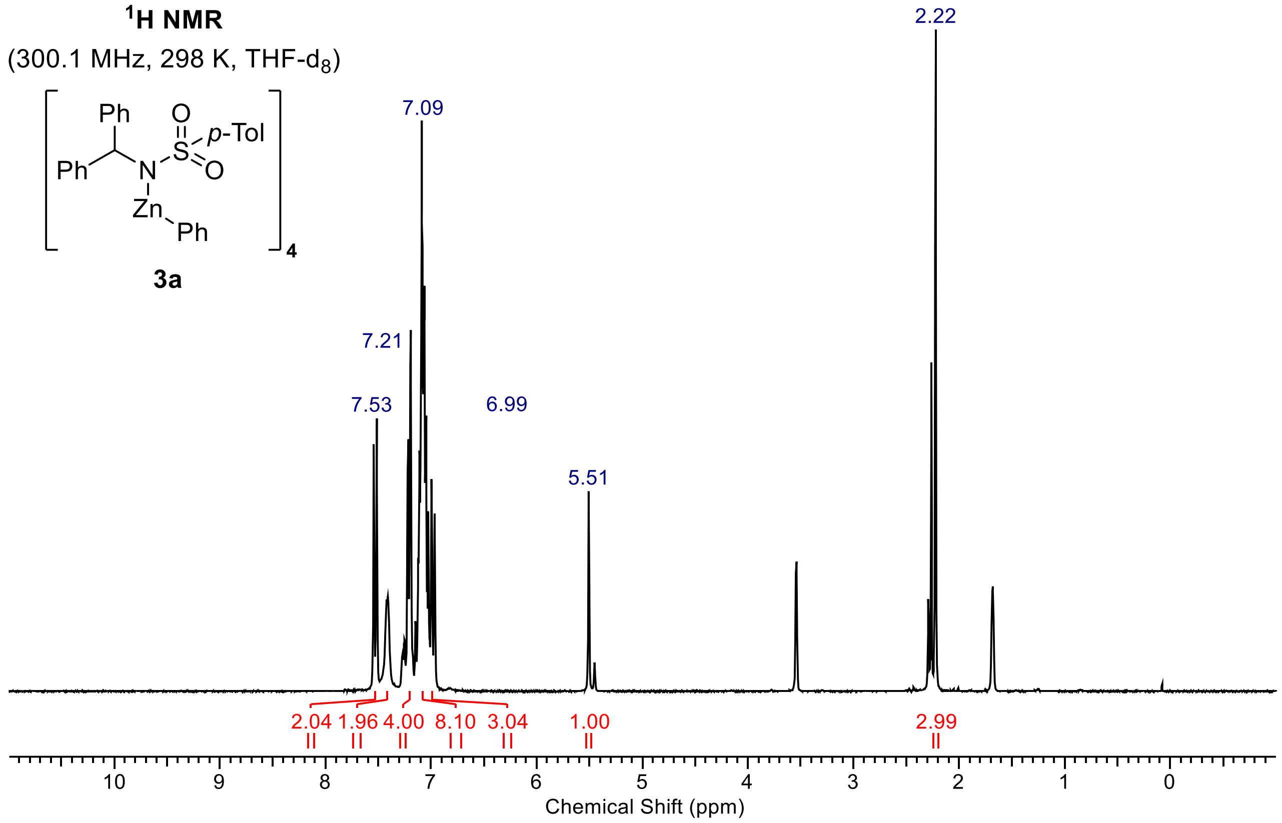

Spectra S58: ^{1}H NMR spectrum of **3a**.

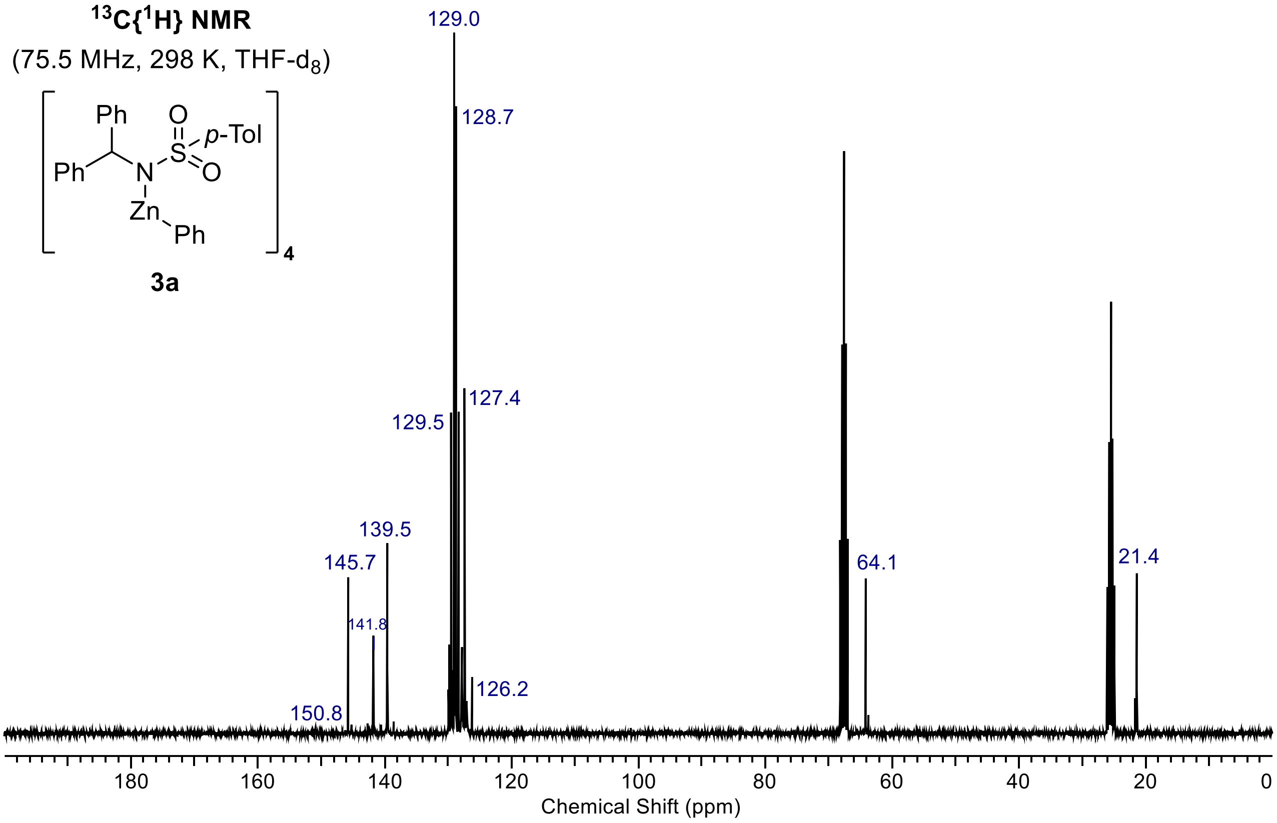

Spectra S59: $^{13}C\{^{1}H\}$ NMR spectrum of **3a**.

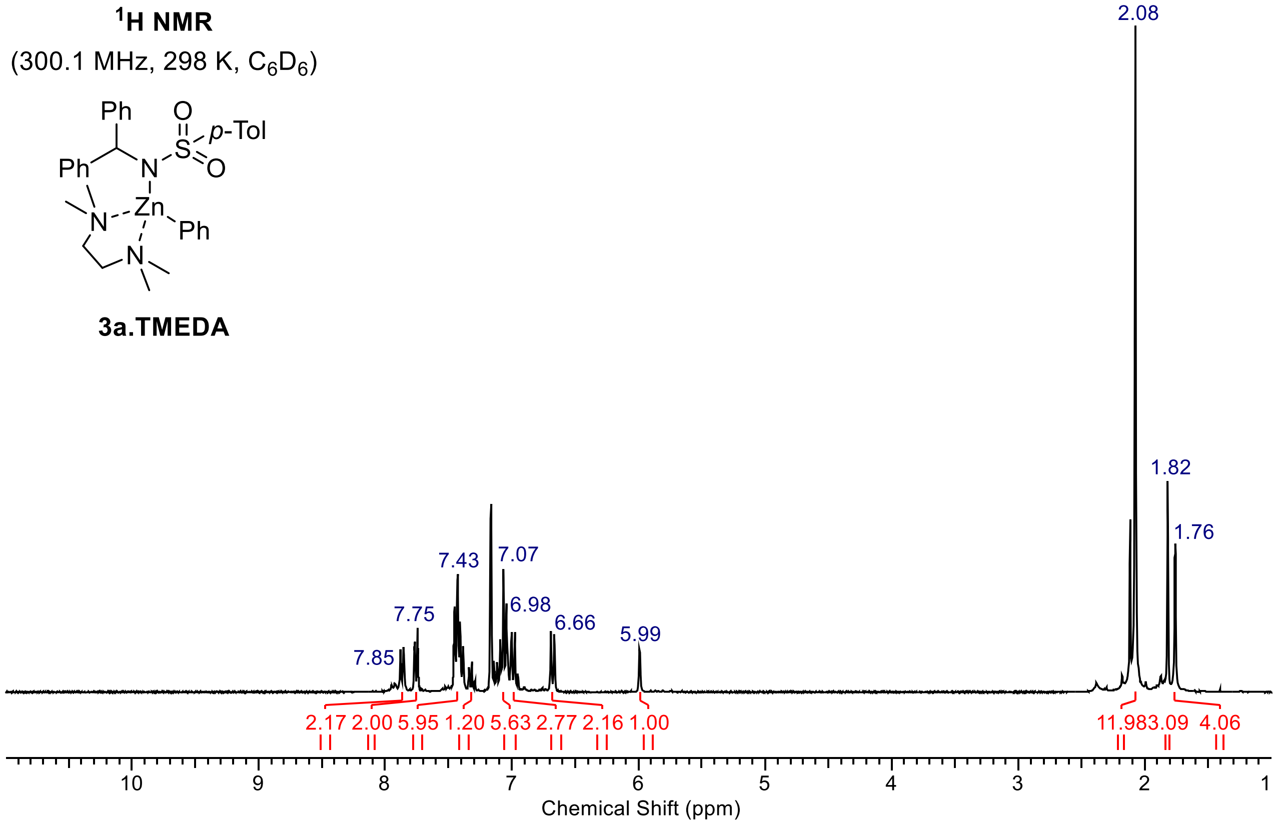

Spectra S60: ^{1}H NMR spectrum of **3a.TMEDA**.

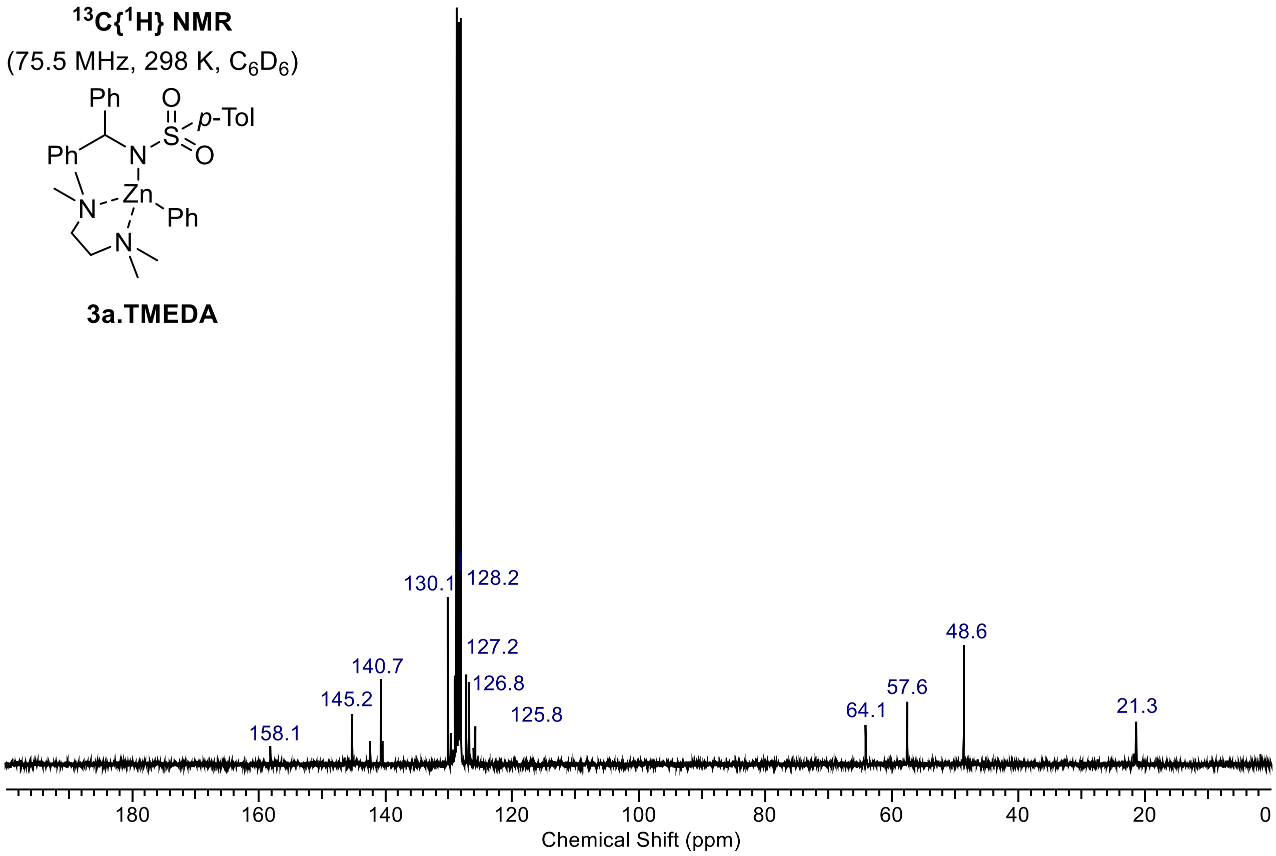

Spectra S61: $^{13}C\{^{1}H\}$ NMR spectrum of **3a.TMEDA**.

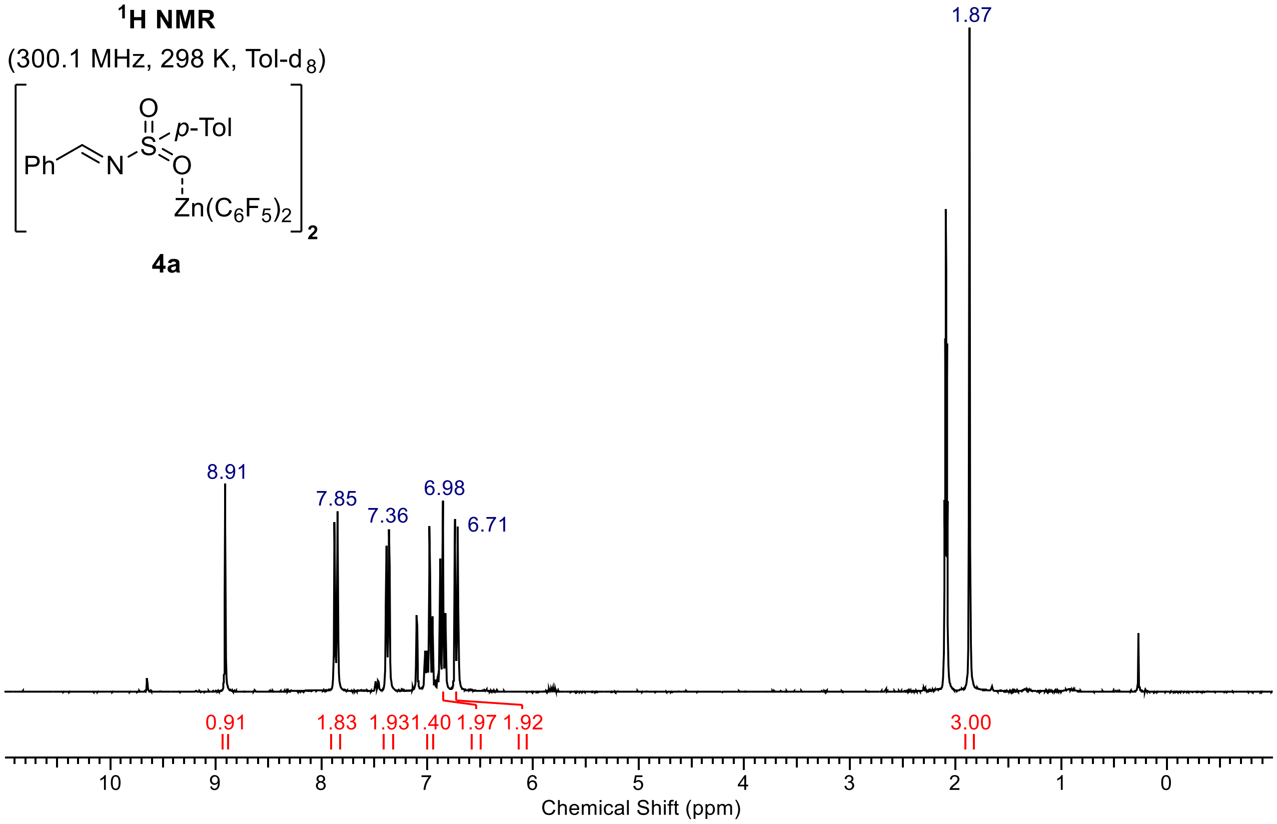

Spectra S62: ^{1}H NMR spectrum of **4a**.

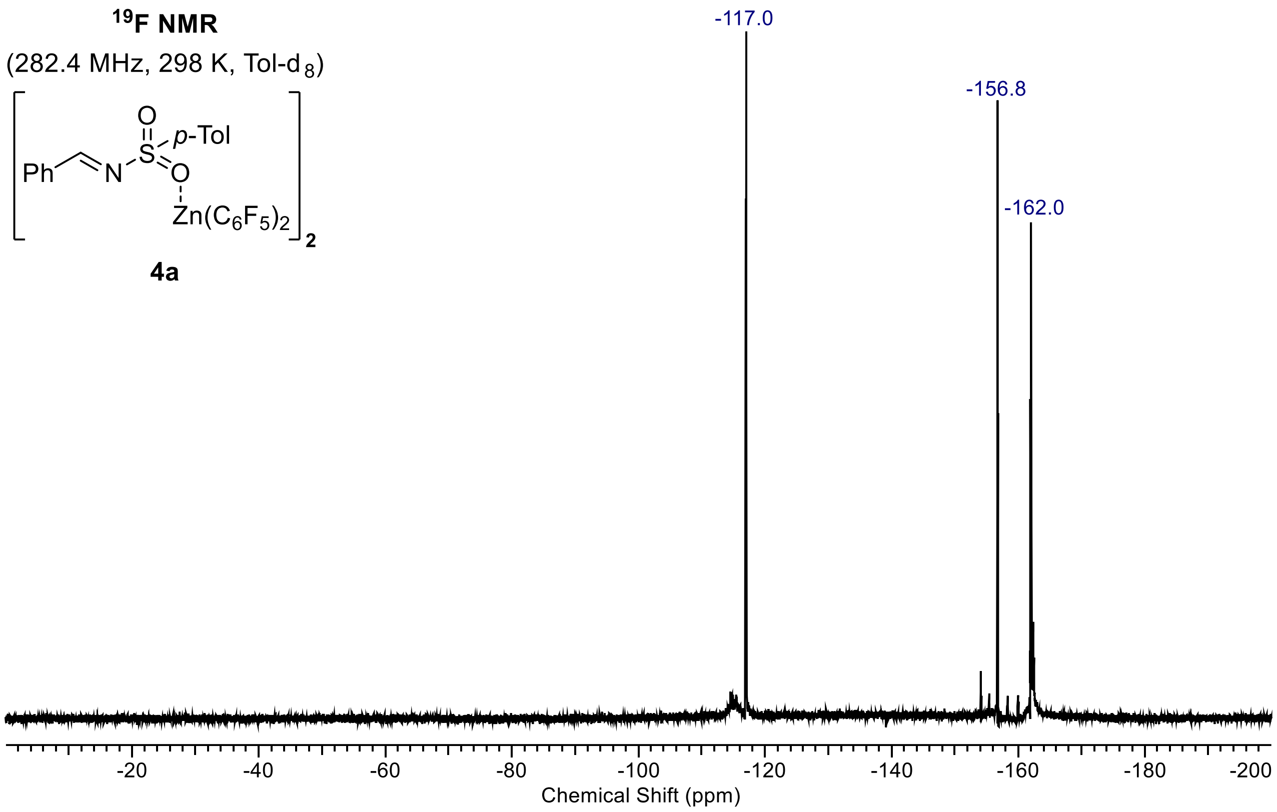

Spectra S63: ^{19}F NMR spectrum of **4a**.

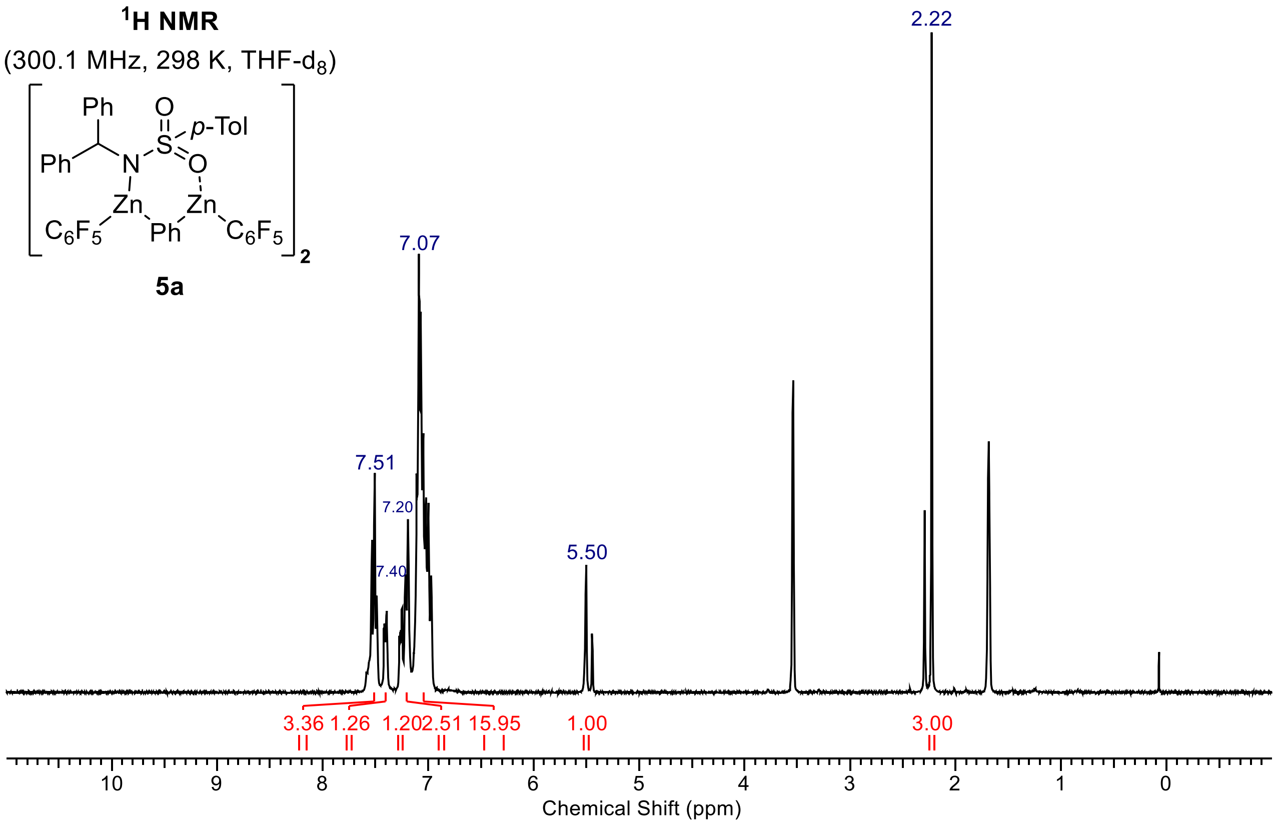

Spectra S64: ^{1}H NMR spectrum of **5a**.

References

1 A. M. Borys, The Schlenk Line Survival Guide, https://schlenklinesurvivalguide.com (Accessed May 2023).

2 A. M. Borys, *Organometallics*, 2023, **42**, 182–196.

3 A. Hernán-Gómez, S. A. Orr, M. Uzelac, A. R. Kennedy, S. Barroso, X. Jusseau, S. Lemaire, V. Farina and E. Hevia, *Angew. Chem. Int. Ed.*, 2018, **57**, 10630–10634.

4 A. M. Borys, J. M. Gil-Negrete and E. Hevia, *Chem. Commun.*, 2021, **57**, 8905–8908.

5 N. Duguet, C. D. Campbell, A. M. Z. Slawin and A. D. Smith, *Org. Biomol. Chem.*, 2008, **6**, 1108.

6 Y. Hu, C. Wang, H. Zhu, J. Xing and X. Dou, *Adv. Synth. Catal.*, 2022, **364**, 531–535.

7 D. Huang, X. Wang, X. Wang, W. Chen, X. Wang and Y. Hu, *Org. Lett.*, 2016, **18**, 604–607.

8 S. Morales, F. G. Guijarro, J. L. García Ruano and M. B. Cid, *J. Am. Chem. Soc.*, 2014, **136**, 1082–1089.

9 C. S. Marques and A. J. Burke, *Eur. J. Org. Chem.*, 2010, **2010**, 1639–1643.

10 Y. Ye, J. Zhang, G. Wang, S. Chen and X. Yu, *Tetrahedron*, 2011, **67**, 4649–4654.

11 G. N. Ma, T. Zhang and M. Shi, *Org. Lett.*, 2009, **11**, 875–878.

12 N. Tokunaga, Y. Otomaru, K. Okamoto, K. Ueyama, R. Shintani and T. Hayashi, *J. Am. Chem. Soc.*, 2004, **126**, 13584–13585.

13 Y. Kim, Y. Il Kwon and S.-G. Kim, *Synthesis*, 2020, **52**, 281–289.

14 Oxford-Diffraction, 2018.

15 G. M. Sheldrick, *Acta Cryst.*, 2015, **A71**, 3–8.

16 G. M. Sheldrick, *Acta Cryst.*, 2015, **C71**, 3–8.

17 O. V. Dolomanov, L. J. Bourhis, R. J. Gildea, J. A. K. Howard and H. Puschmann, *J. Appl. Crystallogr.*, 2009, **A42**, 339–341.
